# An estrogen-regulated long non-coding RNA NCALD promotes luminal breast cancer proliferation by activating GRHL2

**DOI:** 10.1186/s12935-024-03245-0

**Published:** 2024-01-30

**Authors:** Yue Meng, Dianrong Zhou, Ying Luo, Jierong Chen, Hui Li

**Affiliations:** 1 Department of Clinical Laboratory, Guangdong Provincial People’s Hospital (Guangdong Academy of Medical Sciences), Southern Medical University, 106 ZhongShan Road, Guangzhou, 51000 Guangdong China; 2 Zhongshan School of Medicine, Sun Yat-Sen University, Guangzhou, 51000 Guangdong China

**Keywords:** Luminal breast cancer, LncRNA NCALD, LncRNA, GRHL2, ERα

## Abstract

**Purpose:**

Luminal breast cancer (BC) is a prevalent subtype associated with an increased risk of late disease recurrence and mortality. Long noncoding RNAs (lncRNAs) likely play significant roles in regulating tissue-specific gene expression during tumorigenesis. However, the biological function and underlying mechanisms of specific dysregulated lncRNAs in luminal BC remain largely unknown, which has drawn our attention.

**Methods:**

The expression pattern of lncRNA NCALD in luminal BC was predicted and validated in collected tissue samples. Following cell transfection with knockdown of lncRNA NCALD and ESR1 and overexpression of GRHL2 and ESR1, we investigated the interactions among lncRNA NCALD, ESR1, and GRHL2. Additionally, their regulatory functions in luminal BC cell biological processes were studied. Subsequently, a xenograft tumor model was prepared for validation.

**Results:**

Our study identified a specific overexpression of the lncRNA NCALD in luminal BC, which correlated with an unfavorable prognosis. Suppression of lncRNA NCALD or ESR1 led to inhibition of GRHL2 expression, while concurrent overexpression of ESR1 and lncRNA NCALD potentially elevated GRHL2 expression. Mechanistically, ERα may drive the expression of lncRNA NCALD. Furthermore, the 1–151 nt fragment of lncRNA NCALD was found to recruit ERα and interact with its oest-Recep domain located in the promoter region of GRHL2, ultimately inducing GRHL2 transcription.

**Conclusions:**

These findings reveal the involvement of lncRNA NCALD and its specific expression pattern in luminal BC. Targeting lncRNA NCALD could be a potential therapeutic strategy for delaying the progression of BC.

**Supplementary Information:**

The online version contains supplementary material available at 10.1186/s12935-024-03245-0.

## Introduction

Luminal breast cancer (BC) is one of the most common subtypes of BC, accounting for approximately two-thirds of all BC cases [1]. It can be divided into two types: luminal A and luminal B. Among them, luminal A is the most prevalent subtype, characterized by the positive expression of the estrogen receptor (ER) and progesterone receptor (PR). Luminal B, similar to luminal A, is characterized by ER^+^ and PR^+^, but is distinguished by its notably elevated expression of proliferation markers, specifically Ki67. Despite the availability of diverse therapeutic modalities, such as hormonal therapy and chemotherapy, luminal BC continues to present unique challenges [2]. For instance, patients diagnosed with luminal BC exhibit higher rates of late disease recurrence and mortality than those diagnosed with other subtypes. Therefore, despite the better prognosis of patients with luminal BC, their survival rates do not surpass those of other subtypes in the long term (20 years), leading to a significant societal and medical burden [3]. Hence, there is an urgent need to investigate the pivotal regulatory factors and signaling networks involved in luminal BC. Such research would contribute to the identification of novel biomarkers and development of targeted therapeutics, offering a solid scientific foundation.

Long noncoding RNA (lncRNAs) are a class of RNA molecules that have a transcription length of over 200 nucleotides and lack coding ability. LncRNAs play a significant role in various genome regulatory processes, including X chromosome inactivation, genomic imprinting, chromatin modification, transcription, splicing, translation, and degradation [4]. Additionally, they serve as crucial regulators of cell proliferation, differentiation, apoptosis, adhesion, migration, and other biological processes [5]. Some researchers have postulated that cell type-specific lncRNAs may significantly affect cancer progression. Thus, there is significant research interest in characterizing lncRNA involvement in the pathogenesis of luminal BC to identify novel targets and biomarkers for this subtype. Nevertheless, the precise biological function and underlying mechanisms of specific lncRNAs that are transcriptionally dysregulated in luminal BC remain unclear.

ER positivity is a molecular characteristic of luminal BC, with ERs, including ERα and ERβ. ERα plays a role in promoting the progression of luminal BC [6]. To identify evidence of ER-associated oncogenic transcription factor (TFs) binding, researchers conducted transcription factor motif enrichment analyses were conducted on both normal and tumor-specific ER cistromes. The results demonstrated that the GRHL2 DNA-binding motif was one of the most enriched. Previous studies have identified GRHL2 as a potential oncogene associated with the progression of ER^+^ luminal BCs. The absence of the GRHL2 motif in the normal ER cistrome indicates that GRHL2 may act as a transcription factor that cooperates with ER in driving ER^+^ breast tumorigenesis [7–9]. However, it remains unclear how ERα affects GRHL2 expression and its role in the regulatory networks of GRHL2.

In this study, we discovered a significant upregulation of lncRNA NCALD, specifically in luminal BC, which was closely correlated with poor prognosis. In addition, ERα and estrogen transcriptionally upregulate the expression of lncRNA NCALD by binding to the promoters of the SE-lncRNA NCALD. Subsequently, ERα and lncRNA NCALD form a complex that activate the transcription of GRHL2, which ultimately enhances luminal BC proliferation.

## Materials and methods

### Ethics approval and consent to participate

This study was approved by the ethics committee of Guangdong Provincial People’s Hospital. This animal study was approved by the Institutional Animal Care and Use Committee of Guangdong Provincial People’s Hospital.

### Datasets and computational analysis

The Cistrome database browser (http://cistrome.org/db/#/) [10] was used to acquire ESR1 ChIP-seq data for luminal BC cell lines (MCF7:33491, T47D:81169, and ZR-75-1:68858), and SP1 ChIP-seq data for luminal BC cell lines (MCF7 Data ID:64351). The hTFtarget database was used to predict TFs for lncRNA NCALD [11]. RNA-seq analysis was conducted using data downloaded from the GEO database, including datasets GSE70905, GSE58135, and GSE48216 [12]. We utilized the RNA–Protein Interaction prediction (RPISeq) tool (http://pridb.gdcb.iastate.edu/RPISeq/index.html) [13] to predict the protein partners that interact with lncRNA NCALD.

### Sample collection

Forty-eight pairs of frozen tumor and paracarcinoma tissues were collected from patients with BC immediately after surgery at the Department of Breast Surgery, Guangdong Provincial People’s Hospital, between December 1, 2020, and December 31, 2021. The patients provided informed consent, and the clinical processes were approved by the Ethics Committees of Guangdong Provincial People’s Hospital. The histological subgroups of the frozen tissues were determined using immunohistochemistry (IHC) to assess the expression of ERα and PR and fluorescence in situ hybridization (FISH) to evaluate the expression of HER2. All BC specimens were confirmed to have tumor cell percentages > 80%.

### Cell lines and cell culture

MCF7 (RRID: CVCL 0031) and T47D (RRID: CVCL 0553) cell lines were obtained from Guangzhou Cellcook Biotech Co. Ltd. (Guangzhou, China). HCC1954 (RRID: CVCL 1259) was purchased from Cobioer (Nanjing, China). MDA-MB-231 (RRID: CVCL 0062), MDA-MB-468 (RRID: CVCL 0419), SKBR-3 (RRID: CVCL 0033), MCF10A (RRID: CVCL 0598), and ZR-75-1 (RRID: CVCL 0588) were acquired from Procell Life Science & Technology Co., Ltd. (Wuhan, China). All specimens were cultured in standardized media and conditions. All cell lines were subjected to STR authentication. All experiments were performed using mycoplasma-free cells.

### Protein preparation and Western blot assay

Western blot assay was performed as previously described. Briefly, total protein was extracted from cells using RIPA buffer supplemented with protease and phosphatase inhibitors (Beyotime, Beijing). Protein aliquots were separated by SDS-PAGE and visualized using Immobilon Western HRP substrate (Millipore). The primary antibodies used are listed in Additional file 1: Table S1.

### In situ hybridization (ISH) and fluorescence in situ hybridization (FISH)

ISH probes (1. 5′-CCTTTATGACCGAAGATGGAACTGAAATGCCATCCTGTTA-3,’ 2. 5'-ACCATCAGGTTGTAATTGTTCAGATCAGAAATTCCCAAGC-3,’ 3. 5′-AGCCTGGCACAGTTGGGCTTGAAACCATCTGTGTAAAGGG-3′) for the detection of digoxin-labeled lncRNA NCALD were designed and synthesized by BOSTER (Boster Biological Technology Ltd., Wuhan, China). A commercial BC tissue array was obtained from OUTDO (SHANGHAI OUTDO BIOTECH CO., Shanghai, China). Two independent pathologists evaluated the staining score of the lncRNA NCALD to determine its expression as either low or high. FISH was performed on the MCF7 and T47D cell lines. Cells were fixed in 4% formaldehyde for 15 min, washed with PBS, treated with pepsin, and dehydrated using ethanol. A FISH probe was added to the air-dried cells in hybridization buffer at a concentration of 40 nM. Following the hybridization process, the slides were thoroughly washed, dehydrated, and treated with Prolong Gold Antifade Reagent containing DAPI to facilitate the detection of nucleic acids. Fluorescence microscopy (DMI4000B, Leica) was used to observe immunofluorescence on the slides. The plugin colocalization finder of Image J was used for the quantitative analysis of colocalization. To evaluate the colocalization results quantitatively, Pearson's correlation coefficient and overlap coefficient, as proposed by Manders [14, 15], were utilized [16, 17].

### RNAi and cell transfection

Knockdown of lncRNA NCALD in cell lines was accomplished using Ribo lncRNA Smart Silencers, which consist of three pairs of small interfering RNA (siRNA), three pairs of antisense oligonucleotides, and short hairpin RNA (shRNA). ESR1 knockdown cell lines were generated using siRNA and shRNA. Ribo-lncRNA Smart Silencers were developed by RiboBio Co. (Guangzhou, China). The siRNA and shRNA sequences used are listed in Supplementary Table S2. Lipofectamine 3000 transfection reagent (Thermo Fisher, L3000015) was used to transfect 50 nM siRNA or 2 μg shRNA in Opti-MEM (Gibco, #31985070) using reverse transfection techniques. RNA and proteins were harvested at 48 and 72 h, respectively, following siRNA transfection.

### Lentiviral packaging and infection

Lentivirus packaging was performed by Shanghai Genechem (China). To knock down lncRNA NCALD and ESR1, we cloned two validated hairpins (lncRNA NCALD target sequences: ACCGAAGATGGAACTGAAA, ACAGTTGGGCTTGAAACCAT; ESR1 target sequence: TGATCAAACGCTCTAAGAA, TCCGAGTATGATCCTACCA) specifically targeting the human lncRNA NCALD and ESR1 transcript into the hU6-MCS-CMV-puromycin vector. To achieve overexpression of GRHL2 and ESR1, the complete transcripts of these genes were inserted into the CMV-MCS-3FLAG-SV40-puromycin vector. Similarly, the lncRNA NCALD was inserted into the CMV-MCS-SV40-puromycin vector. The infected MCF7 and T47D cells were cultured in selection medium containing 1.0 μg/mL puromycin for 72 h post-infection. The cells were collected for downstream analyses.

### RNA extraction and quantitative real-time polymerase chain reaction (qRT-PCR)

Total RNA was extracted from both BC cell lines and tissues using an RNAeasy Fast Tissue/Cell Kit (TIANGEN, Beijing). Complementary DNA (cDNA) was reverse-transcribed using the RiboSCRIPT mRNA/lncRNA qRT-PCR Starter Kit (Ribobio, Guangzhou, China). SYBR Green PCR Master Mix was used to amplify the cDNA aliquots. GAPDH served as an endogenous control. The sequences of the sense and antisense primers used are listed in Additional file 1: Table S3.

### Cytoplasmic/nuclear RNA fractionation

Cytoplasmic and nuclear RNA fractionation was performed using a cytoplasmic and nuclear RNA purification kit (Norgen, 21,000). RNA was reverse-transcribed and quantified by qRT-PCR. The results for the lncRNA NCALD are illustrated in Additional file 1: Fig. S1.

### Flow cytometry analysis

MCF-7 and T-47D cells stably transfected with shRNA or overexpression vector were collected. We conducted a cell cycle arrest assay using propidium iodide staining followed by flow cytometry as previously described [18]. Three biological replicates were established, and the data collected accurately represented all the experiments.

### Cell proliferation and colony formation assays

A total of 2000 cells were seeded in 96-well plates with 10% FBS-containing culture medium and incubated for 5 days for the CCK-8 assay. Cell viability was assessed daily using a CCK-8 kit. Approximately 1000 cells were seeded into six-well plates and cultured for 2 weeks to conduct the colony formation assay. Colonies were fixed with 4% paraformaldehyde and stained with crystal violet. The ImageJ software was used to count the colonies. Replicates were established for all the assays.

### Annexin V apoptosis assay

After a 15-min staining period at room temperature in the dark using FITC and PE (BD Biosciences), the cells were analyzed by flow cytometry within 1 h (BD Biosciences). Cell apoptosis analysis was performed using FlowJo software (BD Biosciences).

### Xenograft in a nude mouse model

Considering the limited capacity of human cell lines to develop tumors in mice, 5-week-old NOD-SCID mice with severe immune deficiencies were selected. Under the guidance of animal protection organizations and principles of animal welfare, the reduction, refinement, replacement, and responsibility (4R) principle is currently promoted for animal experiments. Based on a previous study [18], five mice that satisfied these criteria were carefully selected for biological replication and 4R. Four different types of MCF7 cells, each with distinct treatment, were injected into female NOD-SCID mice. Tumor volume was measured and evaluated approximately two–three times per week using the formula: length × width^2^ × 0.5. Animals were used in research conducted at the Guangdong Provincial People's Hospital after obtaining approval from the Institutional Animal Care and Use Committee.

### Dual-luciferase reporter assay

The promoter of the lncRNA NCALD was defined as the region located 2 kb upstream of the transcription start site. To investigate the promoter activity of lncRNA NCALD, we generated promoter regions of different lengths: − 1460 to + 100, 1/2, 1/4, and 1/8 of the full-length promoter region. Since the − 1460 to + 100 region contains an ERα-binding site, we constructed a mutant − 1460– + 100 region to investigate the regulatory role of ERα in lncRNA NCALD. The truncated wild-type and mutated sequences of the promoter were cloned into a pGL3-basic vector (Promega) to generate recombinant plasmids. The plasmids were constructed by Synbio Technologies (Suzhou, China). MCF7 and T47D cells were seeded into 24-well plates. After 24 h, 0.2 ng phRL-CMV, 0.5 μg of recombinant plasmids, and 50 nM ESR1 siRNA were transfected using Lipofectamine 3000 (Thermo Fisher Scientific). The cells were harvested 48 h post-transfection and luciferase activity was measured using a dual-luciferase reporter assay system (Promega). Three technical replicates were used for each assay.

### Electrophoretic mobility shift assays (EMSAs) and supershift assays

Biotin labeling was performed on double-stranded DNA oligonucleotides containing the ERα-binding site discovered in the lncRNA NCALD promoter (sense:5′-GACCGGGCCCAGTGACCCGCGGGC-3′), as well as ERα-mutated oligonucleotides (sense:5′-GACCATATACAATTTAAAGCGGGC-3′). Biotin labeling was performed by Viagene Biotech Inc. (Changzhou, China). The assay was performed as previously described. To perform the supershift assay, 1 µg of anti-ERα antibodies and normal goat IgG were preincubated with 4 µg of nuclear extracts in binding buffer for 20 min at ambient temperature before adding the oligonucleotide probes.

### ChIP-qPCR assay

DNA–protein complexes were immunoprecipitated from wild-type MCF7 cells, MCF7 cells with stable knockdown of ERα and lncRNA NCALD, and MCF7 cells treated with an RNA Pol II inhibitor (α-amanitin) using a ChIP assay kit (Millipore, Bedford, MA, USA). The complexes were immunoprecipitated using polyclonal antibodies against ERα (Santa Cruz, sc-8002X) or normal IgG (non-specific DNA-binding control). Immunoprecipitated DNA was purified and subjected to real-time PCR analysis. The primer sequences used for the real-time PCR amplification of GRHL2 were forward (F):5′-TTTCCCAGTCCAAAGGTCAC-3′ and reverse (R):5′-GCTCAGCCTCTTCCTGTCTC-3.’ The primer sequences used for real-time PCR amplification of the lncRNA NCALD were as follows: F:5′-CCAGCTACCCATGAAGCTACTTAG-3′ and R:5′-CATCTCTTGCCTGCCTTCAGA-3.’ The real-time PCR results were analyzed using the 2^−ΔΔCT^ method.

### RNA pulldown assay

Biotin-labeled RNA was transcribed and purified using the Biotin RNA Labeling Mix and T7 RNA polymerase. Biotin-labeled RNA was heated in RNA immunoprecipitation (RIP) buffer for one hour and combined with the cytoplasmic extract. The mixture was subsequently incubated with streptavidin–agarose beads. TRIzol reagent was used to extract the beads for qPCR analysis.

### RNA immunoprecipitation (RIP)

Cells transfected with the specified plasmids were harvested and lysed using lysis buffer. RIP buffer supplemented with magnetic beads was then added to the cell lysates. They were conjugated to the beads either by conjugation with antibodies or anti-IgG as a control. Isolated RNA was digested with DNase I and proteinase K, followed by immunoprecipitation. Subsequently, enrichment of purified RNAs was evaluated using qRT-PCR.

### Statistical analysis

Statistical analysis was conducted using SPSS version 20.0 (SPSS, Inc., Chicago, IL, USA) and Prism version 8.0 (GraphPad Software, Inc., La Jolla, CA, USA) statistical software packages. Student's t-test was used to analyze the experimental data, and all data are presented as the mean ± standard deviation unless otherwise specified. Statistical significance was set at a threshold of *P* < 0.05.

## Results

### Overexpression of lncRNA NCALD is associated with an unfavorable prognosis in luminal BC

**Fig. 1:**
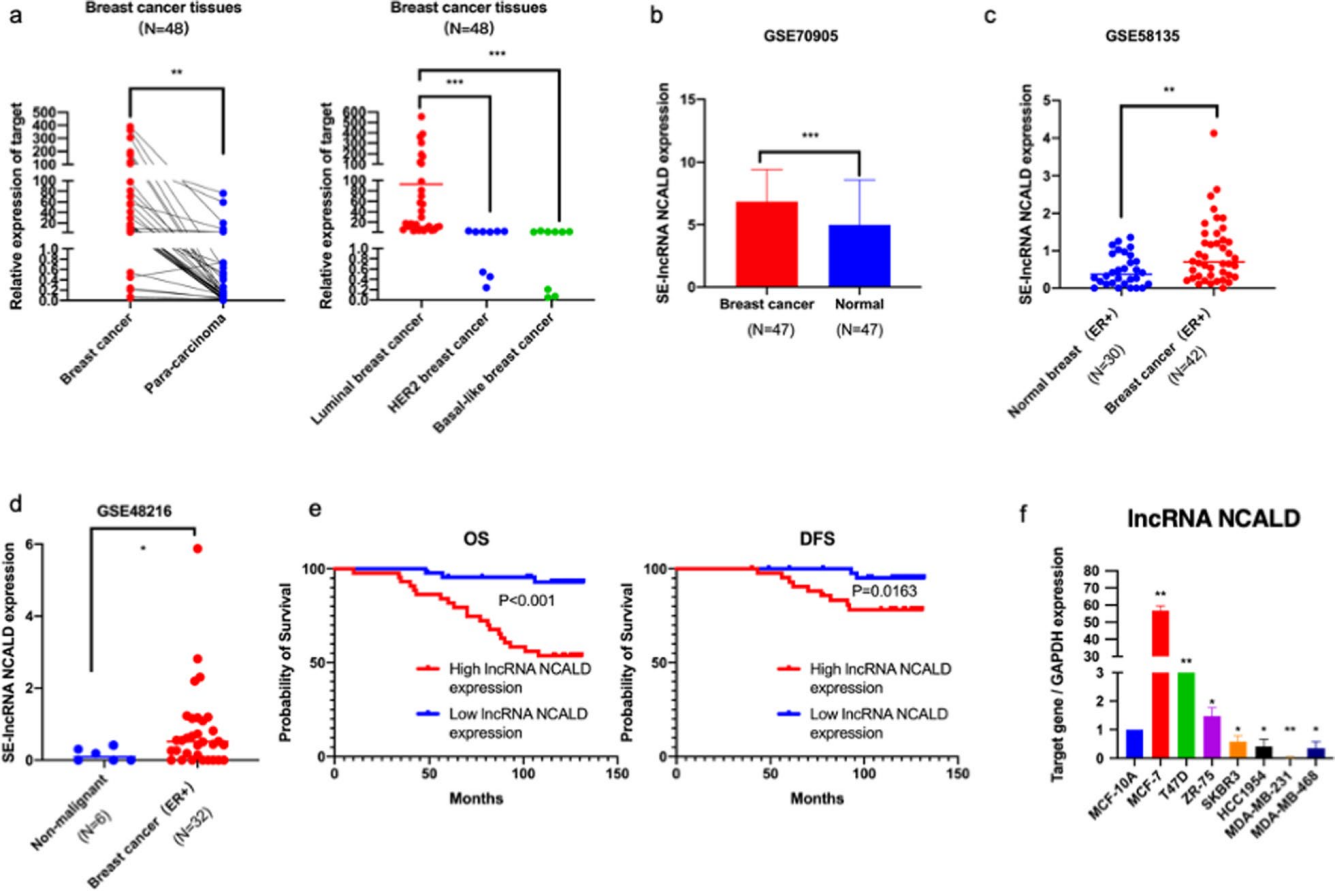
Expression of lncRNA NCALD in both BC tissues and cell lines. Comparison of lncRNA NCALD expression between 48 pairs of BC and para-carcinoma tissues (panel **a**). Expression of lncRNA NCALD in BC and its correlation with clinical features analyzed using data from the GEO database (panels **b–d**). Kaplan–Meier survival curves were constructed to assess overall survival and disease-free survival for patients with luminal BC, stratified according to their lncRNA NCALD expression (panel **e**). The relative expression of lncRNA NCALD in various BC cell lines. The experiments were biologically replicated three times (panel **f**). The data are presented as the means ± SD of three independent experiments. *P < 0.05, **P < 0.01, ***P < 0.001

To identify the significantly overexpressed lncRNAs in luminal BC, we conducted lncRNA microarray analysis using three pairs of luminal BC tissues and their corresponding paracarcinoma tissues. We were particularly interested in the lncRNA NCALD, which was named LNCipedia (https://lncipedia.org/db/search?search_id=ENSG00000254024) and is also known as KB-1562D12.1 or AP001207.3 (Gene ID, ENSG00000254024). This is the only unreported lncRNA transcribed from the super-enhancer region of the luminal BC cell line. It exhibits higher expression in luminal BC tissues than in paracarcinoma tissues. Therefore, it may be a crucial lncRNA driving luminal BC progression (Additional file 2: Table S4). qRT-PCR analysis revealed a significant increase in the expression of lncRNA NCALD in luminal BC tissues compared with that in adjacent non-tumor tissues (Fig. 1a). Furthermore, the increased expression of lncRNA NCALD was validated in luminal BC tissues by ISH staining in a subsequent step. This confirmed that lncRNA NCALD was significantly upregulated in patients with the luminal subtype, characterized by a tumor size greater than 2 cm and ER-positive status. Furthermore, Kaplan–Meier survival analysis and Cox proportional hazards analysis demonstrated that luminal BC patients with high levels of lncRNA NCALD expression exhibited significantly reduced overall and disease-free survival rates. This finding provides independent evidence of the association between high lncRNA NCALD levels and unfavorable outcomes in luminal BC (Fig. 1e, Additional file 1: Table S5–S9). Based on GEO data analysis, we detected upregulation of lncRNA NCALD in BC tissues compared to normal breast tissues, irrespective of tumor subtype (luminal or other types; Fig. 1b–d). Furthermore, we evaluated the expression of lncRNA NCALD in breast cells of different molecular subtypes. Our findings revealed that it was significantly upregulated in luminal BC cells compared to MCF-10A cells, indicating its crucial involvement in luminal BC development (Fig. 1f).

### LncRNA NCALD is transcriptionally activated by ERα

**Fig. 2:**
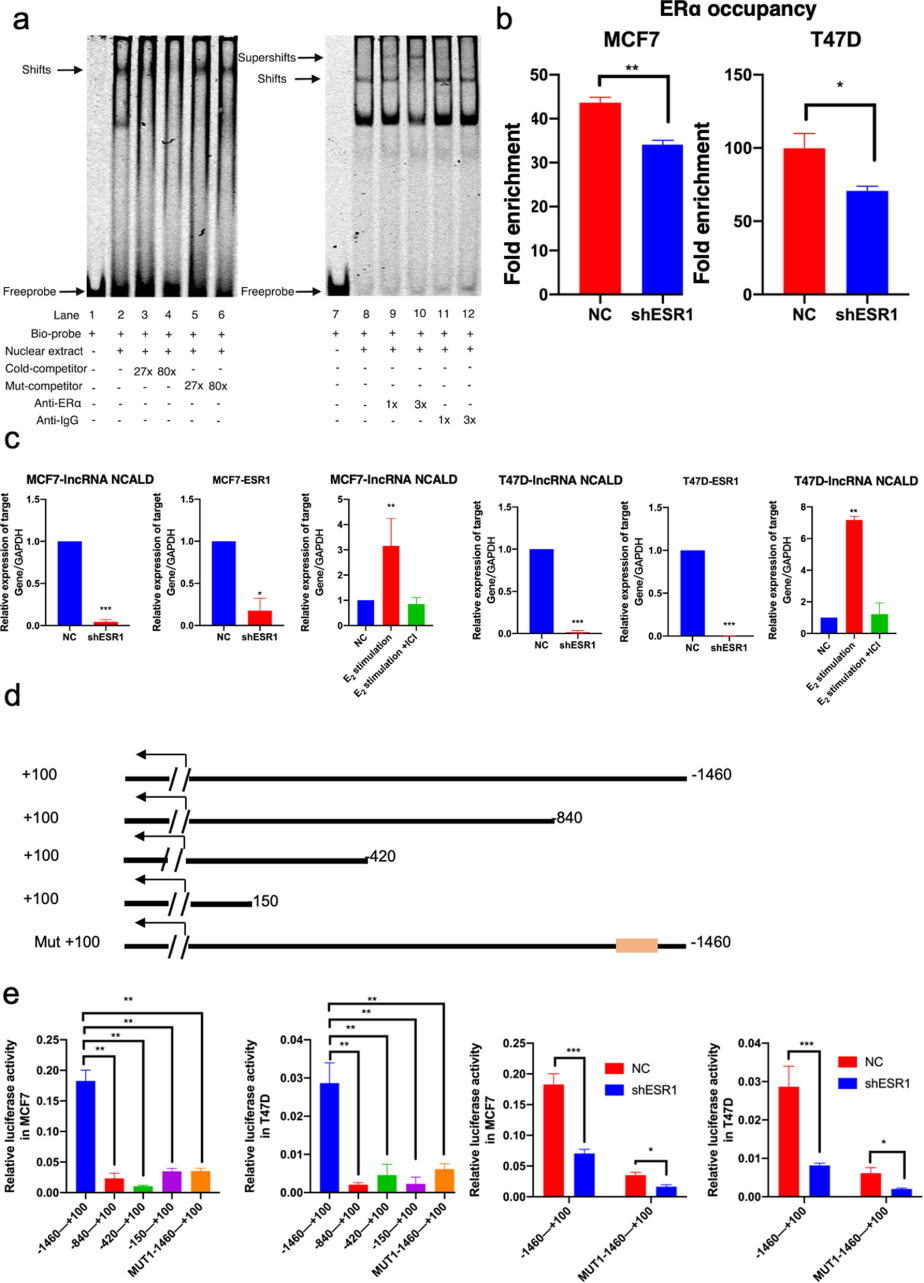
ERα binds directly to the lncRNA NCALD promoter in BC cells, thereby regulating the expression of lncRNA NCALD. Ability of ERα to bind lncRNA NCALD. The DNA–protein complexes were separated through electrophoresis and identified as 'shifts' from the position of the unbound probe. The inclusion of an additional goat anti-ERα antibody resulted in a noticeable 'supershift' and perturbed the 'shift' (panel **a**). A ChIP assay was performed to investigate the binding of ERα to the promoters of lncRNA NCALD in MCF7 and T47D cells using shRNA targeting ERα or control shRNA (*P < 0.05) (panel **b**). All experiments were performed in triplicate. RT-qPCR assay showing the expression of ERα and lncRNA NCALD (panel **c**). All experiments were performed in triplicate. The plasmid details were used in a dual-luciferase reporter assay (panel **d**). The orange box indicates the location of the mutation site for ERα binding, whereas the arrow denotes the transcription start site. Plasmids containing the wild-type lncRNA NCALD promoter and those with a mutated lncRNA NCALD promoter were transfected into shRNA ERα and control cells. All experiments were performed in triplicate. (*P < 0.05, **P < 0.01, ***P < 0.001)

To explore the underlying cause of the selective overexpression of lncRNA NCALD in luminal BC, we initially focused on evaluating TFs that enhanced the expression of lncRNA NCALD. ChIPBase [19] and hTFtarget [11] were used to analyze the promoter region. TCF4, SP1, and ERα exhibited high predictive scores (Additional file 1: Table S10). Furthermore, the ChIP-seq data revealed notable peaks of ERα in close proximity to the transcription start site (TSS), which harbors conserved ERα-binding motifs (Additional file 1: Fig. S2). In contrast, SP1 ChIP-seq data did not show significant peaks near TSS in BC cells (Additional file 1: Fig. S3). We analyzed the expression patterns of ERα and lncRNA NCALD in BC tissues using RT-qPCR. BC tissues with high levels of ERα expressed significantly higher levels of the lncRNA NCALD (Additional file 1: Fig. S2). TCGA data demonstrated a negative correlation between TCF4 and lncRNA NCALD in BC (Additional file 1: Fig. S3). Therefore, ERα is an attractive target for this purpose. To further demonstrate the binding of ERα to the promoter region of the lncRNA NCALD, we performed an EMSA, a supershift EMSA, and a ChIP-qPCR assay. MCF7 and T-47D cells exhibited significant enrichment of ERα occupation in the promoter region of lncRNA NCALD, as shown in Fig. 2a and b. To investigate the regulatory role of ERα in lncRNA NCALD, we used shRNA to knockdown ERα expression in BC cell lines. qPCR analysis demonstrated that shRNA effectively silenced ERα expression. ERα depletion led to a significant reduction in lncRNA NCALD levels (Fig. 2c, Additional file 1: Fig. S4). Furthermore, MCF7 and T47D cell lines were stimulated with estrogen, resulting in high expression of the lncRNA NCALD. However, the estrogen-stimulating effect was significantly reduced by the ICI treatment (Fig. 2c). Moreover, dual-luciferase reporter assays indicated that ERα was capable of binding to the promoter of lncRNA NCALD and regulating its transcription (Fig. 2d, e). In BC cells, ERα binds directly to the promoter region of lncRNA NCALD and governs its expression.

### Suppression of lncRNA NCALD restricts the proliferation of BC cells

**Fig. 3:**
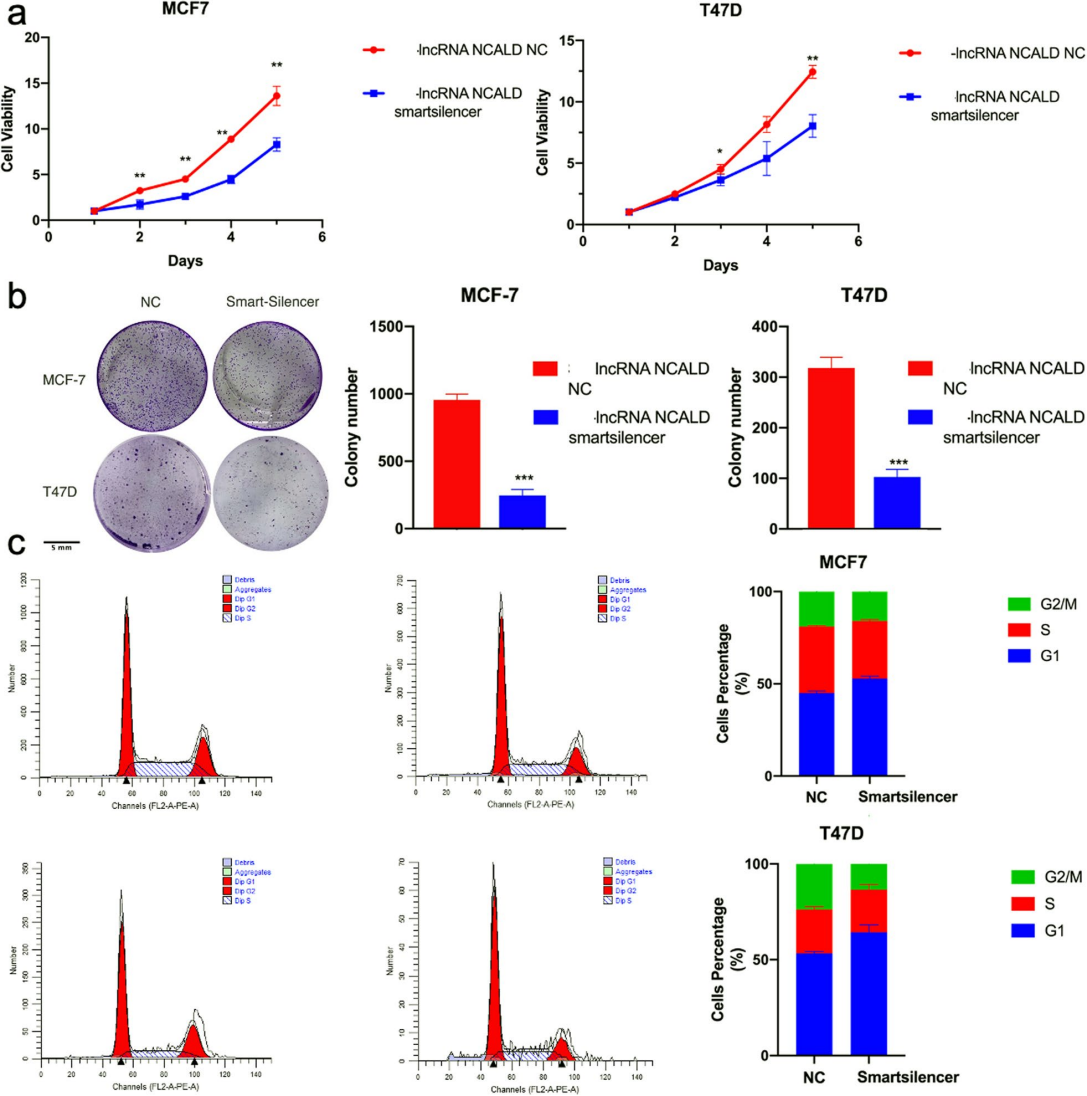
Suppression of lncRNA NCALD restricts the proliferation of BC cells. **a**, **b** CCK8 assay and colony formation assay of T47D and MCF7 cells transfected with smartsilencer or negative control, *P < 0.05, **P < 0.01, ***P < 0.001. All experiments were biologically replicated three times. c. Knockdown of lncRNA NCALD makes the cell cycle arrested at the G1 phase (n = 3, mean ± SD; **P < 0.01)

The biological role of the lncRNA NCALD in luminal BC was also investigated. A smart silencer comprising six pairs of siRNAs was used to suppress the expression of the lncRNA NCALD. This was validated by qPCR assays, demonstrating the successful silencing of the lncRNA NCALD (Additional file 1: Fig. S5). Silencing of lncRNA NCALD led to a significant decrease in the proliferation and colony growth of BC cell lines (Fig. 3a, b). Knockdown of lncRNA NCALD significantly impeded the G1-S transition in luminal BC cells without any impact on apoptosis (Fig. 3c; Additional file 1: Fig. S6).

### LncRNA NCALD regulates the expression of GRHL2, thereby influencing the function of luminal BC

**Fig. 4:**
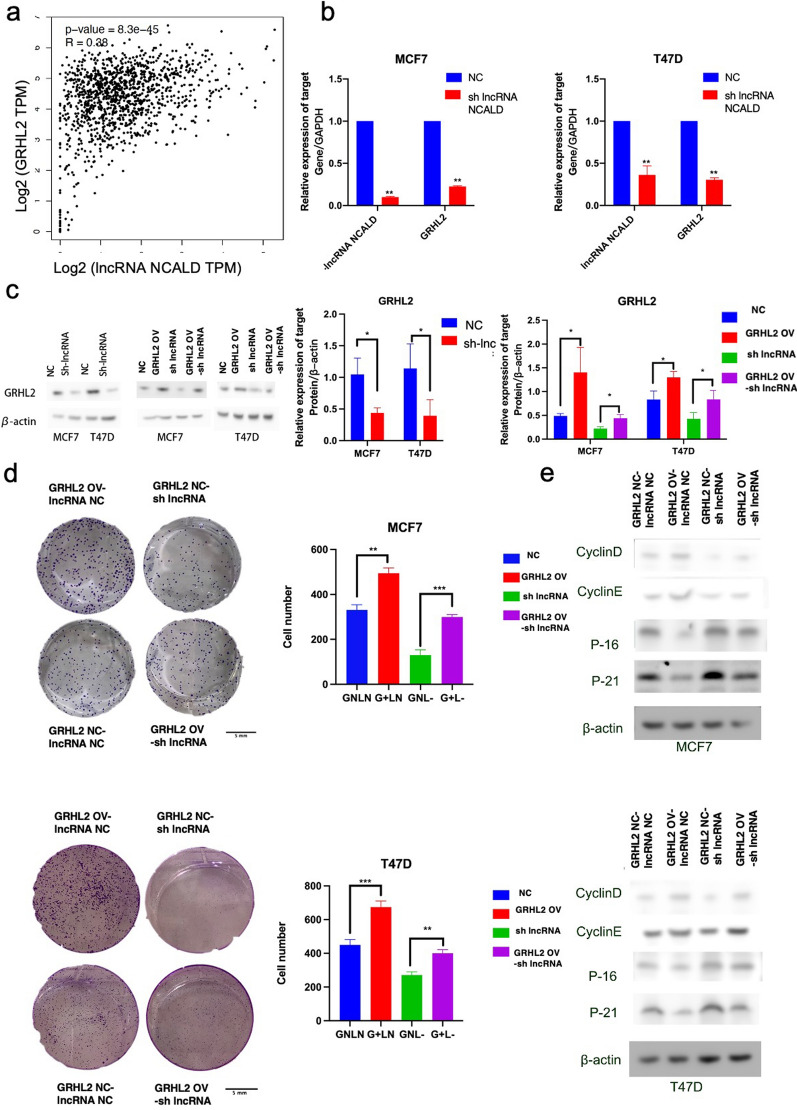
LncRNA NCALD can induce the colony formation of luminal BC cells by regulating the expression of GRHL2. The lncRNA NCALD positively correlated with GRHL2 based on TCGA database data (panel **a**). Knockdown of lncRNA NCALD resulted in the downregulation of GRHL2 expression at the RNA level, (n = 3, mean ± SD; ***P < 0.001) (panel **b**). The level of GRHL2 significantly decreased in cells after lncRNA NCALD knockdown. The level of GRHL2 in cells transfected with GRHL2 overexpressing plasmid and lncRNA NCALD normal control plasmid (GRHL2 OV-lncRNA NC) was significantly higher compared to cells transfected with lncRNA NCALD control plasmid and GRHL2 normal control plasmid (GRHL2 NC-lncRNA NC). The levels of GRHL2 in cells transfected with GRHL2 normal control plasmid and lncRNA NCALD knockdown plasmid (GRHL2 NC-sh lncRNA) were significantly lower compared to those in the GRHL2 NC-lncRNA NC cells. Cells transfected with lncRNA NCALD knockdown plasmid and GRHL2 overexpressing plasmid can restore the depleted level of GRHL2 caused by lncRNA NCALD knockdown (panel **c**). All experiments were biologically replicated three times. Cell proliferation induced by knockdown of lncRNA NCALD was rescued by overexpression of GRHL2 in the colony formation assay (***P < 0.001) (panel **d**). All experiments were biologically replicated three times. The expression of cell proliferation-related proteins cyclin D and cyclin E were reduced, while the expression of p16 and p21 were increased in MCF7 or T47D cell line with knockdown of lncRNA NCALD. Furthermore, the restoration of GRHL2 led to a significant reversal of these expression changes (panel **e**). All experiments were biologically replicated three times

lncRNAs usually affect tumor progression by regulating gene expression [4]. We screened TCGA datasets to identify the target genes of lncRNA NCALD. GRHL2 emerged as the gene most closely resembling the lncRNA NCALD across multiple cancer types (http://gepia.cancer-pku.cn/detail.php?gene=ENSG00000254024). In BC, a positive correlation was observed between the expression of the lncRNA NCALD and GRHL2 (Fig. 4a). Moreover, qPCR and western blot analyses demonstrated a significant decrease in GRHL2 levels upon silencing lncRNA NCALD (Fig. 4b, c; Additional file 1: Fig. S7), indicating that NCALD regulates the transcription of GRHL2. To investigate the impact of lncRNA NCALD on GRHL2 expression and its associated biological function in luminal BC, we generated luminal BC cell lines overexpressing GRHL2 and knocking down lncRNA NCALD. Knockdown of the lncRNA NCALD rescued the expression of GRHL2 (Fig. 4c). GRHL2 overexpression promoted cell proliferation (Fig. 4d, Additional file 1: Fig. S8) and G1-S transition (Additional file 1: Fig. S8) by modulating the expression of relevant signal proteins (Fig. 4e), and this effect could be reversed by silencing the lncRNA NCALD. In summary, lncRNA NCALD can potentially influence the proliferation of luminal BC cells through its regulation of GRHL2 expression.

### LncRNA NCALD interacts with ERα to regulate GRHL2 transcription

**Fig. 5:**
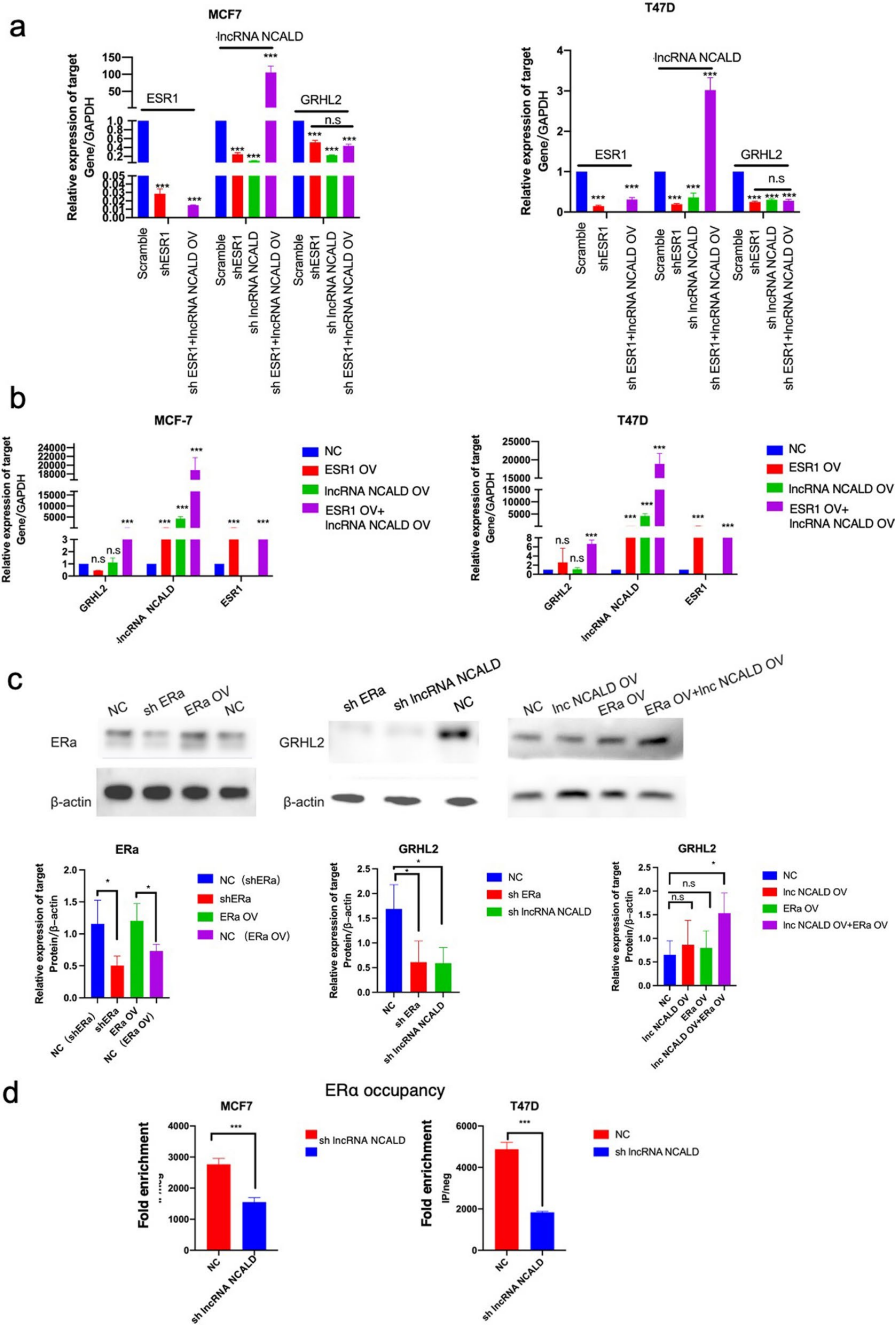
LncRNA NCALD interacts with the ERα to modulate the transcription of GRHL2**.** Knockdown of lncRNA NCALD or ESR1 inhibited expression of GRHL2. However, overexpression of ESR1 did not reverse the level of GRHL2 induced by knockdown of lncRNA NCALD (n = 3, ***P < 0.001) (panel **a**). Concomitant overexpression of ESR1 and lncRNA NCALD led to an upregulation of GRHL2 (n = 3, ***P < 0.001) (panel **b**). Knocking down ESR1 or lncRNA NCALD resulted in the downregulation of GRHL2 protein levels. Only when lncRNA NCALD and ESR1 were simultaneously overexpressed, GRHL2 expression was promoted (n = 3, *P < 0.05) (panel **c**). The ChIP-qPCR assay demonstrated that the knockdown of lncRNA NCALD significantly inhibited ERα binding at the GRHL2 promoter (n = 3, ***P < 0.001) (panel **d**)

lncRNAs frequently regulate adjacent genes by forming complexes with other TFs [20, 21]. Previous studies have indicated that silencing ERα can suppress the expression of GRHL2, while overexpression of ERα fails to increase GRHL2 expression [22]. Therefore, certain lncRNAs are likely to play pivotal roles in this process. In this study, we discovered that suppression of ERα or lncRNA NCALD resulted in the inhibition of GRHL2 expression. Moreover, the expression of GRHL2 was significantly upregulated exclusively upon simultaneous overexpression of ERα and lncRNA NCALD (Fig. 5a–c). Furthermore, ChIP-qPCR assays demonstrated a significant reduction in ERα occupancy at the GRHL2 promoter upon silencing of the lncRNA NCALD (Fig. 5d).

**Fig. 6:**
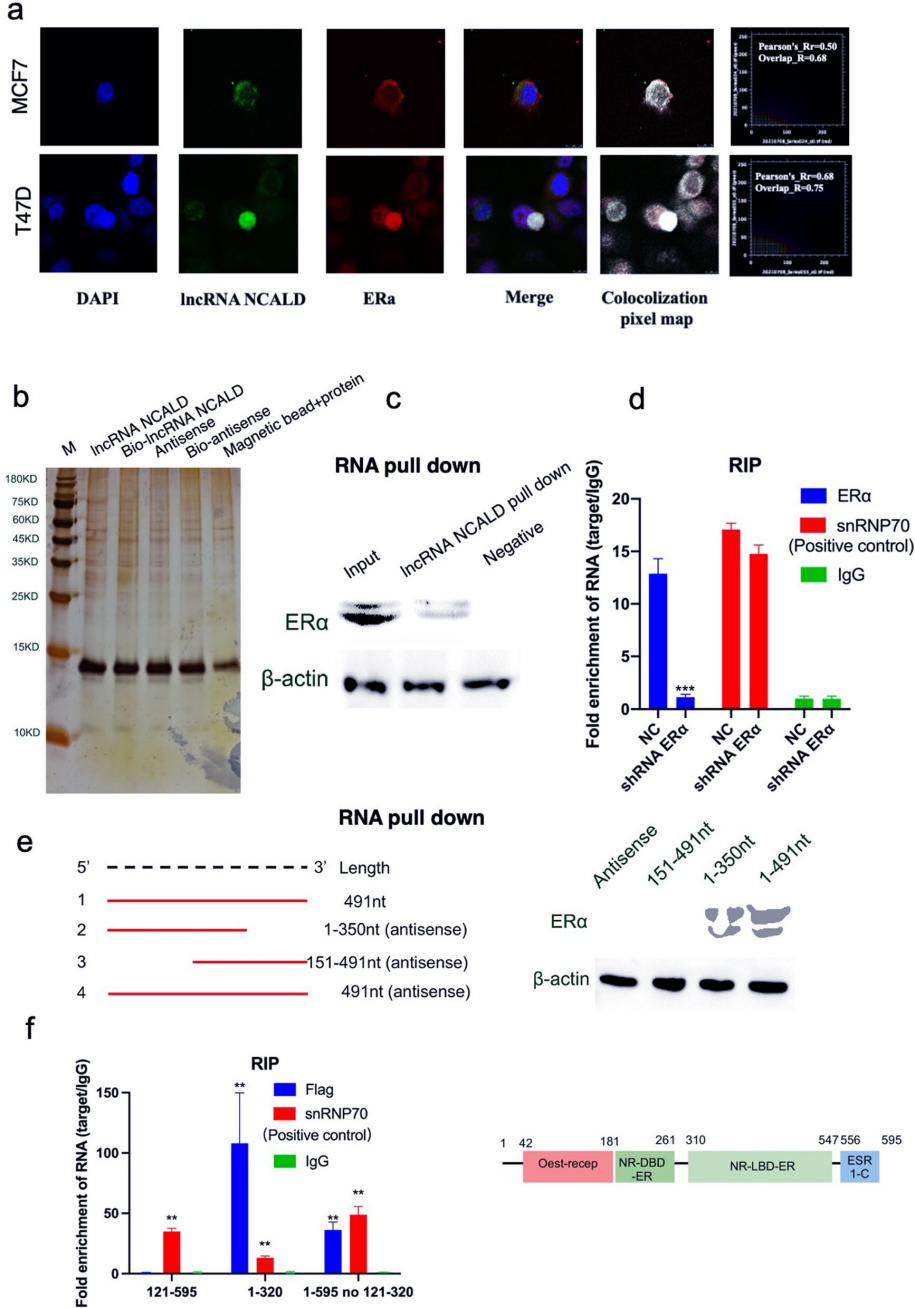
LncRNA NCALD may interact directly with ERα. Cell immunofluorescence assay revealed the colocalization of lncRNA NCALD with ERα in MCF7 and T47D cells (panel **a**). RNA pull-down assay (**b**, **c**) and RIP (**d**) assay indicated a significant interaction between ERα and ncRNA NCALD (n = 3, ***P < 0.001) (panels **b–d**). The results of the RNA pull-down (**e**) and RIP (**f**) assays demonstrated that the fragment spanning nucleotides 1-121 of ERα interacted with lncRNA NCALD at position 1-151 (n = 3, **P < 0.01) (panels **e–f**)

To elucidate the molecular mechanism underlying the regulation of GRHL2 by ERα and lncRNA NCALD, we conducted a cell immunofluorescence assay and predicted RNA–protein interactions. The results revealed a direct interaction between lncRNAs and ERα (Fig. 6a). Furthermore, RIP and RNA pull-down assays indicated that the 1–121 nt fragment of ERα was part of the Oest-Recep domain and could directly interact with lncRNA NCALD at the 1–151 nt position (Fig. 6b–f). Therefore, it is plausible that GRHL2 is regulated simultaneously by NCALD and ERα.

## Discussion

In this study, we discovered that lncRNA NCALD was promoted by ERα. Specific enrichment promotes the proliferation of luminal BCs. Moreover, overexpression of lncRNA NCALD was identified as a standalone prognostic indicator in luminal BC. Moreover, it has been demonstrated that the lncRNA NCALD promotes proliferation in luminal BC by interacting with ERα, which stimulates the transcription of GRHL2.

Cell-type-specific lncRNAs play pivotal roles in cancer progression [23]. For instance, six lncRNAs are specifically expressed in hepatocellular carcinoma (HCC) cells but not in the liver or other normal tissues. These lncRNAs have been found to influence HCC [24]. Furthermore, researchers have investigated the subtype-specific and cell-type-specific expression of lncRNAs in BC through bioinformatics analysis [25]. However, their potential clinical value remains unexplored. In this study, we used tissues and cell lines representing diverse molecular subtypes of BC to identify potential lncRNAs. Our findings revealed that lncRNA NCALD was specifically overexpressed in luminal BC. Furthermore, enrichment of lncRNA NCALD was associated with high-grade BC. It also functions as an independent prognostic marker for luminal BC, indicating its potential as a useful marker for this subtype of BC. The potential reasons for this expression pattern may be related to two factors. On the one hand, previous studies suggested that super enhancers play prominent roles in driving the expression of cell-type-specific genes. It was observed that a super enhancer cluster in luminal and her2 breast cancers but not in normal and basal-like breast cancers was discovered within lncRNA NCALD’s promoter and gene body. The ChIA-PET data of MCF7 cells suggested that the super enhancer interacted with the promoter of lncRNA NCALD (data not shown). Furthermore, in cancer, lncRNA expression is often regulated by the occupancy of subtype-specific TFs at their promoter regions. For instance, the regulation of DSCAM-AS1 by ERα renders it specific to cell lines expressing ERα and exerts an impact on cellular functions [26]. Furthermore, FOXA1 and STAT3 have been shown to elevate the expression of lncRNAs in luminal BC [27, 28]. To identify the master transcription factor of lncRNA NCALD, the promoter region of lncRNA NCALD was analyzed using ChIPBase [19] and hTFtarget [11]. The prediction score for ERα was high, and its expression was positively correlated with lncRNA NCALD. ERα, a molecular characteristic of luminal BC, primarily affects cellular function via two pathways. It can activate MAPK, PI3K, and other signaling pathways to enhance cell proliferation and suppress apoptosis [29]. Furthermore, it serves as a critical transcription factor capable of activating oncogene transcription by binding to the ER element [30]. In our study, we identified a novel finding that the lncRNA NCALD is responsive to estrogen stimulation, and its transcription is activated by ERα. These results provide an explanation for the reduced expression of the lncRNA NCALD in HER2 or Basal-like cells, which is potentially caused by the absence of ERα regulation.

As lncRNA NCALD is associated with the clinical characteristics and prognosis of luminal BC, we propose that it may exert an impact on the cellular functions of luminal BC. The results indicated that the lncRNA NCALD enhanced cell proliferation in vivo and in vitro. This finding was consistent with the GEO data, which demonstrated that the expression of lncRNA NCALD was elevated in BC tissues compared to that in non-malignant or normal breast tissues.

Our ultimate objective was to identify the specific target gene of lncRNA NCALD and elucidate its regulatory mechanisms. We analyzed the correlation between the lncRNA NCALD and other genes. We observed a positive correlation between GRHL2 and NCALD lncRNAs. While the correlation between GRHL2 and lncRNA NCALD is not particularly strong, both genes are specifically expressed in luminal BC and exhibit a close relationship with ERα [31]. Furthermore, our findings implied that the overexpression of GRHL2 stimulated cell proliferation in luminal BC, which was in line with previous studies demonstrating the subtype-specific role of GRHL2 in BC [10].

Furthermore, our findings revealed that inhibiting the expression of the lncRNA NCALD reversed the cellular effects caused by the overexpression of GRHL2. GRHL2 is a tumorigenic ER-cooperating transcription factor [22]. Several studies have indicated that downregulation of ERα can suppress the expression of GRHL2. However, the rationale behind the lack of upregulation of GRHL2 expression upon ERα overexpression has not been investigated [22, 31]. Our study demonstrated that knockdown of either lncRNA or ERα downregulated GRHL2, whereas only the concurrent overexpression of lncRNA NCALD and ERα upregulated GRHL2 expression. The lncRNA NCALD serves as a scaffold for recruiting ERα to the GRHL2 promoter. Moreover, we discovered interactions between the lncRNA NCALD and ERα regions, which suggested that in the future, we can develop inhibitors targeting lncRNA NCALD fragment, which destroy its scaffold effect and prevent ERα from binding to GRHL2 promoter region, and thus inhibit the expression of GRHL2 to provide a new therapy for luminal breast cancer.

Some limitations of the study include the relatively small sample size and further validation in larger cohorts and in vivo models would be necessary in our future research. Besides, the expression of lncRNA NCALD in serum of luminal breast cancer patients was also unclear and its implications for liquid biopsy applications should be explored in the following study.

In summary, we discovered a new lncRNA, NCALD, that exhibited specific upregulation in luminal BC and displayed a positive correlation with poor prognosis. The unique expression pattern of the lncRNA NCALD can be attributed primarily to ERα stimulation. lncRNA NCALD facilitates cell proliferation by regulating GRHL2. It can also directly interact with ERα and bind to the GRHL2 promoter, consequently affecting its expression.

### Supplementary Information

**Additional file 1.** Supplementary figures and tables

**Additional file 2.** Raw data of microarray

## Data Availability

All data generated or analyzed during this study are included in this published article and its supplementary information files.
