# Supplementary material for: An estrogen-regulated long non-coding RNA NCALD promotes luminal breast cancer proliferation by activating GRHL2
Source: Cancer Cell Int. 2024 Jan 30;24:49. doi: 10.1186/s12935-024-03245-0 (PMC10829383; doi:10.1186/s12935-024-03245-0)
Supplement: Supplementary file 1 — Additional file 1. Supplementary figures and tables [file 12935_2024_3245_MOESM1_ESM.pdf]

Table S1. The primary antibodies

Name	Lot	Corporation
ERa	sc-8002X	Santa Cruz
p16	92803S	CST
p21	2947S	CST
E-cadherin	ab133597	Abcam
N-cadherin	13116	CST
Cyclin E2	4132	CST
Cyclin D1	bs-20596R	Bioss
Snail	3879	CST
GRHL2	A10153	Abclonal

Table S2. The siRNA and shRNA sequences

**Product name**	**Target sequence**
**SE-lncRNA NCALD Smart Silencer**	**ACAGTTGGGCTTGAAACCAT**
	**GCCCTGTCATTGGGAATAAG**
	**GGACTAATAATGCTGACCTC**
	**GAGAGATGCAAGATTGAAA**
	**ACCGAAGATGGAACTGAAA**
	**GCAAGAGAATTGAGTAAGA**
**ESR1 siRNA**	TGATCAAACGCTCTAAGAA
	TCCGAGTATGATCCTACCA
	TACTGTTTGCTCCTAACTT
SE-lncRNA NCALD shRNA	**ACCGAAGATGGAACTGAAA**
**ESR1 shRNA**	TACTGTTTGCTCCTAACTT

Table S3. The primers of RT-qPCR

ESR1	F:5-CGTCGATTATCTGAATTTGGCCT-3
	R:5-CCCACTCAACAGCGTGTCTC-3
GRHL2	F:5-TCAATACCCGAAGAGCCTACA-3
	R:5-CTTGGCTGTCACTTGCTTTGC-3
SE-lncRNA NCALD	**F:5-CGAAGATGGAACTGAAATGCC-3**
	R:5-CTGGATGAGGCTCCCTGAAG-3
GAPDH	F:5-GAACGGGAAGCTCACTGG-3
	R:5-GCCTGCTTCACCACCTTCT-3
c-MYC	F:5-GGCTCCTGGCAAAAGGTCA-3
	R:5-CTGCGTAGTTGTGCTGATGT-3

Table S4. Raw data of microarray（showed in Supplemental Videos and Spreadsheets）

Table.S5 Relevance analysis of lncRNA NCALD expression in patients with breast cancer (n = 134)

Characteristics	Total	SE-lncRNA NCALD		*P*
		Low	High	
Age (Years)				0.788
>60	41	27	14	
≤60	93	59	34	
Lymph node metastasis				0
Negative	57	44	13	
Positive	77	42	35	
Tumour size (cm)				0
>2	70	27	43	
≤2	64	59	5	
AJCC stage				0.39
I\II	90	60	30	
III\IV	44	26	18	
Molecular subtype				0
Basal-like	29	27	2	
HER2	16	14	2	
Luminal	89	45	44	
ER status				0
High	47	3	44	
Low	87	83	4	
PR status				0.003
High	45	21	24	
Low	89	65	24	
HER2 status				0.807
High	38	25	13	
Low	96	61	35	

Table.S6 Prognostic factors of overall survival in patients with luminal breast cancer by univariate analysis.

Parameter	N	Cumulative survival rates (%)		Mean survival time (Mo)	HR	95% CI	*P*
		3 years	5 years				
Age							
≤60	59	-	-	129.3			
>60	30	-	93.3	114.2	6.354	1.681–24.02	0.006
lncRNA NCALD level							
Low	45	-	-	130.2			
High	44	-	93.0	118.1	5.346	1.15–24.77	0.032
Lymph node metastasis							
No	48	-	-	129.4			
Yes	41	-	92.7	119.2	11.93	1.53–93.21	0.018
Tumour size							
≤2cm	34	-	-	129.5			
>2cm	55	-	94.5	120.8	2.87	0.62–13.27	0.178
AJCC							
1-2	64	-	96.8	127.1			
3-4	25	-	95.8	116.4	3.35	1.02–10.97	0.046
ER status							
Low	42	-	-	130.1			
High	47	-	93.3	118.5	4.81	1.04–22.27	0.045
PR status							
Low	47	-	95.7	123.0			
High	42	-	97.6	125.3	0.95	0.29–3.10	0.927
HER2 status							
Low	67	-	95.5	123.1			
High	22	-	-	126.4	0.70	0.15–3.24	0.648

Table S7 Multivariate analysis of overall survival in luminal breast cancer using the Cox proportional hazards model.

Parameter	N	HR	95%CI	*P*
Age				
≤60	59			
>60	30	7.59	1.62-35.65	0.01
lncRNA NCALD level				
Low	45			
High	44	30.122	5.16-175.79	<0.001
Lymph node metastasis				
No	48			
Yes	41	36.91	4.22-322.70	0.001
AJCC				
1-2	64			
3-4	25			0.167
ER status				
Low	42			
High	47			0.837

Table.S8 Prognostic factors of disease-free survival in luminal breast cancer patients by univariate analysis.

Parameter	N	Cumulative disease -free rates (%)		Mean disease-free time (mo)	HR	95% CI	*P*
		3 years	5 years				
Age							
≤60	59	96.6	-	118.2			
>60	30	96.7	83.2	107.4	1.741	0.76–3.98	0.189
lncRNA NCALD level							
Low	45	-	-	127.8			
High	44	-	81.8	101.3	8.453	2.51–28.48	0.001
Lymph node metastasis							
No	48	93.7	-	116.1			
Yes	41	-	87.8	112.7	1.524	0.67–3.48	0.317
Tumour size							
≤2cm	34	97.1	-	129.5			
>2cm	55	96.4	87.3	120.8	3.14	1.07–9.23	0.038
AJCC							
1-2	64	-	98.4	122.5			
3-4	25	-	92.0	94.1	5.19	2.24–12.19	<0.001
ER status							
Low	42	-	-	130.1			
High	47	93.6	82.8	118.5	7.48	12.22–25.20	0.001
PR status							
Low	47	-	91.4	117.6			
High	42	95.2	85.6	110.8	1.57	0.69–3.58	0.284
HER2 status							
Low	67	97.0	91.0	116.4			
High	22	95.5	-	107.7	1.79	0.75–4.22	0.184

Table. S9 Multivariate analyses of disease-free survival in luminal breast cancer using the Cox proportional hazards model.

Parameter	N	HR	95%CI	P
SE-lncRNA NCALD level				
Low	45			
High	44	6.913	2.035–23.482	0.002
Tumour size				
≤2cm	34			
>2cm	55			0.917
AJCC				
1-2	64			
3-4	25	4.07	1.75–9.48	0.001
ER status				
Low	42			
High	47			0.741

Table S10. The result of motif analysis of the promoter region using ChIPBase and haTFtarget TF

Source	Sequence name		Star	Stop	Strand	Score	P value	Q value	Matched Motif
TCF4	hTFtarget	NC_000008.11:101492399-101494499	194	215	+	20.5526	7.69E-08	1.87E-05	GGCGCGAGCGGGCGAGCGAGCG
SP1	hTFtarget	NC_000008.11:101492399-101494499	1033	1054	-	17.803	5.29E-07	0.000836	CGGGGGAGGGAGGCGGGGGAGG
ESR1	hTFtarget	NC_000008.11:101492399-101494499	1028	1052	-	17.4328	5.37E-07	0.00214	GGGGAGGGAGGCGGGGGAGGGGGGA

Figure S1. Cytoplasmic/nuclear RNA fractionation of SE-lncRNA NCALD. MALAT1 and H19 were the positive control of genes located at nuclear and cytoplasmic regions. Three biological replicates were performed, and the data are representative of all experiments.

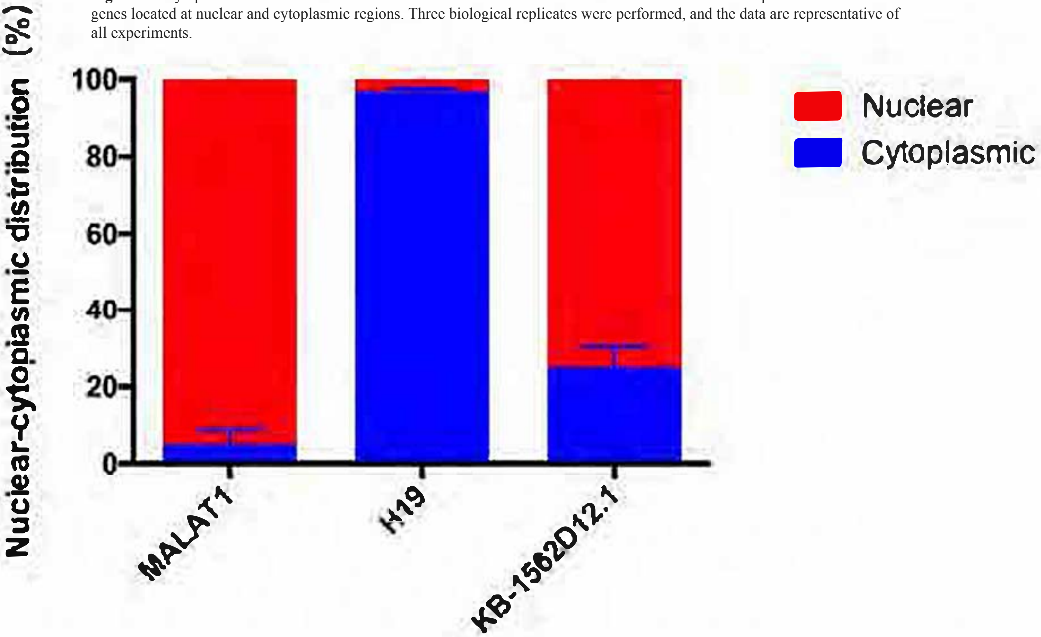

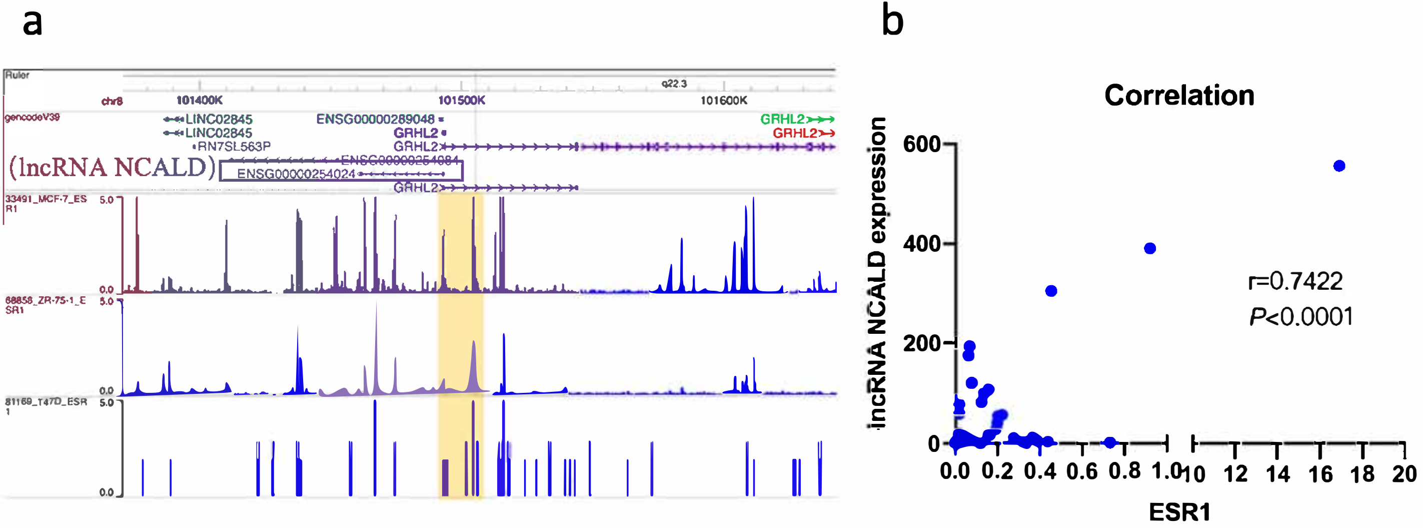

Figure S2. a. ChIP-seq data showed significant peaks of ERα near the TSS(Yellow rectangle represents significant peaks of ERα). b RT–qPCR analysis of breast cancer tissues was conducted to analyze the expression patterns of ERα and lncRNA NCALD.

a

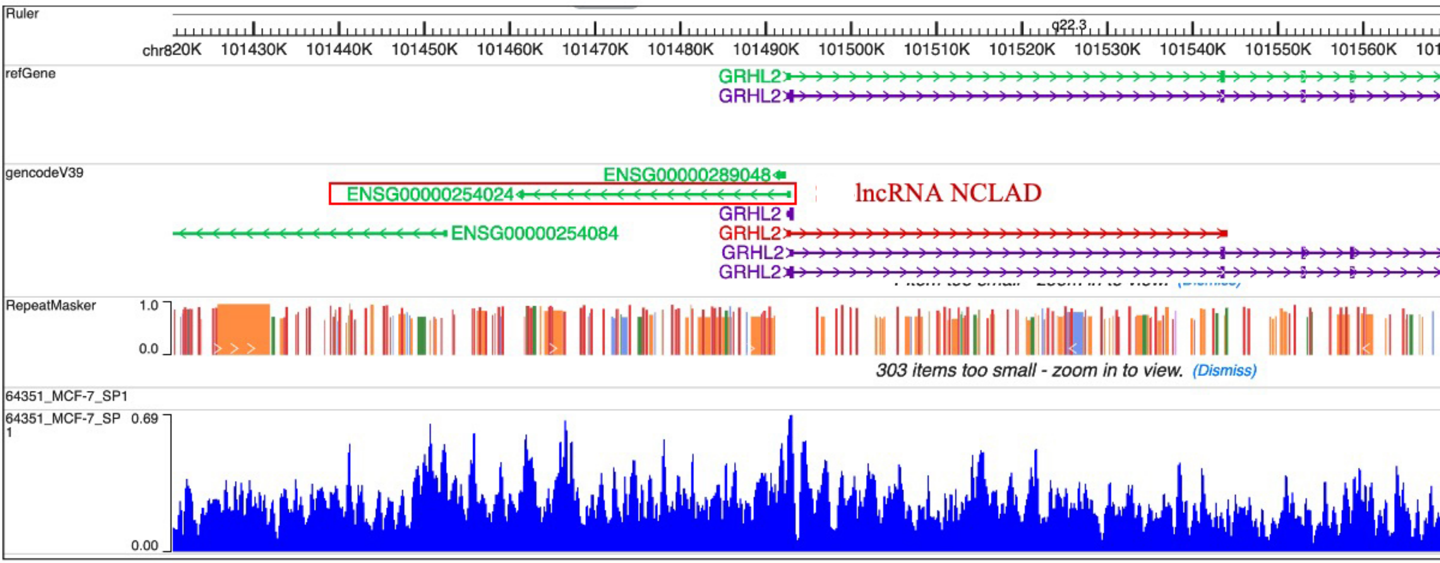

b

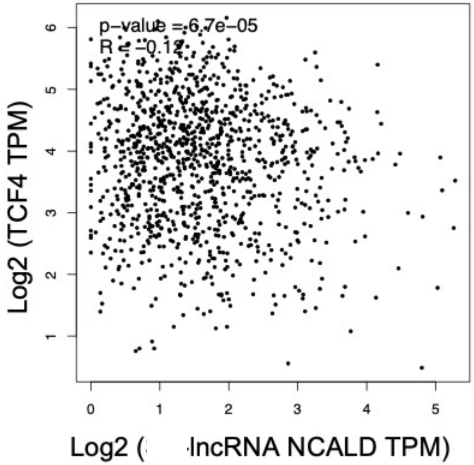

Figure S3. SP1 and TCF4 were not candidate transcription factors of lncRNA NCALD. a. SP1 ChIP-seq data showed low signal (rpm<0.69) near TSS of lncRNA NCALD in MCF7 cell. The y-axis represents reads per million (rpm) of ChIP-seq. b. The correlation between TCF4 and lncRNA NCALD was negative in breast cancer (r=-0.12)

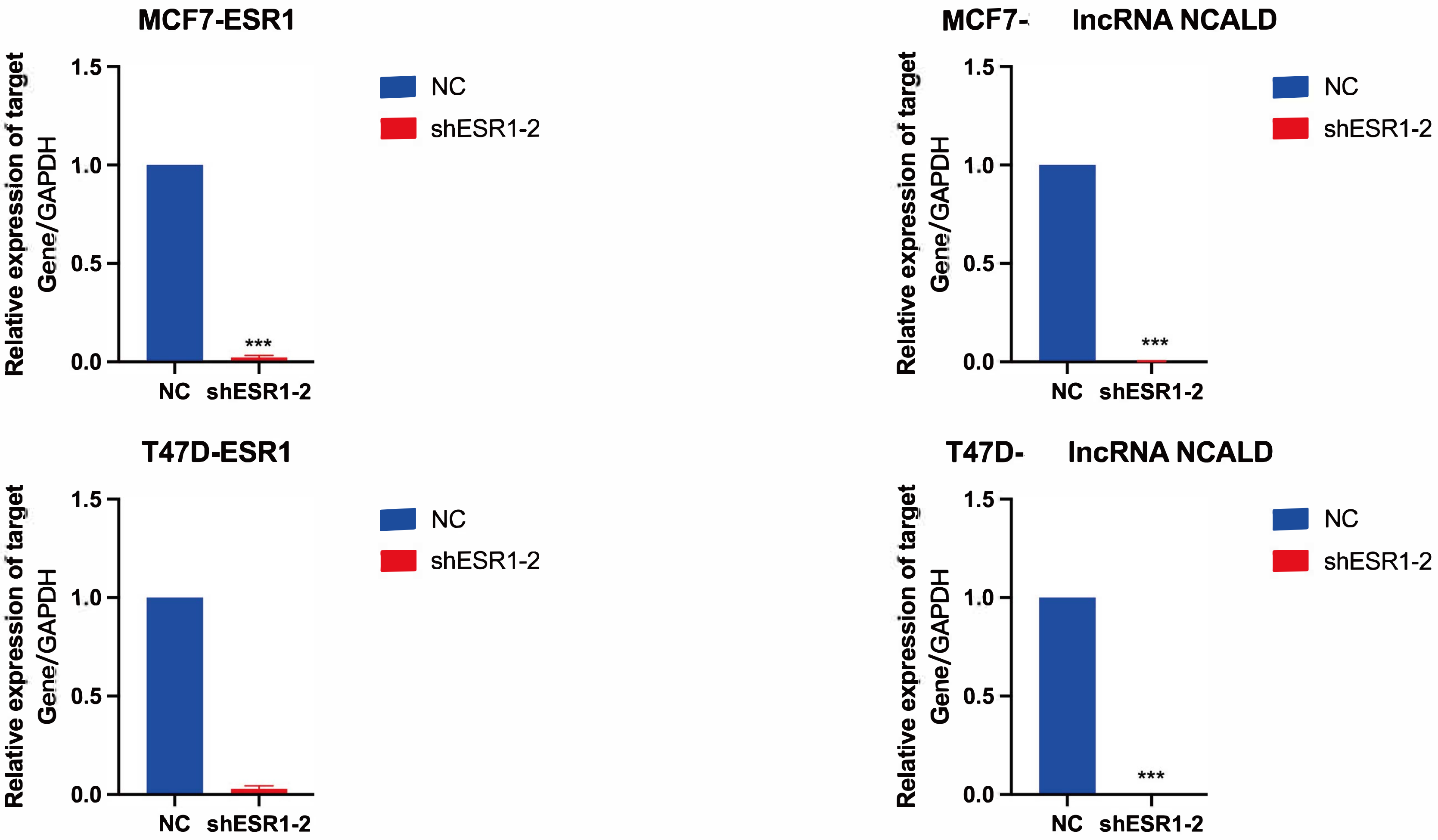

Figure S4.RT-qPCR assay showed that the expression levels of ERα and lncRNA NCALD in normal control cells were lower than that in the shESR1-2 cells. All experiments were biologically replicated three times.

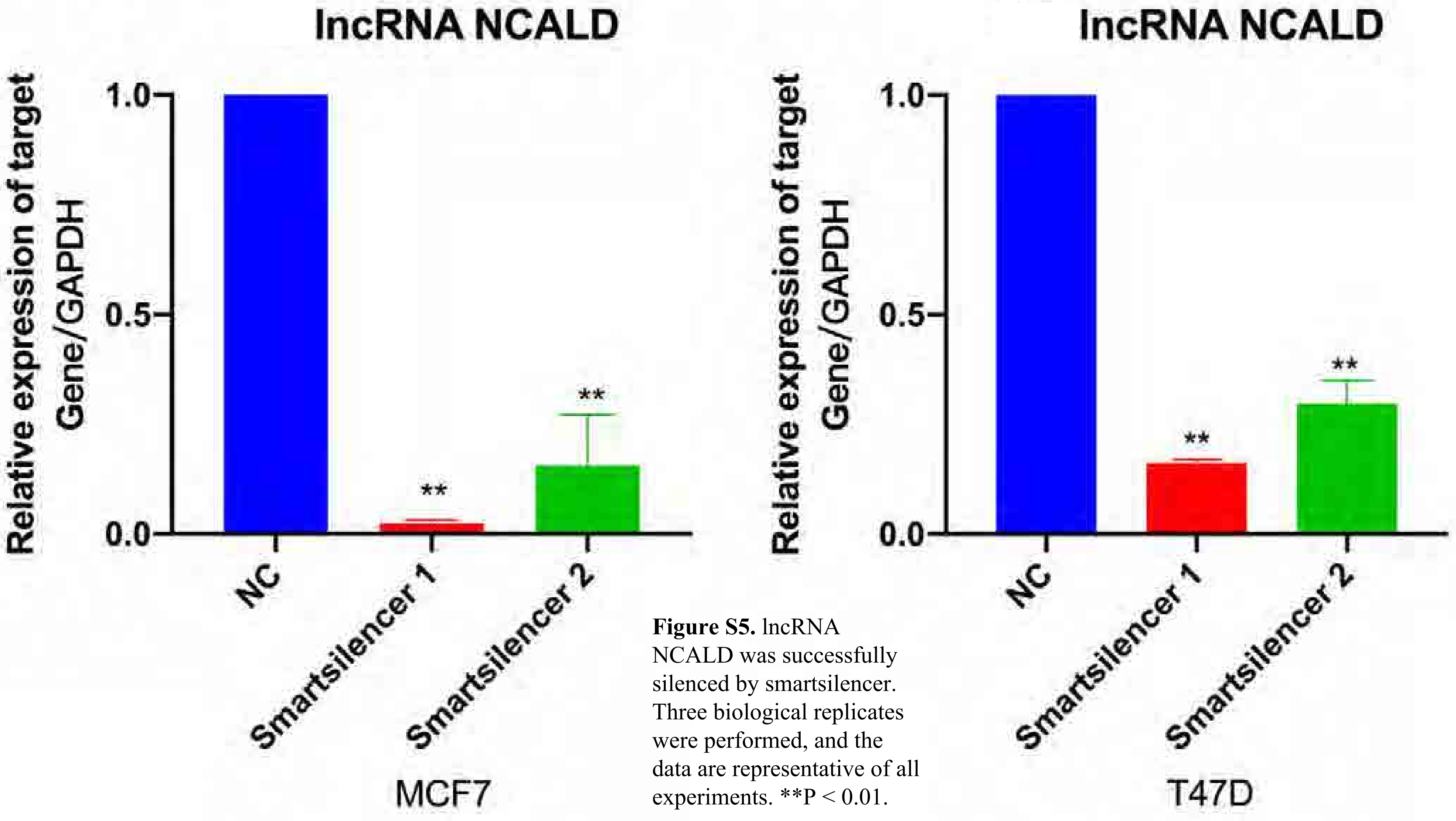

Figure S5. lncRNA NCALD was successfully silenced by smartsilencer. Three biological replicates were performed, and the data are representative of all experiments. **P < 0.01.

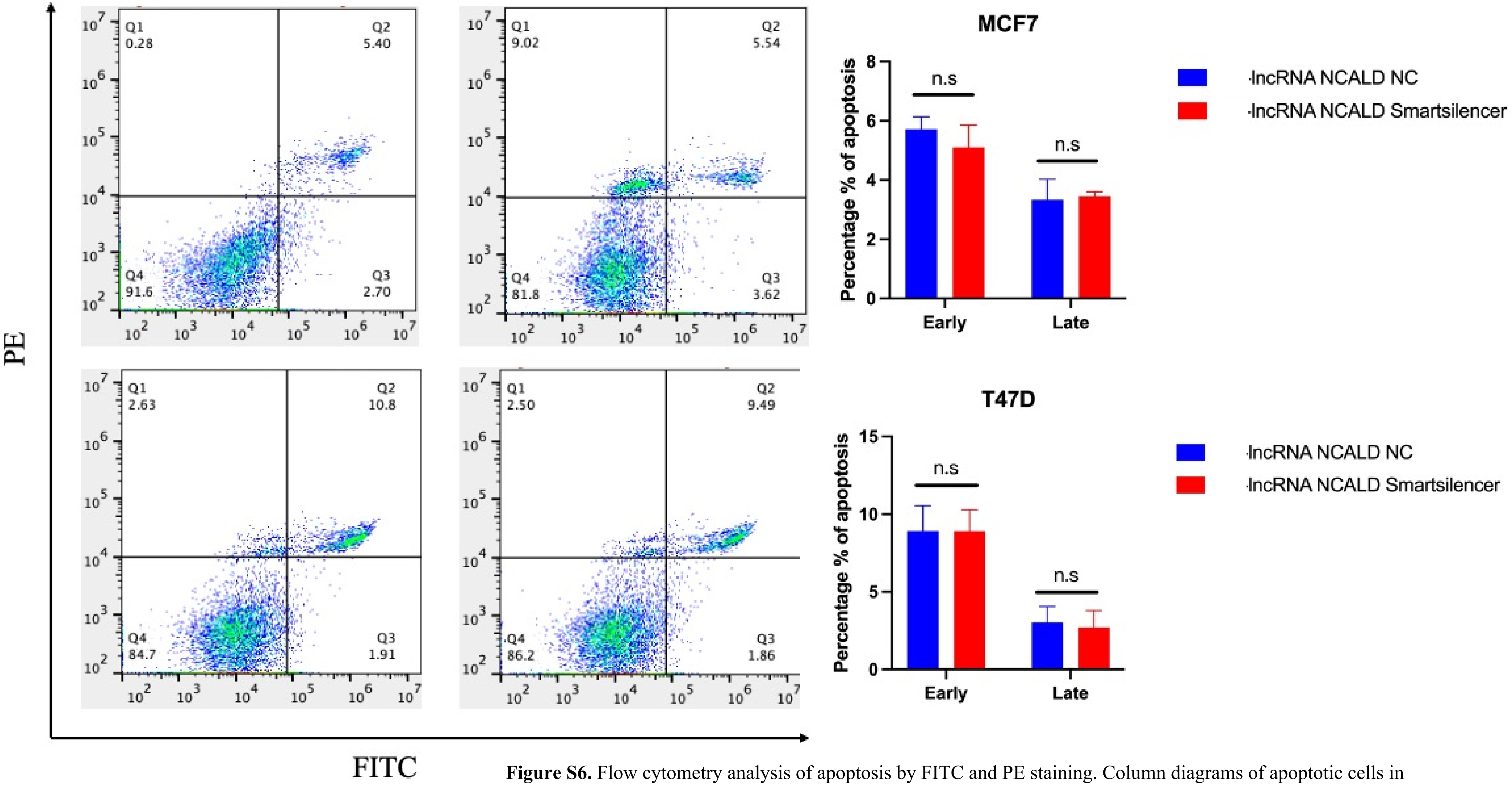

Figure S6. Flow cytometry analysis of apoptosis by FITC and PE staining. Column diagrams of apoptotic cells in percentage (n.s means no significant difference). All experiments were biologically replicated three times.

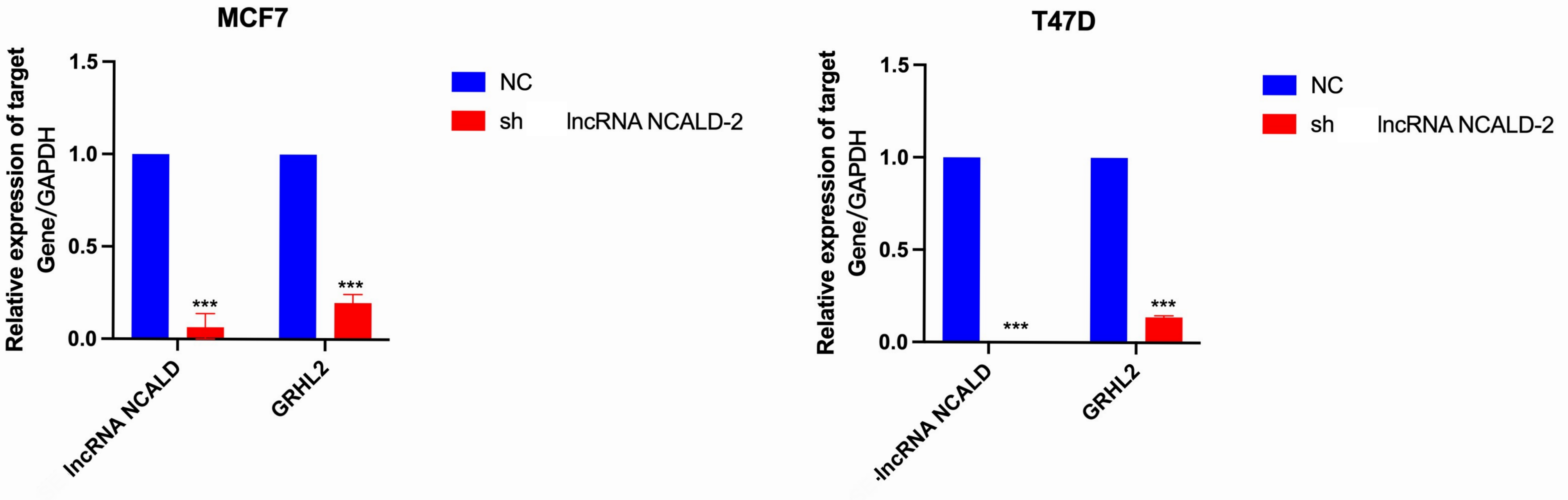

Figure S7. Knockdown of lncRNA NCALD caused by sh-lncRNA NCALD-2 plasmid inhibited GRHL2 expression in RNA level, ($n = 3$, mean ± SD; ***$P < 0.001$).

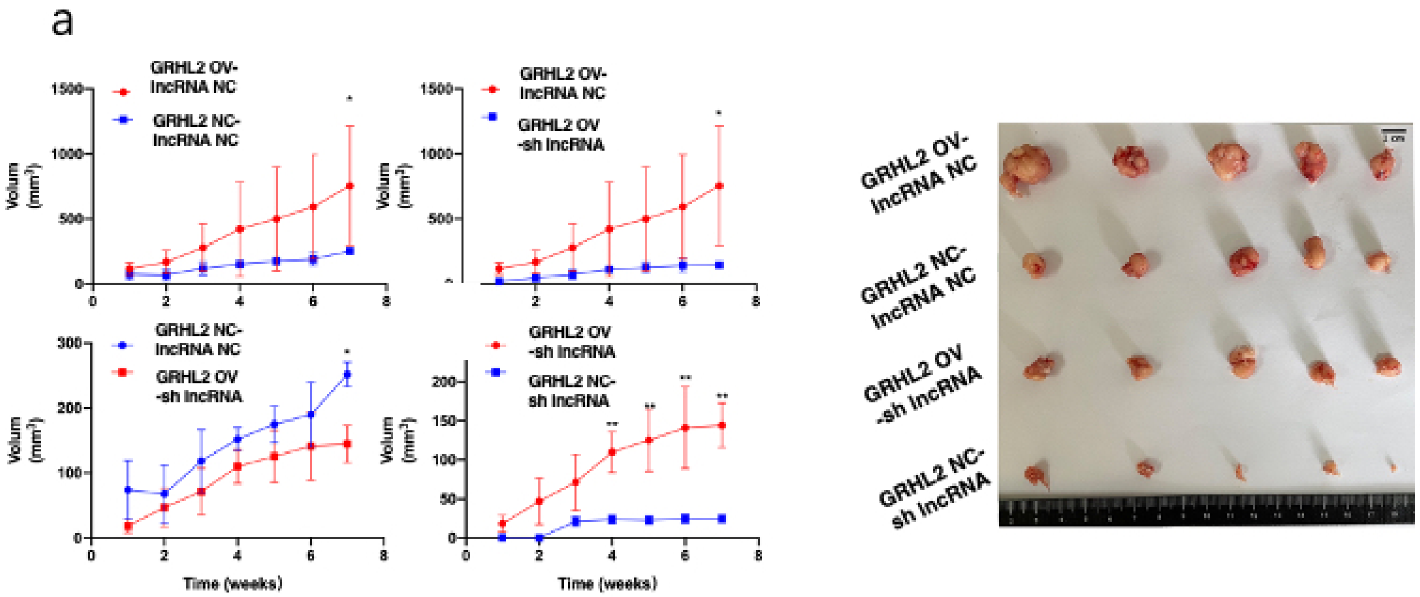

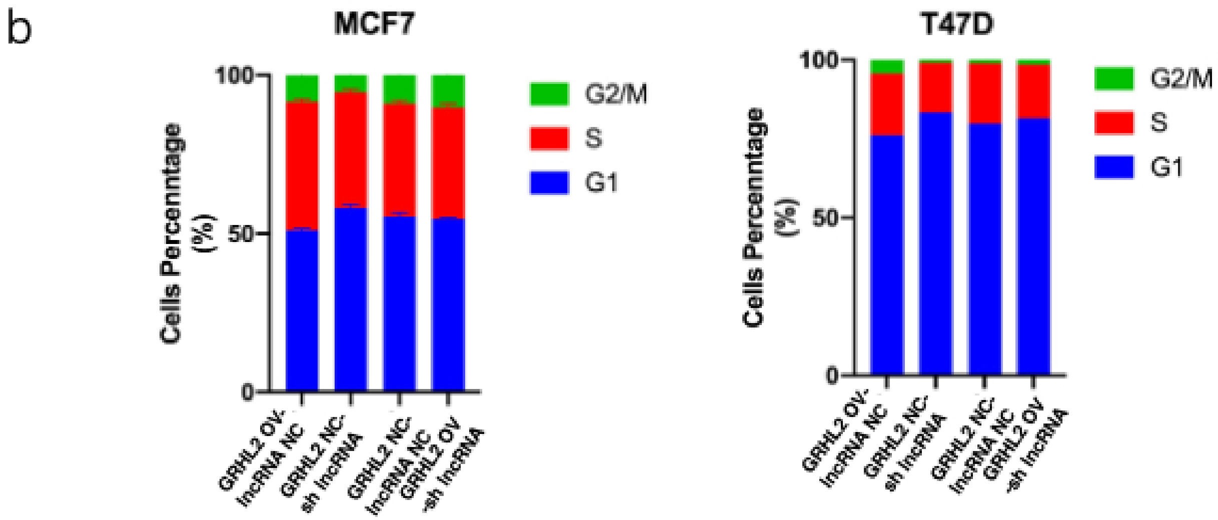

Figure S8. a The significant restoration of GRHL2 effectively reversed the suppressed tumor growth caused by the knockdown of lncRNA NCALD . The data points of the tumor growth curve are presented as the mean ± SD (n = 5/group) (*$P < 0.05$, **$P < 0.01$). b.Knockdown of lncRNA NCALD arrested cell in G1 cycle, which can be reversed by the overexpression of GRHL2 (n = 3, mean ± SD).

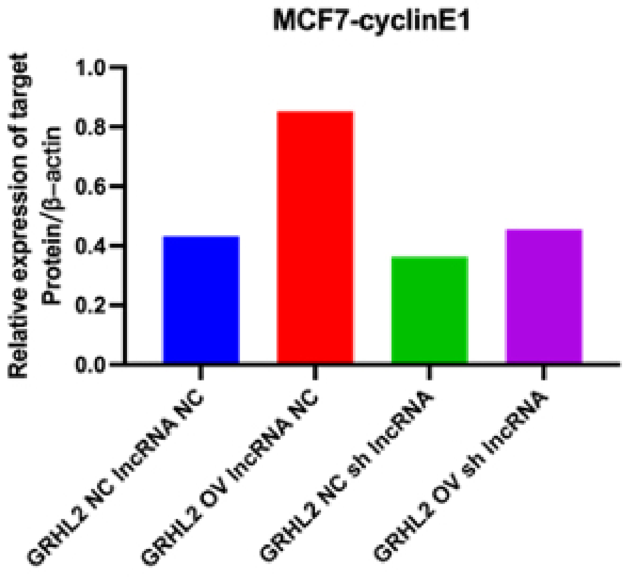
MCF7-cyclinE1
Relative expression of target Protein/β−actin
1.0
0.8
0.6
0.4
0.2
0.0
GRHL2 NC lncRNA NC
GRHL2 OV lncRNA NC
GRHL2 NC sh lncRNA
GRHL2 OV sh lncRNA

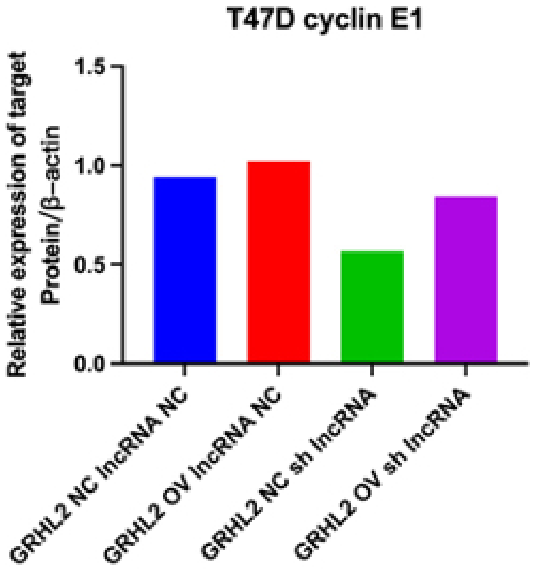
T47D cyclin E1
Relative expression of target Protein/β−actin
1.5
1.0
0.5
0.0
GRHL2 NC lncRNA NC
GRHL2 OV lncRNA NC
GRHL2 NC sh lncRNA
GRHL2 OV sh lncRNA
