# Supplementary material for: An estrogen-regulated long non-coding RNA NCALD promotes luminal breast cancer proliferation by activating GRHL2
Source: Cancer Cell Int. 2024 Jan 30;24:49. doi: 10.1186/s12935-024-03245-0 (PMC10829383; doi:10.1186/s12935-024-03245-0)
Supplement: Supplementary file 2 — Additional file 2. Raw data of microarray [file 12935_2024_3245_MOESM2_ESM.pdf]

Raw data of microarray

ProbeName	Fold Chang	Regulation	type	TransID	transcript_typ	GeneID	GeneSymbol	lncRNA_level	RNAlength	locus	overlapped_superenhancer_information
ASHG19SELNC2A100011138	2.6038	down	noncoding	ENST0000045161 8	noncoding	ENSG00000224413	AP001476.2	Gold	2198	chr21:47456966-47459721:+	chr21:47368269:47414613:Aorta;chr21:47368978:47399878:Fetal Muscle;chr21:47370640:47418875:Stomach Smooth Muscle;chr21:47373022:47488156:Ovary;chr21:47373188:47425562:NHDF-Ad;chr21:47373962:47413579:Lung;chr21:47390790:47421807:Esophagus;chr21:47390883:47405694:Bladder;chr21:47390890:47405005:NHLF;chr21:47441142:47554313:NHDF-Ad;chr21:47441234:47488057:Sigmoid Colon;chr21:47441247:47488321:Fetal Muscle;chr21:47441257:47531436:Esophagus;chr21:47444324:47564240:Osteoblasts;chr21:47444870:47513373:Astrocytes;chr21:47447910:47520896:NHLF;chr21:47457705:47513717:u87;chr21:47458019:47488324:Aorta;chr21:47468502:47488351:Stomach Smooth Muscle;chr21:47468608:47520268:IMR90;chr21:475
ASHG19SELNC2A100007759	3.0444	up	noncoding	uc002xyu.1	noncoding	AK056098	AK056098		2570	chr20:56285239-56287808:+	chr20:56229534:56258510:Jurkat;chr20:56246672:56287104:CD20;chr20:56251067:56284317:Brain Anterior Caudate;chr20:56252467:56259279:MM1S;chr20:56253372:56286564:Ly4;chr20:56255682:56294468:Aorta;chr20:56257900:56300726:Adipose Nuclei;chr20:56261450:56303879:HCC1954;chr20:56261686:56292224:HMEC;chr20:56261714:56293621:NHEK;chr20:56261966:56294255:VACO 503;chr20:56262534:56294254:VACO 9m

ASHG19SELNC2A100016582	3.4515	down	noncoding	ENST000004507 46	noncoding	ENSG00000227260	AC116035.1	Gold	436	chr3:30566237-30568677:+	chr3:30469138:30555788:HSMMtube;chr3:30496390:30591982:Adipose Nuclei;chr3:30511426:30554266:HSMM;chr3:30511944:30554348:Skeletal Muscle Myoblast;chr3:30533581:30560947:HMEC;chr3:30551551:30592247:HUVEC	

ASHG19SELNC2A100017010	2.3424	down	noncoding	ENST000006029 77	noncoding	ENSG00000270001	RP11-218C14.8	Reliable	491	chr20:23612463-23612953:-	chr20:23609017:23622743:HepG2

ASHG19SELNC2A100016678	2.1005	down	noncoding	ENST0000432251	noncoding	ENSG00000233397	AC008063.3	Gold	619	chr2:162949939-162951364:+	chr2:162912779:162950883:Fetal Intestine Large;chr2:162945520:162950998:NHEK

ASHG19SELNC2A100133219	2.696	down	noncoding	ENST00000453517	noncoding	ENSG00000224063	AC007319.1	802	chr2:187867947-188392007:+	chr2:188267043:188314011:HUVEC

ASHG19SELNC2A100133219	2.696	down	noncoding	ENST0000045351 7	noncoding	ENSG00000224063	AC007319.1	802	chr2:187867947-188392007:+	chr2:188384010:188415175:HCC1954;chr2:188389384:188419998:Osteoblasts;chr2:188389431:188420188:u87;chr2:188389786:188418294:Panc1;chr2:188405332:188420155:HUVEC;chr2:188405621:188419612:HSMM;chr2:188405635:188419527:Skeletal Muscle Myoblast;chr2:188411722:188420090:CD34 Primary RO01536

ASHG19SELNC2A100018404	3.416	up	noncoding	ENST000005604 87	noncoding	ENSG00000259711	CTD-3032H12.2	359	chr16:54968825-54988577:+	chr16:54954572:54971158:u87;chr16:54956669:54972978:Left Ventricle;chr16:54956715:54973307:Lung;chr16:54959639:54972666:HMEC;chr16:54961499:54972180:Gastric

ASHG19SELNC2A100509795	6.2257	down	noncoding	ENST00000544890	noncoding	ENSG00000235423	RP11-282O18.3		796	chr12:123743524-123745766:-	chr12:123707408:123763358:CD34 Primary RO01536;chr12:123707459:123728805:Fetal Thymus
ASHG19SELNC2A100010845	2.2008	down	noncoding	MICT00000096712	noncoding	CATG00000017584	CATG00000017584.1		2380	chr13:80615818-80629005:-	chr13:80593904:80631065:u87
ASHG19SELNC2A100010672	7.0859	up	noncoding	ENST00000522494	noncoding	ENSG00000254205	RP11-92K15.1	Reliable	364	chr8:81035087-81039905:+	chr8:80927615:81085156:CD20;chr8:81033109:81081429:Brain Cingulate Gyrus;chr8:81033331:81084576:Tonsil;chr8:81033340:81084616:CD19 Primary;chr8:81033954:81085230:Ly1;chr8:81045809:81084636:GM12878;chr8:81048638:81085059:DHL6;chr8:81048663:81087135:Toledo;chr8:81048681:81085173:Ly4;chr8:81048776:81084522:MM1S

ASHG19SELNC2A100004675	2.6437	up	noncoding	ENST00000592982	noncoding	ENSG00000267296	CEBPA-AS1	2198	chr19:33793763-33795960:+	chr19:33731236:33808732:Colon Crypt 3;chr19:33738816:33795450:CD14;chr19:33751162:33802757:Lung;chr19:33762587:33809528:Colon Crypt 1;chr19:33763913:33809521:VACO 400;chr19:33764237:33808704:Sigmoid Colon;chr19:33764326:33809516:Colon Crypt 2;chr19:33764404:33802495:Esophagus;chr19:33764408:33808979:Fetal Intestine;chr19:33764433:33802794:Gastric;chr19:33764450:33808989:Fetal Intestine Large;chr19:33764588:33796341:HepG2;chr19:33781201:33808848:Small Intestine;chr19:33782013:33802787:LNCaP

ASHG19SELNC2A100003993	9.1453	up	noncoding	ENST00000605281	noncoding	ENSG00000271367	RP3-483K16.4	Reliable	548	chr6:53214956-53215503:+	chr6:53036329:53227410:CD20;chr6:53036599:53116245:CD19 Primary;chr6:53100514:53226188:Adipose Nuclei;chr6:53128178:53225333:CD14;chr6:53143027:53229608:Jurkat;chr6:53146409:53227384:CD4p CD25- Il17- PMAstim Th;chr6:53146723:53227390:CD4p CD25- Il17p PMAstim Th17;chr6:53154779:53227532:Fetal Thymus;chr6:53154998:53228030:DND41;chr6:53158989:53180047:RPMI-8402;chr6:53163931:53226897:Panc1;chr6:53164749:53181309:HUVEC;chr6:53165560:53203522:Thymus;chr6:53166397:53227225:CD34 Primary RO01536;chr6:53190554:53225640:CD19 Primary;chr6:53190767:53225498:Ly4;chr6:53196085:53227890:CD4p CD25- CD45ROp Memory;chr6:53196226:53227225:CD4 Memory Primary 7pool;chr6:53196256:53227329:CD8 primiary;chr6:53196355:53227514:CD4p CD25- CD45RAp Naive;chr6:53196563:53227334:CD4p CD225int CD127p Tmem;chr6:53196686:53227248:CD3;chr6:53196787:53227022:Tonsil;chr6:53197275:53227398:CD56;chr6:53197489:53227236:CD4 Memory Primary 8pool;chr6:53197881:53224849:CD4 Naive Primary 7pool;chr6:53198177:53226968:CD4 Naive Primary 8pool;chr6:53199221:53226955:CD8 Naive 7pool;chr6:53199226:53226968:CD8 Memory 7pool;chr6:53199602:53225903:Fetal Muscle

ASHG19SELNC2A101070378	3.745	down	noncoding	ENST0000536835	noncoding	ENSG00000256802	RP11-680F8.1	Reliable	2530	chr15:29967194-29971372:+	chr15:29962239:29981983:Colon Crypt 1;chr15:29962672:29981651:Colon Crypt 2
ASHG19SELNC2A100013142	3.4703	up	noncoding	ENST0000608206	noncoding	ENSG00000272989	RP13-616I3.1	Reliable	1725	chr3:197232133-197233857:-	chr3:197182362:197247617:Osteoblasts;chr3:197182483:197251089:Pancreatic islets;chr3:197182511:197222729:HSMMtube;chr3:197182681:197248452:u87;chr3:197182694:197219959:HSMM;chr3:197182708:197219870:Skeletal Muscle Myoblast;chr3:197191173:197199703:HepG2;chr3:197205399:197248702:VACO 9m;chr3:197208959:197283348:Esophagus;chr3:197219947:197251007:Colon Crypt 1;chr3:197219992:197250392:Colon Crypt 2;chr3:197220594:197250278:Small Intestine;chr3:197238701:197250196:Fetal Intestine;chr3:197238747:197250317:Fetal Intestine Large;chr3:197274001:197287045:Colon Crypt 3

ASHG19SELNC2A100981549	6.247	up	noncoding	ENST00000561529	noncoding	ENSG00000260886	RP11-432I5.1	873	chr16:71599692-71612090:+	chr16:71605152:71608364:HepG2

ASHG19SELNC2A100839170	5.3554	down	noncoding	ENST000005021 25	noncoding	ENSG00000247809	NR2F2-AS1	1930	chr15:96812001-96870590:-	chr15:96811654:96841238:Astrocytes

ASHG19SELNC2A100839170	5.3554	down	noncoding	ENST0000050212 5	noncoding	ENSG00000247809	NR2F2-AS1	1930	chr15:96812001-96870590:-	chr15:96843794:96900436:Fetal Muscle;chr15:96843842:96906957:Spleen;chr15:96845619:96910635:Stomach Smooth Muscle;chr15:96854958:96904044:Ovary;chr15:96859803:96901322:Duodenum Smooth Muscle;chr15:96860092:96903991:HUVEC;chr15:96860236:96900756:Right Atrium;chr15:96860251:96903800:Lung;chr15:96860327:96939510:Gastric;chr15:96863690:96903716:Astrocytes;chr15:96863797:96905060:Esophagus;chr15:96863802:96904281:Left Ventricle;chr15:96863879:96904071:Brain Hippocampus Middle;chr15:96863927:96903646:Adrenal Gland;chr15:96863929:96900992:Skeletal Muscle;chr15:96864068:96901557:Panc1;chr15:96864146:96912049:Pancreatic islets;chr15:96864155:96891159:Psoas Muscle;chr15:96864262:96901557:Brain Hippocampus Middle 150;chr15:96864441:96903811:Sigmoid Colon;chr15:96864462:96898142:Aorta;chr15:96864499:96901249:Osteoblasts;chr15:96864502:96898263:Small Intestine;chr15:96864556:96903688:NHLF;chr15:96864606:96898944:NHDF-Ad;chr15:96864624:96898150:Bladder;chr15:96867016:96901350:Adipose Nuclei;chr15:96867102:96898961:IMR90;chr15:96867427:96884039:MCF-

ASHG19SELNC2A101017201	3.519	down	noncoding	ENST00000432370	noncoding	ENSG00000237986	CELF2-AS2		853	chr10:11117094-11140436:-	chr10:11034219:11079146:HSMMtube;chr10:11049192:11078288:HSMM;chr10:11049251:11078274:Skeletal Muscle Myoblast;chr10:11051880:11069686:Fetal Muscle;chr10:11054424:11131833:Left Ventricle
ASHG19SELNC2A100198090	2.8499	up	noncoding	ENST00000526487	noncoding	ENSG00000255129	RP11-839D17.3		588	chr11:113149275-113185146:-	chr11:113102167:113146901:Brain Inferior Temporal Lobe;chr11:113102330:113150528:Brain Anterior Caudate;chr11:113103771:113146672:Brain Hippocampus Middle;chr11:113104958:113150986:Brain Cingulate Gyrus;chr11:113110834:113160849:HSMMtube
ASHG19SELNC2A100006978	5.0289	up	noncoding	NR_131157	noncoding	NKILA	NKILA	Gold	2615	chr20:56285239-56287836:+	chr20:56229534:56258510:Jurkat;chr20:56246672:56287104:CD20;chr20:56251067:56284317:Brain Anterior Caudate;chr20:56252467:56259279:MM1S;chr20:56253372:56286564:Ly4;chr20:56255682:56294468:Aorta;chr20:56257900:56300726:Adipose Nuclei;chr20:56261450:56303879:HCC1954;chr20:56261686:56292224:HMEC;chr20:56261714:56293621:NHEK;chr20:56261966:56294255:VACO 503;chr20:56262534:56294254:VACO 9m

ASHG19SELNC2A100006209	11.48	up	noncoding	ENST000006070 17	noncoding	ENSG00000272264	RP11-92K15.3	Reliable	577	chr8:80944959-80945535:-	chr8:80927615:81085156:CD20;chr8:81033109:81081429:Brain Cingulate Gyrus;chr8:81033331:81084576:Tonsil;chr8:81033340:81084616:CD19 Primary;chr8:81033954:81085230:Ly1;chr8:81045809:81084636:GM12878;chr8:81048638:81085059:DHL6;chr8:81048663:81087135:Toledo;chr8:81048681:81085173:Ly4;chr8:81048776:81084522:MM1S

ASHG19SELNC2A100008357	2.1313	down	noncoding	ENST00000526269	noncoding	ENSG00000254703	SENCR		1298	chr11:128561567-128565918:-	chr11:128539687:128591438:RPMI-8402;chr11:128546321:128643284:CD34 Primary RO01536;chr11:128547574:128600678:HUVEC;chr11:128547730:128674908:Spleen;chr11:128555577:128610042:Ly1;chr11:128555988:128567972:Tonsil;chr11:128560125:128616737:Lung;chr11:128560330:128599656:Adipose Nuclei;chr11:128561748:128599291:Right Atrium;chr11:128561882:128591019:Fetal Muscle;chr11:128562324:128604385:Osteoblasts;chr11:128562350:128654166:CD14;chr11:128562395:128679347:CD20;chr11:128562503:128605194:Small Intestine;chr11:128562685:128628864:CD8 primiary;chr11:128562700:128628023:CD56;chr11:128562767:128628950:Ly4;chr11:128562805:128677186:CD4p CD25- Il17- PMAstim Th;chr11:128563170:128609883:DND41;chr11:128564879:128611094:Jurkat;chr11:128582503:128610944:CD19 Primary;chr11:128582760:128618325:CD3;chr11:128582794:128629104:Tonsil;chr11:128585066:128609559:DHL6;chr11:128585163:128630250:MM1S;chr11:128585301:128610080:Sigmoid Colon;chr11:128585928:128609212:Thymus

ASHG19 SELNC2 A100515 178	8.6375	up	noncoding	ENST00 0005307 78	noncodi ng	ENSG000002 45149	RNF139-AS1	Gold	1767	chr8:12548 5051- 125486817: -	chr8:125483998:125489483:CD4 Memory Primary 7pool;chr8:125484090:125489608:CD8 Memory 7pool;chr8:125485153:125489587:CD4 Memory Primary 8pool;chr8:125485354:125489304:CD4 Naive Primary 8pool;chr8:125485368:125489205:CD8 Naive 7pool;chr8:125485632:125489329:CD4 Naive Primary 7pool;chr8:125485702:125489235:Spleen

ASHG19SELNC2A100008954	3.1131	down	noncoding	ENST00000440492	noncoding	ENSG00000233975	RP11-288L9.1	441	chr1:27986839-27989233:-	chr1:27715023:27763264:Right Atrium;chr1:27717086:27764296:Brain Hippocampus Middle;chr1:27721859:27765916:Left Ventricle;chr1:27724397:27820500:CD20;chr1:27725337:27778490:Fetal Muscle;chr1:27727708:27765915:Adipose Nuclei;chr1:27727756:27763633:Esophagus;chr1:27727767:27766345:Toledo;chr1:27728493:27766344:HSMMtube;chr1:27728639:27778340:Brain Anterior Caudate;chr1:27728653:27765964:Brain Cingulate Gyrus;chr1:27728715:27819731:CD14;chr1:27728718:27764261:Brain Inferior Temporal Lobe;chr1:27728799:27782366:Duodenum Smooth Muscle;chr1:27728807:27763107:Thymus;chr1:27728880:27762357:CD34 Primary RO01536;chr1:27728950:27761500:Brain Angular Gyrus;chr1:27743024:27792274:MM1S;chr1:27799873:27826360:DHL6;chr1:27801410:27856116:Tonsil;chr1:27808338:27869987:Brain Cingulate Gyrus;chr1:27814191:27931064:Fetal Intestine Large;chr1:27814202:27903953:Brain Hippocampus Middle 150;chr1:27814251:27929612:Fetal Intestine;chr1:27814257:27905229:HSMMtube;chr1:27814303:27902896:Colon Crypt 3;chr1:27814306:27934479:Fetal Muscle;chr1:27814309:27904188:Duodenum Smooth Muscle;chr1:27814337:27930846:Brain Inferior Temporal Lobe;chr1:27814348:27933300:Brain Hippocampus Middle;chr1:27814375:27895242:HeLa;chr1:2781439

ASHG19SELNC2A100008835	2.9063	up	noncoding	ENST00000442226	noncoding	ENSG00000225986	UBXN10-AS1	510	chr1:20510735-20512979:-	chr1:20506105:20540518:HCC1954;chr1:20509945:20516325:K562

ASHG19SELNC2A100006588	2.0098	down	noncoding	ENST0000042645 3	noncoding	ENSG00000231441	RP11-472M19.2		551	chr6:56708800-56728876:+	chr6:56502602:56581913:Brain Cingulate Gyrus;chr6:56502838:56581696:Brain Anterior Caudate;chr6:56502845:56581837:Brain Hippocampus Middle 150;chr6:56502861:56535165:HMEC;chr6:56502894:56539089:Brain Inferior Temporal Lobe;chr6:56502995:56536202:NHEK;chr6:56513204:56560621:HCC1954;chr6:56513685:56559671:Fetal Intestine Large;chr6:56520482:56559710:Fetal Intestine;chr6:56524224:56578736:Skeletal Muscle;chr6:56524595:56638092:Left Ventricle;chr6:56534362:56638518:Osteoblasts;chr6:56571865:56623920:Panc1;chr6:56571905:56599574:u87;chr6:56594050:56626671:HMEC;chr6:56606682:56638124:Brain Cingulate Gyrus;chr6:56611492:56651397:Brain Anterior Caudate;chr6:56613641:56680435:HSMMtube;chr6:56654686:56711326:Skeletal Muscle;chr6:56660455:56679904:HSMM;chr6:56660469:56679904:Skeletal Muscle Myoblast;chr6:56667271:56717953:Brain Cingulate Gyrus;chr6:56702566:56735177:HSMMtube;chr6:56703330:56777597:u87;chr6:56703556:56735029:HSMM;chr6:56703775:56734987:Skeletal Muscle Myoblast

ASHG19SELNC2A100703474	3.5479	down	noncoding	ENST000005179 27	noncodi ng	ENSG00000253522	MIR146A	Reliable	2301	chr5:159895275-159914433:+	chr5:159865596:159905010:GM12878;chr5:159893483:159905754:HepG2

ASHG19SELNC2A100016780	2.0504	down	noncoding	ENST00000499966	noncoding	ENSG00000246898	LINC00920	2147	chr16:66442427-66446038:+	chr16:66390995:66420521:HUVEC;chr16:66392024:66418292:Fetal Muscle;chr16:66392035:66422229:Lung;chr16:66392306:66443386:Adipose Nuclei;chr16:66392592:66418106:Left Ventricle;chr16:66392705:66417964:Right Atrium

ASHG19 SELNC2 A100004 771	11.247	down	noncoding	FTMT2 1400001 584	noncodi ng	CATG000000 72156	CATG000000 72156.1	1132	chr4:26085 946- 26087077:-	chr4:25996538:26036432:Osteoblasts;chr4:26013996 :26052472:Fetal Muscle;chr4:26015538:26036372:NHDF- Ad;chr4:26050417:26092107:Ly3;chr4:26051599:26 096077:HBL1;chr4:26075171:26095996:CD4p CD25- Il17p PMAstim Th17

ASHG19SELNC2A100009974	2.4696	up	noncoding	ENST000005642 48	noncoding	ENSG00000260418	RP3-406A7.7	Reliable	374	chr6:136656852-136657225:-	chr6:136626675:136825292:Brain Hippocampus Middle 150;chr6:136645304:136854457:Brain Cingulate Gyrus;chr6:136645356:136705407:Brain Inferior Temporal Lobe;chr6:136722695:136768750:Brain Inferior Temporal Lobe;chr6:136722901:136761865:Brain Anterior Caudate;chr6:136722912:136768334:Brain Hippocampus Middle;chr6:136724053:136766443:Brain Angular Gyrus;chr6:136781412:136855111:Brain Inferior Temporal Lobe;chr6:136811569:136853648:Brain Anterior Caudate;chr6:136819007:136825461:H1;chr6:136832967:136853528:Brain Angular Gyrus;chr6:136837841:136854150:Brain Hippocampus Middle 150

ASHG19SELNC2A100012481	2.1958	down	noncoding	ENCT000003515 82	noncoding	CATG00000078038	CATG00000078038.1	2129	chr5:149539613-149541741:+	chr5:149503252:149541427:Spleen;chr5:149504602:149543232:Ovary;chr5:149507350:149562929:Aorta;chr5:149507482:149541625:Osteoblasts;chr5:149508783:149544885:Brain Hippocampus Middle;chr5:149509027:149542236:u87;chr5:149510774:149544849:Left Ventricle;chr5:149511246:149544603:Right Atrium;chr5:149513384:149536425:IMR90;chr5:149513886:149544807:Lung;chr5:149516216:149540715:Astrocytes;chr5:149516288:149536416:HSMMtube;chr5:149516544:149566936:Small Intestine;chr5:149516880:149525103:NHLF;chr5:149516932:149565271:Duodenum Smooth Muscle;chr5:149516984:149569007:Fetal Intestine;chr5:149517172:149541546:NHDF-Ad;chr5:149517354:149546152:Fetal Muscle;chr5:149517656:149533391:Brain Mid Frontal Lobe;chr5:149540335:149569333:Fetal Intestine Large;chr5:149540778:149566941:Colon Crypt 3;chr5:149540841:149566799:Colon Crypt 2;chr5:149540883:149566877:Colon Crypt 1;chr5:149554619:149566880:Sigmoid Colon

ASHG19SELNC2A100003764	2.2432	up	noncoding	ENST000004996 42	noncoding	ENSG00000246145	RP11-346I3.4	4366	chr8:67331824-67341212:-	chr8:67340038:67371615:Psoas Muscle;chr8:67340067:67367435:Skeletal Muscle;chr8:67340114:67385009:Left Ventricle;chr8:67340126:67368514:Lung

ASHG19SELNC2A100018734	2.2986	up	noncoding	ENST0000005226 74	noncoding	ENSG00000240990	HOXA11-AS	1549	chr7:27225027-27228912:+	chr7:27134391:27135361:NHEK;chr7:27134377:27144793:HMEC;chr7:27134611:27149405:HUVEC;chr7:27135447:27166356:Fetal Intestine;chr7:27135483:27172379:Stomach Smooth Muscle;chr7:27135550:27153505:Duodenum Smooth Muscle;chr7:27135686:27164694:Astrocytes;chr7:27135736:27164850:IMR90;chr7:27137333:27163753:Aorta;chr7:27139764:27158843:NHLF;chr7:27149969:27156855:HCC1954;chr7:27150046:27165993:HUVEC;chr7:27152313:27161149:Esophagus;chr7:27153676:27170089:Duodenum Smooth Muscle;chr7:27153682:27163924:Panc1;chr7:27153688:27196950:Adipose Nuclei;chr7:27168979:27220885:HUVEC;chr7:27170197:27183467:Duodenum Smooth Muscle;chr7:27175056:27185979:HMEC;chr7:27175128:27193124:NHLF;chr7:27178045:27192976:IMR90;chr7:27178057:27189568:Sigmoid Colon;chr7:27183589:27193724:Fetal Muscle;chr7:27187599:27213613:CD34 Primary RO01536;chr7:27189595:27202231:Sigmoid Colon;chr7:27189615:27192995:VACO 400;chr7:27193620:27202197:VACO 400;chr7:27194720:27202203:VACO 503;chr7:27196844:27213703:CD34 Primary RO01549;chr7:27198105:27233826:Fetal Muscle;chr7:27199735:27220891:Adipose Nuclei;chr7:27201314:27230078:Osteoblasts;chr7:27202582:27214744:HCT-116;chr7:27202624:27220717:NHDF-

ASHG19SELNC2A100017817	2.0847	down	noncoding	ENCT000003230 70	noncodi ng	CATG00000069858	CATG00000069858.1	1407	chr4:115199105-115200511:+	chr4:115178056:115208536:Osteoblasts

ASHG19SELNC2A101117053	2.5919	down	noncoding	ENST000005546 87	noncoding	ENSG00000259048	CTD-2058B24.2	Reliable	785	chr14:38503492-38663486:-	chr14:38532475:38559437:CD56;chr14:38532657:38559557:CD3

ASHG19SELNC2A101117053	2.5919	down	noncoding	ENST000005546 87	noncoding	ENSG00000259048	CTD-2058B24.2	Reliable	785	chr14:38503492-38663486:-	chr14:38656566:38667401:H1

ASHG19SELNC2A100002932	2.3355	down	noncoding	ENST00000499583	noncoding	ENSG00000245812	RP11-175K6.1		2664	chr5:158527690-158544486:+	chr5:158117077:158371526:Adipose Nuclei;chr5:158121898:158135119:u87;chr5:158121972:158131638:Osteoblasts;chr5:158150800:158180825:Fetal Muscle;chr5:158168868:158186971:Toledo;chr5:158169736:158256293:CD20;chr5:158171269:158211571:Ly1;chr5:158173326:158188865:Osteoblasts;chr5:158271663:158346383:CD20;chr5:158272434:158413938:Ly1;chr5:158300843:158414355:HBL1;chr5:158359507:158533725:CD20;chr5:158369073:158501584:Osteoblasts;chr5:158379947:158501405:Fetal Muscle;chr5:158422759:158490152:u87;chr5:158429879:158499195:Ly1;chr5:158433416:158528623:Ly4;chr5:158457028:158534580:Adipose Nuclei;chr5:158458184:158484073:HBL1;chr5:158509484:158534427:HBL1;chr5:158510435:158534323:Tonsil;chr5:158514566:158533975:Fetal Muscle;chr5:158514747:158534215:Ly1;chr5:158520124:158534388:Toledo

ASHG19SELNC2A100017348	4.1453	up	noncoding	ENST000004343 01	noncoding	ENSG00000230393	AC092667.2	Reliable	2586	chr2:100721381-100723966:+	chr2:100721400:100762028:CD20;chr2:100746570:100768229:Ly3;chr2:100746723:100768009:HBL1

ASHG19SELNC2A100015253	3.2167	up	noncoding	ENST000005202 68	noncodi ng	ENSG00000254024	KB-1562D12.1	Reliable	491	chr8:102473405-102504727:-	chr8:102435163:102487838:HCC1954;chr8:102446129:102530125:MCF-7;chr8:102448598:102489535:Panc1;chr8:102500357:102518753:HCC1954;chr8:102502873:102521092:HMEC;chr8:102503161:102517610:NHEK;chr8:102503445:102527174:Colon Crypt 3;chr8:102503837:102506939:VACO 9m

ASHG19SELNC2A101633320	3.7163	down	noncoding	ENST00000398275	noncoding	ENSG00000214401	KANSL1-AS1	517	chr17:44270942-44274087:+	chr17:44044333:44121889:Brain Hippocampus Middle;chr17:44096782:44274444:CD4p CD25- Il17- PMAstim Th;chr17:44164675:44273060:CD14;chr17:44215263:44273404:CD20;chr17:44215536:44273507:CD34 Primary RO01536;chr17:44230984:44273775:CD4p CD25- Il17p PMAstim Th17;chr17:44248846:44275782:CD4p CD25- CD45RAp Naive;chr17:44255747:44273300:DHL6;chr17:44256258:44272635:Spleen;chr17:44256345:44273776:Tonsil;chr17:44266427:44273061:CD34 Primary RO01549;chr17:44266858:44272835:Brain Hippocampus Middle 150;chr17:44266915:44274107:CD4 Memory Primary 7pool;chr17:44266931:44272683:NHEK;chr17:44267055:44273912:CD4 Naive Primary 8pool;chr17:44267124:44273411:CD8 Memory 7pool;chr17:44267165:44274318:CD8 Naive 8pool;chr17:44267190:44275611:Fetal Thymus;chr17:44267245:44275852:Jurkat;chr17:44267628:44273292:CD8 Naive 7pool;chr17:44267640:44273898:CD4 Memory Primary 8pool;chr17:44268170:44272891:CD34 Primary RO01480;chr17:44268206:44274185:CD4 Naive Primary 7pool;chr17:44268304:44273419:CD4p CD225int CD127p Tmem

ASHG19SELNC2A100003619	3.0351	up	noncoding	ENST00000415479	noncoding	ENSG00000230838	AC093850.2	Reliable	648	chr2:216582766-216584147:+	chr2:216516265:216662199:Osteoblasts;chr2:216520885:216674187:u87;chr2:216545306:216578353:Astrocytes;chr2:216556049:216585085:HUVEC

ASHG19SELNC2A100007335	2.7573	down	noncoding	ENST00000442176	noncoding	ENSG00000229108	AC005550.4	697	chr7:15728003-15735116:+	chr7:15687862:15732963:Fetal Muscle

ASHG19SELNC2A100525925	5.6421	down	noncoding	ENCT0000134095	noncoding	CATG00000021187	CATG00000021187.1		8252	chr14:55545548-55565160:-	chr14:55517374:55542334:CD20;chr14:55517498:55520386:NHLF;chr14:55537310:55545298:NHEK;chr14:55537362:55546494:HMEC;chr14:55538409:55599024:Esophagus;chr14:55543226:55609720:Fetal Intestine Large;chr14:55543381:55609338:Fetal Intestine;chr14:55543633:55599267:Colon Crypt 3;chr14:55555849:55606206:Osteoblasts;chr14:55555850:55590996:u87;chr14:55556020:55594550:H2171;chr14:55557509:55598917:Colon Crypt 2;chr14:55558308:55599128:Sigmoid Colon;chr14:55558414:55601665:Colon Crypt 1;chr14:55558583:55576148:NHDF-Ad;chr14:55560547:55599401:HeLa;chr14:55563236:55614101:Adipose Nuclei;chr14:55563567:55600547:Right Atrium;chr14:55563760:55599095:Lung;chr14:55563819:55606443:Gastric;chr14:55563949:55599030:Small Intestine;chr14:55563967:55614638:CD14;chr14:55564001:55599376:Duodenum Smooth Muscle;chr14:55564346:55595404:HCC1954;chr14:55564586:55601252:Left Ventricle;chr14:55568071:55575974:HSMMtube;chr14:55568080:55575933:NHLF;chr14:55568260:55575863:HMEC;chr14:55568330:55575714:NHEK;chr14:55568402:55598945:VACO 503;chr14:55568405:55576160:Astrocytes;chr14:55568888:55600476:Ovary;chr14:55569710:55575102:Adipose Tissue

ASHG19SELNC2A100893214	2.7086	down	noncoding	ENCT00000134094	noncoding	CATG00000021187	CATG00000021187.1		8242	chr14:55545548-55565179:-	chr14:55517374:55542334:CD20;chr14:55517498:55520386:NHLF;chr14:55537310:55545298:NHEK;chr14:55537362:55546494:HMEC;chr14:55538409:55599024:Esophagus;chr14:55543226:55609720:Fetal Intestine Large;chr14:55543381:55609338:Fetal Intestine;chr14:55543633:55599267:Colon Crypt 3;chr14:55555849:55606206:Osteoblasts;chr14:55555850:55590996:u87;chr14:55556020:55594550:H2171;chr14:55557509:55598917:Colon Crypt 2;chr14:55558308:55599128:Sigmoid Colon;chr14:55558414:55601665:Colon Crypt 1;chr14:55558583:55576148:NHDF-Ad;chr14:55560547:55599401:HeLa;chr14:55563236:55614101:Adipose Nuclei;chr14:55563567:55600547:Right Atrium;chr14:55563760:55599095:Lung;chr14:55563819:55606443:Gastric;chr14:55563949:55599030:Small Intestine;chr14:55563967:55614638:CD14;chr14:55564001:55599376:Duodenum Smooth Muscle;chr14:55564346:55595404:HCC1954;chr14:55564586:55601252:Left Ventricle;chr14:55568071:55575974:HSMMtube;chr14:55568080:55575933:NHLF;chr14:55568260:55575863:HMEC;chr14:55568330:55575714:NHEK;chr14:55568402:55598945:VACO 503;chr14:55568405:55576160:Astrocytes;chr14:55568888:55600476:Ovary;chr14:55569710:55575102:Adipose Tissue

ASHG19SELNC2A100243511	2.5674	down	noncoding	ENST000005198 80	noncoding	ENSG00000254286	RP11-89K10.1		574	chr8:127570120-127725660:+	chr8:127534539:127575687:Fetal Intestine Large;chr8:127535085:127575544:Fetal Intestine;chr8:127546423:127606030:HMEC;chr8:127548311:127593375:HCC1954;chr8:127567091:127577136:Gastric;chr8:127567492:127570739:VACO9m

ASHG19SELNC2A100243511	2.5674	down	noncoding	ENST000005198 80	noncoding	ENSG00000254286	RP11-89K10.1	574	chr8:127570120-127725660:+	chr8:127641238:127660919:Brain Hippocampus Middle 150
ASHG19SELNC2A100361851	2.323	down	noncoding	ENST0000050403 2	noncoding	ENSG00000249348	UGDH-AS1	2787	chr4:39529639-39596327:+	chr4:39569641:39642487:CD20;chr4:39609302:39642303:HBL1;chr4:39609871:39642539:Panc1

ASHG19SELNC2A100642515	6.8218	down	noncoding	ENST00000450715	noncoding	ENSG00000228505	AC011897.2	346	chr2:14756552-14770316:+	chr2:14760758:14777862:Fetal Intestine Large;chr2:14760941:14776971:Fetal Intestine

ASHG19SELNC2A100013917	2.4877	up	noncoding	ENST0000060 6067	noncoding	ENSG00000272155	RP11-707M3.3	445	chr8:66626569-66627013:-	chr8:66619768:66757228:CD4p CD25- Il17-PMAstim Th;chr8:66628792:66720654:Left Ventricle;chr8:66647994:66675128:DHL6;chr8:66652449:66767909:CD20;chr8:66692709:66756320:CD4p CD25- CD45RAp Naive;chr8:66728768:66756039:CD4p CD25- Il17p PMAstim Th17;chr8:66734112:66756365:CD8 primiary;chr8:66741431:66767174:Tonsil;chr8:66741939:66767197:CD19 Primary;chr8:66742426:66755991:CD4 Memory Primary 7pool;chr8:66748646:66755944:CD56;chr8:66748741:66755943:CD8 Naive 7pool;chr8:66749164:66755707:CD4 Naive Primary 7pool;chr8:66750712:66755519:CD34 Primary RO01480

ASHG19SELNC2A100010536	7.752	down	noncoding	ENST00000417638	noncoding	ENSG00000235448	RP11-3L8.3		799	chr9:12700100-12814344:-	chr9:12771064:12819477:HMEC;chr9:12772303:12820220:Adipose Nuclei;chr9:12774422:12827133:Duodenum Smooth Muscle;chr9:12774601:12818766:NHEK;chr9:12774741:12795687:HCC1954;chr9:12809247:12822241:Osteoblasts
ASHG19SELNC2A101026948	5.2257	up	noncoding	NR_103852	noncoding	ENSG00000260886	RP11-432I5.1	Gold	685	chr16:71598919-71606341:+	chr16:71605152:71608364:HepG2

ASHG19SELNC2A100007736	3.8703	up	noncoding	ENST000006086 84	noncoding	ENSG00000273338	RP11-386I14.4	Reliable	209	chr1:78470030-78470238:-	chr1:78412595:78447087:CD4p CD25- Il17-PMAstim Th;chr1:78440503:78446337:CD4p CD25-Il17p PMAstim Th17;chr1:78440827:78446362:CD8 primiary;chr1:78440854:78446748:CD4 Memory Primary 7pool;chr1:78440981:78446554:CD34 Primary RO01536;chr1:78440990:78472269:HMEC;chr1:78441202:78446868:CD8 Memory 7pool;chr1:78442114:78446661:HeLa;chr1:78442308:78446105:CD8 Naive 7pool;chr1:78468119:78474275:CD34 Primary RO01536;chr1:78468219:78474511:CD4 Memory Primary 7pool;chr1:78469587:78472772:CD34 Primary RO01480

ASHG19SELNC2A100094949	2.2444	up	noncoding	ENST00000415226	noncoding	ENSG00000233611	AC079135.1	Reliable	552	chr2:237076090-237203570:+	chr2:237074529:237089707:HSMM

ASHG19SELNC2A100094949	2.2444	up	noncoding	ENST00000415226	noncoding	ENSG00000233611	AC079135.1	Reliable	552	chr2:237076090-237203570:+	chr2:237144830:237174927:Left Ventricle

ASHG19SELNC2A100502053	3.0952	up	noncoding	ENST00000433595	noncoding	ENSG00000226272	ARHGAP26-AS1		978	chr5:142239169-142248475:-	chr5:142136972:142262349:CD14;chr5:142148681:142267010:Left Ventricle;chr5:142148743:142226830:Right Atrium;chr5:142148774:142205896:Duodenum Smooth Muscle;chr5:142162913:142226368:Sigmoid Colon;chr5:142173878:142209873:Fetal Intestine Large;chr5:142176144:142254943:Gastric;chr5:142177195:142264107:Lung;chr5:142181959:142249748:Fetal Muscle;chr5:142186071:142247539:Panc1;chr5:142186253:142215077:HepG2;chr5:142193268:142255285:Colon Crypt 1
ASHG19SELNC2A100010602	2.4179	up	noncoding	ENST00000499502	noncoding	ENSG00000246526	RP11-539L10.2	Reliable	1821	chr4:6689175-6692246:-	chr4:6675399:6698881:VACO 503;chr4:6689586:6699251:Gastric

ASHG19SELNC2A100000760	2.4793	up	noncoding	ENST000005992 74	noncoding	ENSG00000269246	CTC-246B18.10	Reliable	173	chr19:39832413-39832585:-	chr19:39794187:39822425:VACO 400;chr19:39810612:39864153:Tonsil;chr19:39825231:39866492:Spleen;chr19:39835067:39864460:Brain Anterior Caudate;chr19:39835251:39864330:Brain Angular Gyrus;chr19:39835544:39871845:Brain Hippocampus Middle 150;chr19:39844385:39858405:Brain Cingulate Gyrus;chr19:39845143:39867711:Brain Hippocampus Middle;chr19:39845385:39858315:Brain Inferior Temporal Lobe;chr19:39852821:39873874:Brain Mid Frontal Lobe;chr19:39864576:39910331:Panc1;chr19:39887353:39906237:CD20;chr19:39887382:39905568:CD4p CD25- CD45ROp Memory;chr19:39887413:39904333:CD14;chr19:39887465:39905704:Skeletal Muscle;chr19:39887512:39904080:HCT-116;chr19:39887524:39905043:HeLa;chr19:39887549:39905712:CD8 primiary;chr19:39887560:39906641:Brain Hippocampus Middle;chr19:39887567:39905352:Psoas Muscle;chr19:39887574:39925278:Esophagus;chr19:39887582:39905820:CD4p CD25- Il17- PMAstim Th;chr19:39887592:39902772:Colon Crypt 1;chr19:39887595:39905740:Small Intestine;chr19:39887598:39922292:Sigmoid Colon;chr19:39887615:39906154:Gastric;chr19:39887623:39905090:CD56;chr19:39887631:39906190:Ri

ASHG19SELNC2A100939874	2.0642	up	noncoding	MICT000002476 44	noncoding	CATG00000065539	CATG00000065539.1		6361	chr3:107684183-107706188:-	chr3:107687913:107726304:HCC1954
ASHG19SELNC2A100008481	2.0731	up	noncoding	ENST00000606921	noncoding	ENSG00000272402	RP1-30M3.6	Reliable	405	chr6:24706975-24707379:+	chr6:24689956:24723154:Adipose Nuclei;chr6:24699483:24722977:CD4p CD25- Il17- PMAstim Th;chr6:24704475:24722601:CD34 Primary RO01536;chr6:24716200:24722741:CD4 Memory Primary 7pool;chr6:24717070:24722878:CD8 primiary;chr6:24717126:24722883:Tonsil;chr6:24717401:24722848:CD8 Memory 7pool;chr6:24717427:24722705:CD4p CD25- Il17p PMAstim Th17;chr6:24717823:24723068:CD8 Naive 8pool;chr6:24717939:24722652:NHEK;chr6:24717975:24722681:CD4p CD225int CD127p Tmem;chr6:24717984:24722785:CD4 Memory Primary 8pool;chr6:24718102:24722633:CD4 Naive Primary 8pool;chr6:24718104:24722804:Fetal Muscle;chr6:24718107:24722890:CD8 Naive 7pool;chr6:24718189:24722854:CD56;chr6:24718403:24722902:Spleen;chr6:24718471:24722666:K562;chr6:24718524:24722844:CD3;chr6:24718676:24722761:CD4 Naive Primary 7pool;chr6:24719459:24722374:CD34 Primary RO01480;chr6:24719529:24722288:Colon Crypt 2

ASHG19SELNC2A100470650	2.3071	up	noncoding	ENST000005138 99	noncoding	ENSG00000249835	VCAN-AS1	453	chr5:82837296-82877139:-	chr5:82762834:82772725:Fetal Muscle;chr5:82766088:82838695:CD14;chr5:82766791:82772748:Astrocytes

ASHG19SELNC2A100002935	3.3541	up	noncoding	ENST000004890 11	noncoding	ENSG00000244468	RP11-206M11.7	Reliable	551	chr3:149002569-149051440:+	chr3:148988789:149126506:Panc1;chr3:149048300:149061677:HUVEC;chr3:149049808:149071618:u87;chr3:149056853:149120867:HeLa;chr3:149057580:149127143:Gastric;chr3:149073140:149120176:Fetal Intestine;chr3:149083029:149109672:HMEC;chr3:149083318:149107100:NHEK;chr3:149084101:149120838:HUVEC;chr3:149084137:149120740:Osteoblasts;chr3:149084285:149120708:HCC1954;chr3:149084395:149120202:Esophagus;chr3:149084790:149109422:Astrocytes;chr3:149087973:149107095:NHLF

ASHG19SELNC2A100006614	3.2328	up	noncoding	ENST00000421735	noncoding	ENSG00000232352	SEMA3B-AS1	338	chr3:50304073-50304803:-	chr3:50238336:50280738:Pancreas;chr3:50244838:50314281:Brain Hippocampus Middle 150;chr3:50245058:50313116:Brain Anterior Caudate;chr3:50247084:50313376:Brain Cingulate Gyrus;chr3:50247150:50313735:Brain Hippocampus Middle;chr3:50248043:50313227:Brain Inferior Temporal Lobe;chr3:50263296:50298495:CD4p CD25- CD45ROp Memory;chr3:50263305:50298785:Tonsil;chr3:50263386:50311338:HCT-116;chr3:50263388:50298592:CD4p CD25- CD45RAp Naive;chr3:50263790:50311735:Brain Mid Frontal Lobe;chr3:50263891:50292642:CD4p CD25- Il17- PMAstim Th;chr3:50263947:50299068:HUVEC;chr3:50264154:50298414:CD8 primiary;chr3:50264158:50296642:CD14;chr3:50264182:50298159:CD4p CD25- Il17p PMAstim Th17;chr3:50264192:50307591:Spleen;chr3:50264199:50309396:Lung;chr3:50264225:50309632:Small Intestine;chr3:50264235:50308232:Left Ventricle;chr3:50264239:50309560:Sigmoid Colon;chr3:50264241:50312025:Gastric;chr3:50264244:50307456:Psoas Muscle;chr3:50264249:50311529:Colon Crypt 3;chr3:50264266:50311141:Esophagus;chr3:50264267:50311261:Colon Crypt 1;chr3:50264277:50311114:Colon Crypt 2;chr3:50264284:50307169:NHLF;chr3:50264303:50308693:Aorta;chr3:50264311:50312803:Brain

ASHG19SELNC2A100003964	2.2787	up	noncoding	ENST00000591918	noncoding	ENSG00000266927	AC006273.7	Reliable	388	chr19:786650-787037:+	chr19:709079:728211:Ovary;chr19:727253:756891:Small Intestine;chr19:733218:756380:Colon Crypt 1;chr19:733267:757655:Colon Crypt 3;chr19:736895:756837:Fetal Intestine;chr19:736989:756634:Fetal Intestine Large;chr19:737545:755701:Colon Crypt 2;chr19:738309:757171:Sigmoid Colon;chr19:747547:756341:VACO 503;chr19:750343:797668:HSMMtube;chr19:772741:807650:Pancreatic islets;chr19:778437:802014:HeLa;chr19:778786:803779:Lung;chr19:778823:804693:Spleen;chr19:781851:802859:Adrenal Gland;chr19:781861:803389:Pancreas;chr19:788577:801770:K562;chr19:797352:807489:LNCaP
ASHG19SELNC2A100015321	2.0628	down	noncoding	ENST00000560714	noncoding	ENSG00000259607	CTD-2647L4.5	Reliable	585	chr8:28924796-28925971:+	chr8:28822411:28925363:CD20;chr8:28881051:28923940:CD19 Primary;chr8:28882012:28920814:CD8 primiary;chr8:28906874:28920394:CD4 Naive Primary 8pool;chr8:28910053:28934648:Brain Hippocampus Middle;chr8:28910933:28944469:HSMMtube;chr8:28911550:28937410:Brain Cingulate Gyrus;chr8:28911572:28937342:Brain Anterior Caudate
ASHG19SELNC2A100706943	3.2141	up	noncoding	ENST00000563101	noncoding	ENSG00000261360	CTD-2165H16.4	Reliable	2797	chr5:14661917-14664713:-	chr5:14659774:14688245:HMEC;chr5:14662875:14678963:Tonsil

ASHG19SELNC2A100000110	4.8905	down	noncoding	ENCT00000438909	noncoding	CATG00000103488	CATG00000103488.1		2127	chr8:104114413-104117614:-	chr8:104115978:104156783:u87;chr8:104143338:104159540:Brain Inferior Temporal Lobe
ASHG19SELNC2A100018955	9.1528	up	noncoding	ENST0000060699 3	noncoding	ENSG00000272141	RP11-465B22.8	Reliable	987	chr1:1104737-1105723:+	chr1:1058966:1101877:Small Intestine;chr1:1059412:1101686:Fetal Intestine Large;chr1:1059633:1101903:Fetal Intestine;chr1:1059682:1101913:VACO 400;chr1:1067578:1102178:LNCaP;chr1:1068186:1101896:Esophagus;chr1:1069414:1073756:Colon Crypt 3;chr1:1069443:1073717:Colon Crypt 2;chr1:1070790:1094387:CD19 Primary;chr1:1071352:1103985:VACO 9m;chr1:1077814:1082618:Colon Crypt 3;chr1:1077845:1081465:Colon Crypt 2;chr1:1079431:1101911:HCT-116;chr1:1090819:1107666:Gastric;chr1:1092804:1107737:Colon Crypt 3;chr1:1092807:1104591:Sigmoid Colon;chr1:1092822:1104598:Colon Crypt 1;chr1:1092838:1103486:Colon Crypt 2;chr1:1092849:1101754:Pancreas;chr1:1096780:1105079:Pancreatic islets;chr1:1097418:1104038:VACO 503

ASHG19SELNC2A100003564	2.0103	down	noncoding	ENST00000433475	noncoding	ENSG00000228108	AC092839.3	Gold	536	chr2:54743185-54767834:-	chr2:54586565:54904699:Adipose Nuclei;chr2:54586696:54663525:Fetal Muscle;chr2:54613104:54672107:Lung;chr2:54617571:54687691:Ly4;chr2:54636091:54712312:HSMMtube;chr2:54683758:54711159:Fetal Muscle;chr2:54685473:54869675:Lung;chr2:54745958:54901405:Brain Inferior Temporal Lobe;chr2:54749764:54818736:Toledo;chr2:54750088:54804603:HMEC;chr2:54750817:54816774:VACO 400;chr2:54751343:54804439:NHEK;chr2:54751452:54803224:VACO 503;chr2:54756686:54833095:Fetal Intestine Large;chr2:54758268:54832822:Fetal Intestine;chr2:54759619:54832571:Sigmoid Colon;chr2:54759741:54827867:Colon Crypt 3;chr2:54760045:54869686:Small Intestine;chr2:54760046:54846250:Colon Crypt 1;chr2:54761748:54831451:Colon Crypt 2;chr2:54772221:54842605:HUVEC;chr2:54772315:54847136:Duodenum Smooth Muscle;chr2:54772625:54869602:Gastric;chr2:54772694:54902160:Left Ventricle;chr2:54776502:54867867:Brain Anterior Caudate;chr2:54776871:54807844:MM1S;chr2:54777478:54832779:Fetal Thymus;chr2:54777855:54816759:Pancreas;chr2:54781785:54902053:Spleen;chr2:54782397:54814377:Osteoblasts;chr2:54782901:54866655:CD20;chr2:54783432:54831458:RPMI-8402;chr2:54783537:54830613:CD8

ASHG19SELNC2A100000793	2.3844	down	noncoding	ENST000006079 63	noncoding	ENSG00000273110	RP11-350G8.9	Reliable	681	chr1:154452488-154453977:+	chr1:154452194:154475601:HUVEC
ASHG19SUPERLNC2A100089200	3.0383	down	noncoding	ENST000005142 91	noncoding	ENSG00000244968	LIFR-AS1		685	chr5:38559044-38579696:+	

ASHG19SELNC2A100709371	2.0302	up	noncoding	ENST00000609514	noncoding	ENSG00000273143	RP11-525A16.4	488	chr10:112188598-112255945:-	chr10:112071042:112117651:u87;chr10:112100132:112156157:HBL1;chr10:112112550:112125490:CD4p CD25- Il17p PMAstim Th17;chr10:112112730:112119838:MM1S;chr10:112115390:112125208:VACO 9m;chr10:112135153:112176146:HUVEC;chr10:112146730:112185396:MM1S;chr10:112151756:112192739:HeLa;chr10:112152000:112171940:NHDF-Ad;chr10:112152059:112222295:Pancreatic islets;chr10:112152061:112187511:Osteoblasts;chr10:112152533:112189395:u87;chr10:112152627:112187468:NHLF;chr10:112153022:112177947:VACO 503;chr10:112153838:112221461:HCT-116;chr10:112154632:112176414:Fetal Intestine Large;chr10:112154864:112180852:HMEC;chr10:112155104:112175446:VACO 9m;chr10:112157290:112180905:NHEK;chr10:112166871:112184396:Ly4;chr10:112201172:112226248:MM1S
ASHG19SELNC2A100709371	2.0302	up	noncoding	ENST00000609514	noncoding	ENSG00000273143	RP11-525A16.4	488	chr10:112188598-112255945:-	chr10:112246051:112272983:HeLa;chr10:112246915:112297015:Osteoblasts;chr10:112247147:112290867:HUVEC;chr10:112247329:112307154:u87;chr10:112247411:112273365:Astrocytes;chr10:112252889:112273435:HMEC;chr10:112255287:112274294:NHDF-Ad;chr10:112255287:112274309:CD4p CD25- Il17- PMAstim Th;chr10:112255339:112274190:CD4p CD25- Il17p PMAstim Th17;chr10:112255360:112273473:NHLF;chr10:112255365:112296656:HCT-116;chr10:112255396:112273284:Lung;chr10:112255397:112273453:Left Ventricle;chr10:112255470:112261226:VACO 9m;chr10:112255651:112270145:Right Atrium

ASHG19SELNC2A100009680	2.0522	up	noncoding	ENST00000441214	noncoding	ENSG00000233077	RP11-290F20.2	2250	chr20:48927248-48937879:-	chr20:48701839:48789965:Duodenum Smooth Muscle;chr20:48702033:48738142:Ly4;chr20:48702124:48789661:Esophagus;chr20:48703584:48751057:Stomach Smooth Muscle;chr20:48713682:48790155:Skeletal Muscle;chr20:48715646:48789981:Psoas Muscle;chr20:48717052:48789891:Adipose Nuclei;chr20:48717422:48789836:Panc1;chr20:48718262:48789766:Small Intestine;chr20:48720232:48789657:Gastric;chr20:48724294:48730728:CD4p CD25- Il17p PMAstim Th17;chr20:48726272:48789783:Sigmoid Colon;chr20:48726275:48764260:Brain Inferior Temporal Lobe;chr20:48726309:48789939:Brain Hippocampus Middle;chr20:48726442:48767435:Brain Cingulate Gyrus;chr20:48726661:48783250:NHEK;chr20:48727637:48764201:Adrenal Gland;chr20:48737236:48789971:Brain Anterior Caudate;chr20:48737524:48768213:LNCaP;chr20:48746278:48789944:Lung;chr20:48746381:48783848:K562;chr20:48753218:48783657:CD14;chr20:48753257:48789903:HMEC;chr20:48754340:48789912:HUVEC;chr20:48768265:48783648:HepG2;chr20:48781955:48812547:Adrenal Gland;chr20:48802930:48844173:Adipose Nuclei;chr20:48802979:48811786:VACO 503;chr20:48803479:48814402:K562;chr20:48803633:48810228:VACO 400;chr20:48805257:48814437:Lung;chr20:4880560

ASHG19SUPERLNC2A100038277	2.1744	down	noncoding	ENST00000499521	noncoding	ENSG00000230551	CTB-89H12.4		8636	chr5:148873877-148884233:-	chr5:148819120:148837341:K562;chr5:148819269:148881410:Panc1;chr5:148819274:148868596:Colon Crypt 1;chr5:148819404:148867311:Sigmoid Colon;chr5:148819616:148866925:Colon Crypt 2;chr5:148821819:148834385:Stomach Smooth Muscle;chr5:148824432:148845358:IMR90;chr5:148824720:148847073:HSMMtube;chr5:148853356:148869916:HMEC;chr5:148853560:148870207:NHEK;chr5:148853650:148877537:HCC1954;chr5:148860541:148943578:Adipose Nuclei;chr5:148860971:148879463:NHDF-Ad;chr5:148863775:148897937:NHLF;chr5:148864014:148944758:Osteoblasts;chr5:148864055:148882587:HSMMtube;chr5:148893794:148943640:CD4p CD25- Il17- PMAstim Th;chr5:148897743:148932547:CD34 Primary RO01536;chr5:148922338:148943852:Duodenum Smooth Muscle;chr5:148922454:148943762:Fetal Intestine;chr5:148922503:148944671:HUVEC;chr5:148923119:148943190:Spleen;chr5:148923133:148932151:CD34 Primary RO01549;chr5:148923224:148946097:Esophagus;chr5:148924664:149021152:Gastric;chr5:148926288:148944800:NHEK;chr5:148928214:148941668:CD4 Memory Primary 7pool;chr5:148928230:148944907:HMEC;chr5:148928279:148944100:NHDF-Ad;chr5:148928323:148932047:CD34 Primary RO01480;chr5:148928371:148943685:NHLF;chr5:148928400:148943524:Astrocytes;chr5:148928411:148
ASHG19SELNC2A100014649	2.4955	up	noncoding	ENST00000605480	noncoding	ENSG00000271631	RP11-408O19.5	Reliable	610	chr9:115647438-115648047:+	chr9:115593194:115665674:CD14

ASHG19SELNC2A100126370	3.6335	up	noncoding	ENST000003599 06	noncodi ng	ENSG000001 72250	SERHL		1346	chr22:42896596-42908566:+	chr22:42878327:42932916:Brain Cingulate Gyrus;chr22:42896102:42933228:Brain Hippocampus Middle
ASHG19SELNC2A100016212	2.0868	up	noncoding	ENST0000060654 0	noncodi ng	ENSG000002 72347	RP11-43F13.4	Reliable	1585	chr5:1005154-1006738:+	chr5:943372:968917:Toledo;chr5:955704:998713:Jurkat;chr5:989435:1013448:Toledo

ASHG19SELNC2A100652033	2.2052	down	noncoding	ENST00000499194	noncoding	ENSG00000244926	ALKBH3-AS1	1906	chr11:43930842-43942476:-	chr11:43925695:43966804:Duodenum Smooth Muscle;chr11:43926240:43969863:Small Intestine;chr11:43926449:43969198:Ovary;chr11:43928048:43945475:MM1S;chr11:43937958:43969840:Sigmoid Colon;chr11:43941179:43971019:Aorta
ASHG19SELNC2A100015739	4.4629	down	noncoding	ENST00000441895	noncoding	ENSG00000225860	RP11-1018N14.2	2645	chr17:49414076-49419932:+	chr17:49412404:49452376:Brain Hippocampus Middle 150;chr17:49412535:49452208:Brain Inferior Temporal Lobe;chr17:49412663:49451710:Brain Cingulate Gyrus

ASHG19SELNC2A100067244	2.4572	down	noncoding	ENST00000601116	noncoding	ENSG00000268027	AC006129.2	601	chr19:42055888-42057073:+	chr19:42021734:42068710:CD20;chr19:42022136:42062854:CD19 Primary;chr19:42027069:42067869:CD4p CD25- Il17- PMAstim Th;chr19:42027185:42067177:CD56;chr19:42027237:42072222:CD4p CD25- CD45RAp Naive;chr19:42027259:42063702:MM1S;chr19:42027300:42072211:Tonsil;chr19:42027400:42070354:Toledo;chr19:42027607:42066875:CD4p CD25- Il17p PMAstim Th17

ASHG19SELNC2A100018110	2.6312	up	noncoding	ENST000004313 76	noncoding	ENSG00000230730	AC074011.2	Reliable	581	chr2:28856148-28887406:-	chr2:28789785:28861038:Psoas Muscle;chr2:28804678:28866634:Pancreatic islets;chr2:28805268:28860190:Skeletal Muscle;chr2:28806133:28848582:HeLa;chr2:28806193:28863417:CD14;chr2:28806196:28868492:Left Ventricle;chr2:28806225:28861087:Lung;chr2:28806226:28868492:Right Atrium;chr2:28806276:28890142:Esophagus;chr2:28806330:28888847:Sigmoid Colon;chr2:28806480:28907005:Aorta;chr2:28806642:28907013:Small Intestine;chr2:28806722:28906994:Stomach Smooth Muscle;chr2:28806770:28889182:Duodenum Smooth Muscle;chr2:28825474:28849071:CD34 Primary RO01536;chr2:28832761:28873406:HMEC;chr2:28833216:28888249:Colon Crypt 1;chr2:28835613:28850390:CD4p CD25- CD45ROp Memory;chr2:28835773:28849201:CD4 Memory Primary 7pool;chr2:28835860:28848887:CD4p CD225int CD127p Tmem;chr2:28835937:28848980:CD3;chr2:28835981:28848233:CD4 Naive Primary 7pool;chr2:28837043:28886251:VACO 9m;chr2:28843165:28898259:Gastric;chr2:28843336:28888581:Ovary;chr2:28843402:28889028:Fetal Intestine;chr2:28843662:28916085:HSMMtube;chr2:28862226:28887508:HeLa;chr2:28865636:28889058:Fetal Intestine Large;chr2:28883962:28976455:Left Ventricle;chr2:28939958:28981783:Aorta;chr2:28970154:28976795:CD4p CD25- CD45RAp

ASHG19SELNC2A100008326	4.5601	down	noncoding	ENST000005099 32	noncoding	ENSG00000250125	RP11-707A18.1	4079	chr4:65779999-65870218:-	chr4:65832382:65858351:Ly3

ASHG19SELNC2A100018246	3.117	down	noncoding	ENST00000501122	noncoding	ENSG00000245532	NEAT1	22743	chr11:65190269-65213011:+	chr11:65124427:65154947:Fetal Intestine Large;chr11:65124669:65154911:Fetal Intestine;chr11:65133472:65159026:HeLa;chr11:65134015:65159000:Pancreas;chr11:65137968:65159071:NHDF-Ad;chr11:65138031:65158188:CD4 Memory Primary 7pool;chr11:65144518:65155720:Pancreatic islets;chr11:65145326:65155010:CD56;chr11:65145999:65161052:Brain Hippocampus Middle;chr11:65146765:65153405:Small Intestine;chr11:65146897:65153444:Lung;chr11:65147327:65155112:Left Ventricle;chr11:65148732:65154998:CD8 primiary;chr11:65148822:65154999:Spleen;chr11:65148895:65158002:CD4p CD25- Il17p PMAstim Th17;chr11:65149077:65153410:Sigmoid Colon;chr11:65157526:65198662:Fetal Muscle;chr11:65157742:65225276:DHL6;chr11:65167786:65223197:Colon Crypt 2;chr11:65168731:65223379:CD20;chr11:65168909:65198287:CD34 Primary RO01549;chr11:65169738:65223378:Ly4;chr11:65170357:65224596:Tonsil;chr11:65170521:65225513:GM12878;chr11:65170621:65223375:CD56;chr11:65170629:65223372:CD19 Primary;chr11:65170631:65223404:CD14;chr11:65170783:65198465:Ly3;chr11:65170809:65225246:Ly1;chr11:65170818:65198139:K562;chr11:65170820:65199975:HBL1;chr11:65170828:65199000:Fetal Thymus;chr11:65170903:65198766:Thymus;chr11:65170913:65198109:CD34 Primary

ASHG19SELNC2A100017427	2.417	up	noncoding	ENST00000437680	noncoding	ENSG00000237133	AC020594.5	Gold	338	chr2:33780114-33788614:-	chr2:33666722:33710698:Skeletal Muscle;chr2:33699374:33790617:CD20;chr2:33699502:33727719:Ly3;chr2:33699588:33737763:DHL6;chr2:33699635:33724391:MM1S;chr2:33699875:33743353:Brain Cingulate Gyrus;chr2:33699893:33727679:CD19 Primary;chr2:33699905:33724425:HBL1;chr2:33699910:33748217:Brain Hippocampus Middle 150;chr2:33699923:33725484:GM12878;chr2:33699968:33727830:Tonsil;chr2:33700094:33736889:Brain Anterior Caudate;chr2:33750720:33761266:DHL6;chr2:33750850:33759069:CD19 Primary;chr2:33750852:33769157:Ly3

ASHG19SELNC2A100010807	2.8396	up	noncoding	ENST00000607715	noncoding	ENSG00000271788	CTD-2201E18.5	Reliable	811	chr5:43006835-43007645:-	chr5:42974744:43010144:Astrocytes;chr5:42982065:43021566:CD4 Memory Primary 7pool;chr5:42982072:43021311:CD34 Primary RO01536;chr5:42982188:43021349:CD8 Memory 7pool;chr5:42982625:43009005:CD34 adult;chr5:42982779:43008925:CD34 fetal;chr5:42984005:43021232:HeLa;chr5:42984195:43020926:Esophagus;chr5:42984237:43021003:Osteoblasts;chr5:42984869:43020974:NHLF;chr5:42985069:43010090:VACO 9m;chr5:42987481:42997335:CD8 primiary;chr5:42988207:43021793:CD56;chr5:42988303:42997081:CD4p CD25- Il17- PMAstim Th;chr5:42988340:43022194:CD4p CD25- CD45ROp Memory;chr5:42988541:42996957:CD4p CD25- CD45RAp Naive;chr5:42988679:43021490:CD4 Naive Primary 8pool;chr5:42988731:43021440:CD8 Naive 8pool;chr5:42988739:43021396:CD8 Naive 7pool;chr5:42988783:43021518:CD3;chr5:42988888:43021861:CD4p CD25- Il17p PMAstim Th17;chr5:42988915:43022042:Tonsil;chr5:42988940:43021536:CD4p CD225int CD127p Tmem;chr5:42989202:42996790:HUVEC;chr5:42989365:42996520:CD20;chr5:42989548:43021294:CD4 Naive Primary 7pool;chr5:42989568:43021469:CD4 Memory Primary 8pool;chr5:42989815:43020989:Spleen;chr5:42989931:43020950:Sigmoid Colon;chr5:42989941:43020943:Small

ASHG19SELNC2A100012859	7.4912	down	noncoding	ENST0000432488	noncoding	ENSG00000235492	RP11-16L9.4	662	chr1:198985262-199045864:+	chr1:198842777:199083902:DND41;chr1:198857113:198958876:RPMI-8402;chr1:198871424:198907549:Brain Inferior Temporal Lobe;chr1:198871577:198909555:Toledo;chr1:198888066:198907526:Tonsil;chr1:198975663:199017602:RPMI-8402;chr1:198975672:199016988:Jurkat;chr1:198975711:199016609:Toledo;chr1:198977505:199038787:Fetal Thymus

ASHG19SELNC2A100934297	2.2736	up	noncoding	ENST00000551503	noncoding	ENSG00000257261	RP11-96H19.1		Reliable	829		chr12:46777462-46850051:+	chr12:46745509:46786096:Adipose Nuclei;chr12:46750690:46781406:HMEC;chr12:46750696:46801411:Osteoblasts;chr12:46750818:46797247:NHEK;chr12:46751076:46781994:Left Ventricle;chr12:46752905:46785767:CD20;chr12:46754023:46782918:CD14;chr12:46754278:46783404:CD19 Primary;chr12:46755152:46786747:Panc1;chr12:46758395:46797876:Duodenum Smooth Muscle;chr12:46758596:46785607:HUVEC;chr12:46758835:46779688:Brain Hippocampus Middle 150;chr12:46759700:46781118:CD4 Memory Primary 7pool;chr12:46760895:46797996:HeLa;chr12:46760905:46783619:NHDF-Ad;chr12:46760966:46798138:NHLF;chr12:46761022:46798920:Tonsil;chr12:46761111:46785610:Skeletal Muscle;chr12:46761122:46795854:Lung;chr12:46761154:46781460:CD34 Primary RO01536;chr12:46761219:46797045:Colon Crypt 1;chr12:46761973:46778834:CD34 Primary RO01480;chr12:46762301:46780693:HCT-116;chr12:46762605:46799048:IMR90;chr12:46762740:46799125:Astrocytes;chr12:46762818:46799053:HSMM;chr12:46763090:46784642:Colon Crypt 3;chr12:46763376:46781577:u87;chr12:46763621:46798940:Skeletal Muscle Myoblast;chr12:46763715:46785789:Toledo;chr12:46763765:46785105:Fetal

ASHG19SELNC2A100934297	2.2736	up	noncoding	ENST00000551503	noncoding	ENSG00000257261	RP11-96H19.1	Reliable	829	chr12:46777462-46850051:+	chr12:46822847:46889265:HeLa;chr12:46822865:46889072:HUVEC;chr12:46822900:46887936:HCT-116;chr12:46823019:46848340:HMEC;chr12:46823047:46850701:NHEK;chr12:46865752:46889668:IMR90
ASHG19SELNC2A100011127	3.4199	down	noncoding	ENST00000440570	noncoding	ENSG00000223749	MIR503HG		760	chrX:133677367-133680662:-	chrX:133676035:133689032:Fetal Muscle
ASHG19SELNC2A100015425	2.4506	up	noncoding	ENST00000610001	noncoding	ENSG00000272970	RP11-329B9.4	Reliable	701	chr3:184451863-184456737:+	chr3:184274276:184303185:Gastric;chr3:184279295:184303121:Esophagus;chr3:184280246:184445487:Fetal Muscle;chr3:184281212:184287586:LNCaP;chr3:184281285:184322501:Ovary;chr3:184444168:184485359:HMEC;chr3:184454658:184491921:Esophagus;chr3:184455452:184492084:Gastric

ASHG19SELNC2A100018084	3.4376	up	noncoding	ENST00000415809	noncoding	ENSG00000228661	AC090587.5	Gold	494	chr11:3875548-3876739:-	chr11:3828661:3863671:Brain Hippocampus Middle;chr11:3844341:3863605:Brain Cingulate Gyrus;chr11:3844611:3863500:Brain Anterior Caudate;chr11:3845140:3898531:CD4p CD25- CD45RAp Naive;chr11:3845158:3863281:Brain Inferior Temporal Lobe;chr11:3845193:3980272:CD14;chr11:3845355:3899942:CD4 Memory Primary 7pool;chr11:3845384:3863637:Brain Hippocampus Middle 150;chr11:3845410:3902515:CD8 primiary;chr11:3845421:3971821:CD20;chr11:3845430:3883033:CD4p CD25- CD45ROp Memory;chr11:3845565:3942226:CD56;chr11:3845759:3902508:CD4p CD25- Il17- PMAstim Th;chr11:3845795:3900261:K562;chr11:3845961:3927658:Spleen;chr11:3846088:3902358:CD34 Primary RO01536;chr11:3851143:3863755:Ly4;chr11:3851589:3892955:CD4p CD25- Il17p PMAstim Th17;chr11:3851747:3899797:CD8 Memory 7pool;chr11:3851899:3902363:Sigmoid Colon;chr11:3851899:3902446:Small Intestine;chr11:3852368:3916127:Duodenum Smooth Muscle;chr11:3852445:3930490:Adipose Nuclei;chr11:3852540:3863525:CD34 Primary RO01480;chr11:3852790:3887721:CD3;chr11:3852946:3899571:Esophagus;chr11:3853570:3882689:CD8 Naive 8pool;chr11:3855274:3864019:Tonsil;chr11:3855410:3889590:Jurkat;chr11:3855469:3882443:CD4p

ASHG19SELNC2A100002924	3.7377	up	noncoding	NR_125398	noncoding	ENSG00000244300	RP11-475N22.4	Gold	2358	chr3:128208046-128222050:+	chr3:128198402:128209972:Spleen;chr3:128198463:128215333:CD34 Primary RO01536;chr3:128199015:128216666:Lung;chr3:128199025:128216656:LNCaP;chr3:128199676:128211913:HUVEC;chr3:128200879:128224614:K562;chr3:128201129:128212495:CD34 Primary RO01480;chr3:128203519:128216818:VACO 503

ASHG19SELNC2A100214214	10.237	down	noncoding	NR_147027	noncoding	ENSG00000065357	DGKA	3005	chr12:56325812-56347807:+	chr12:56292202:56332486:CD4 Memory Primary 7pool;chr12:56302603:56334475:HMEC;chr12:56306687:56352905:CD4p CD25- Il17- PMAstim Th;chr12:56307923:56334720:Spleen;chr12:56314637:56334486:Ly1;chr12:56318608:56335563:CD4p CD25- Il17p PMAstim Th17;chr12:56318620:56334637:Toledo;chr12:56318690:56337112:CD4p CD25- CD45RAp Naive;chr12:56318758:56335005:DND41;chr12:56318932:56334295:Fetal Thymus;chr12:56319159:56334646:Tonsil;chr12:56319466:56334663:CD8 Naive 8pool;chr12:56319590:56334983:Esophagus;chr12:56319793:56330449:CD4 Memory Primary 8pool;chr12:56320106:56330422:CD4 Naive Primary 7pool;chr12:56322364:56334228:Thymus;chr12:56322937:56334125:NHEK

ASHG19SELNC2A100008492	2.7496	down	noncoding	ENST00000608884	noncoding	ENSG00000273341	RP5-899E9.1	733	chr7:77045990-77054760:+	chr7:77010620:77111780:CD14;chr7:77016591:77056363:HepG2;chr7:77036386:77095861:u87;chr7:77054037:77097807:HSMMtube;chr7:77054421:77095171:HSMM;chr7:77054423:77094996:Skeletal Muscle Myoblast

ASHG19SELNC2A100007404	2.292	up	noncoding	ENST000004550 74	noncoding	ENSG00000234789	RP11-483H20.4	Reliable	470	chr9:132402755-132403561:+	chr9:132307310:132401372:HCT-116;chr9:132309709:132335646:Brain Hippocampus Middle;chr9:132310209:132334432:Ovary;chr9:132310862:132333634:Brain Mid Frontal Lobe;chr9:132318659:132373604:VACO 400;chr9:132329526:132339892:u87;chr9:132345516:132373414:HepG2;chr9:132346510:132400220:Spleen;chr9:132357063:132374195:Gastric;chr9:132388245:132417334:Adrenal Gland

ASHG19SELNC2A100201998	4.2045	up	noncoding	ENST00000579458	noncoding	ENSG00000265369	U3	2598	chr18:24267585-24283602:-	chr18:24264779:24297524:RPMI-8402;chr18:24275980:24297873:DND41
ASHG19SELNC2A101019206	2.487	up	noncoding	ENST00000457510	noncoding	ENSG00000241764	AC002467.7	657	chr7:107383262-107384573:-	chr7:107381985:107387665:CD8 primiary;chr7:107382877:107389728:CD4p CD25-Il17p PMAstim Th17
ASHG19SELNC2A100296803	5.8361	down	noncoding	MICT00000158378	noncoding	CATG00000035089	CATG00000035089.1	4250	chr18:8949324-8993838:+	chr18:8937264:8953874:HSMM;chr18:8942949:8954393:NHDF-Ad;chr18:8943424:8953874:Skeletal Muscle Myoblast

ASHG19SELNC2A100296803	5.8361	down	noncoding	MICT000001583 78	noncoding	CATG00000035089	CATG00000035089.1	4250	chr18:8949324-8993838:+	chr18:8982364:9023448:u87;chr18:8982365:9023286:CD4 Memory Primary 7pool;chr18:8993682:9023368:CD8 Memory 7pool;chr18:8993940:9023254:K562
ASHG19SELNC2A101120258	2.9811	up	noncoding	ENST00000602478	noncoding	ENSG00000270022	RNU12	664	chr22:43011250-43011913:+	chr22:42980294:43018064:Lung;chr22:42997730:43020259:Spleen;chr22:43004520:43020614:Sigmoid Colon;chr22:43004823:43012398:CD34 Primary RO01536;chr22:43008667:43012611:CD8 Memory 7pool;chr22:43008826:43012539:CD4 Memory Primary 7pool

ASHG19SELNC2A100003756	4.1115	down	noncoding	ENST00000526611	noncoding	ENSG00000246982	RP1-179N16.6	587	chr6:36114475-36164980:-	chr6:35982015:36096681:CD14;chr6:35994969:36046831:CD34 Primary RO01536;chr6:35995126:36007828:CD34 Primary RO01549;chr6:35995354:36008175:CD34 Primary RO01480;chr6:36058244:36115204:CD4p CD25- Il17- PMAstim Th;chr6:36061446:36100892:CD20;chr6:36062072:36091854:DHL6;chr6:36062112:36100843:Tonsil;chr6:36062202:36110219:CD8 primiary;chr6:36062310:36100665:Sigmoid Colon;chr6:36062352:36104672:Ly4;chr6:36062393:36091739:Jurkat;chr6:36065757:36110004:CD56;chr6:36065879:36091862:Fetal Thymus;chr6:36067006:36091887:CD19 Primary;chr6:36067039:36091884:CD3;chr6:36067089:36089645:Thymus;chr6:36082566:36100282:Colon Crypt 2;chr6:36084612:36088861:CD34 adult;chr6:36085071:36102084:Fetal Intestine;chr6:36085398:36102228:Fetal Intestine Large;chr6:36088692:36118702:Adrenal Gland;chr6:36097600:36100433:VACO 400
ASHG19SELNC2A100005527	2.3619	down	noncoding	MICT00000282217	noncoding	CATG00000079741	CATG00000079741.1	4296	chr5:52593702-52619385:-	chr5:52613699:52675409:HMEC;chr5:52613974:52640087:NHEK;chr5:52616175:52675561:u87;chr5:52621713:52735533:Osteoblasts;chr5:52655268:52675314:NHEK;chr5:52655451:52727176:NHLF;chr5:52691931:52727310:u87;chr5:52692543:52726861:HeLa;chr5:52692775:52722046:NHDF-Ad;chr5:52693831:52726798:HMEC;chr5:52701847:52726740:NHEK

ASHG19SELNC2A100016135	3.0776	up	noncoding	ENST00000561761	noncoding	ENSG00000261220	RP11-629O1.2	Reliable	679	chr8:134585426-134586104:+	chr8:134467923:134561478:CD4p CD25- Il17-PMAstim Th;chr8:134477147:134543390:CD4p CD25- CD45RAp Naive;chr8:134478014:134584309:CD8 primiary;chr8:134479403:134540188:CD8 Naive 8pool;chr8:134485145:134518237:CD20;chr8:134485660:134546122:CD4p CD25- Il17p PMAstim Th17;chr8:134490651:134585327:Adrenal Gland;chr8:134494104:134561290:CD4 Memory Primary 7pool;chr8:134494808:134563457:Adipose Nuclei;chr8:134495384:134552578:Brain Inferior Temporal Lobe;chr8:134495803:134517089:Ovary;chr8:134496520:134587951:Lung;chr8:134496637:134588231:Left Ventricle;chr8:134496712:134550611:Brain Anterior Caudate;chr8:134496813:134587940:Right Atrium;chr8:134497072:134518457:CD34 Primary RO01480;chr8:134497139:134544764:Tonsil;chr8:134497225:134586114:Psoas Muscle;chr8:134497326:134561099:Small Intestine;chr8:134497349:134585225:CD34 Primary RO01536;chr8:134497358:134544690:CD4p CD25- CD45ROp Memory;chr8:134497368:134541302:CD56;chr8:134497637:134585612:Spleen;chr8:134497849:134552961:CD34 Primary RO01549;chr8:134497867:134540128:CD3;chr8:134497913:134540282:Sigmoid Colon;chr8:134497980:134518247:CD8 Naive 7pool;chr8:134498219:134585268:Gastric;chr8:1345
ASHG19SELNC2A100361869	2.1239	down	noncoding	ENST00000514519	noncoding	ENSG00000249731	RP11-259O2.3		560	chr5:1968208-1969127:+	chr5:1930374:1976494:HMEC;chr5:1935720:1975998:NHEK

ASHG19SELNC2A100018437	4.6799	down	noncoding	ENST0000571890	noncoding	ENSG00000263312	RP11-459C13.1	Reliable	549	chr17:3880397-3885193:+	chr17:3780822:3821212:NCI-H82;chr17:3782275:3834937:H2171;chr17:3785708:3797716:Pancreatic islets;chr17:3788616:3800500:LNCaP;chr17:3788828:3800155:Adrenal Gland;chr17:3789755:3800117:HeLa;chr17:3798207:3808782:Pancreas;chr17:3807098:3872953:CD20;chr17:3807403:3821299:CD19 Primary;chr17:3813770:3833405:CD34 Primary RO01480;chr17:3859604:3882957:Colon Crypt 1;chr17:3862172:3882989:Sigmoid Colon;chr17:3862322:3890678:Ly4;chr17:3862373:3893138:Gastric;chr17:3862385:3887616:Colon Crypt 3;chr17:3862399:3882040:Colon Crypt 2
ASHG19SELNC2A100000533	2.0708	up	noncoding	ENST0000423149	noncoding	ENSG00000242753	RP11-716O23.2	Reliable	686	chr6:11487459-11515943:+	chr6:11514469:11582667:CD14
ASHG19SELNC2A100000654	2.2918	down	noncoding	ENST0000536492	noncoding	ENSG00000256237	RP11-434C1.2	Reliable	188	chr12:11694329-11708772:-	chr12:11697180:11734342:GM12878

ASHG19SELNC2A100741866	6.1669	down	noncoding	ENST00000413483	noncoding	ENSG00000238184	CD81-AS1	1460	chr11:2349979-2399222:-	chr11:2379166:2423231:Toledo;chr11:2384244:2423032:Fetal Muscle;chr11:2386580:2423670:Brain Cingulate Gyrus;chr11:2386845:2423552:Brain Hippocampus Middle;chr11:2386961:2423243:Brain Hippocampus Middle 150;chr11:2387120:2422815:Spleen;chr11:2387137:2423018:Left Ventricle;chr11:2387292:2442798:Pancreatic islets;chr11:2388166:2423432:Brain Inferior Temporal Lobe;chr11:2388513:2422861:Right Atrium;chr11:2394892:2423073:Lung;chr11:2395526:2405370:Pancreas;chr11:2395645:2421627:Right Ventricle;chr11:2396049:2421634:LNCaP;chr11:2398060:2423320:Tonsil;chr11:2398089:2423448:DHL6;chr11:2398408:2423549:Esophagus;chr11:2398461:2423269:Stomach Smooth Muscle;chr11:2398466:2421655:Adrenal Gland;chr11:2398488:2421768:Gastric;chr11:2398538:2422923:Sigmoid Colon;chr11:2398637:2421637:Ovary;chr11:2398638:2422699:Small Intestine;chr11:2398650:2421664:Bladder;chr11:2398654:2423526:HUVEC;chr11:2398720:2421659:Aorta;chr11:2398754:2423055:Brain Anterior Caudate;chr11:2398764:2421885:NHLF;chr11:2398920:2423041:CD8 primiary;chr11:2400950:2421538:Brain Mid Frontal Lobe

ASHG19SELNC2A100508698	2.5289	down	noncoding	ENST0000043969 4	noncoding	ENSG00000234352	hsa-mir-490	2160	chr7:136468822-136866518:-	chr7:136756737:136770185:IMR90
ASHG19SELNC2A100018210	6.42	down	noncoding	ENST0000060742 6	noncoding	ENSG00000240207	RP11-379F4.4	2185	chr3:158453510-158455694:+	chr3:158417299:158446457:Osteoblasts;chr3:158418055:158446367:u87;chr3:158428493:158509261:HCC1954
ASHG19SELNC2A100003595	2.3974	up	noncoding	ENST0000045239 1	noncoding	ENSG00000229278	RP11-483F11.7	303	chr10:101411746-101413662:-	chr10:101413386:101450232:NHEK;chr10:101413737:101446675:HMEC;chr10:101417897:101470925:Osteoblasts

ASHG19SELNC2A100595013	4.3115	up	noncoding	ENST0000043909	noncoding	ENSG00000228221	LINC00578	542	chr3:177159752-177470093:+	chr3:177137525:177182475:HBL1;chr3:177161083:177176223:HMEC;chr3:177166709:177209811:HSMM;chr3:177166755:177212994:HSMMtube;chr3:177167294:177209811:Skeletal Muscle Myoblast

ASHG19SELNC2A100595013	4.3115	up	noncoding	ENST00000439009	noncoding	ENSG00000228221	LINC00578	542	chr3:177159752-177470093:+	chr3:177239011:177247462:HCC1954

ASHG19SELNC2A100595013	4.3115	up	noncoding	ENST00000439009	noncoding	ENSG00000228221	LINC00578	542	chr3:177159752-177470093:+	chr3:177254857:177274845:MM1S

ASHG19SELNC2A100595013	4.3115	up	noncoding	ENST00000439009	noncoding	ENSG00000228221	LINC00578	542	chr3:177159752-177470093:+	chr3:177286633:177337735:Esophagus;chr3:177306147:177341813:HCC1954;chr3:177306304:177321606:HMEC
ASHG19SELNC2A100008480	4.2758	up	noncoding	ENST00000606489	noncoding	ENSG00000272088	RP11-168F9.2	382	chr1:3403810-3404191:+	chr1:3369291:3379649:Colon Crypt 1;chr1:3369308:3400613:Colon Crypt 3;chr1:3369766:3400716:Gastric;chr1:3369826:3378616:Sigmoid Colon;chr1:3369862:3389121:HepG2;chr1:3369890:3388971:VACO 400;chr1:3370110:3400518:Colon Crypt 2;chr1:3370203:3378252:Small Intestine;chr1:3398373:3429749:VACO 503

ASHG19SELNC2A100905285	2.6453	up	noncoding	NR_104286	noncoding	ENSG00000138867	GUCD1	3265	chr22:24936391-24951903:-	chr22:24895818:24960637:VACO 400;chr22:24917481:24947188:LNCaP;chr22:24937896:24975073:Lung;chr22:24937929:24952442:Gastric;chr22:24938005:24963662:Left Ventricle;chr22:24944365:24951291:Colon Crypt 1;chr22:24944429:24952524:Small Intestine;chr22:24944466:24952522:Sigmoid Colon;chr22:24944940:24975971:Fetal Intestine;chr22:24945007:24976085:Fetal Intestine Large;chr22:24966991:24976529:HepG2;chr22:24972431:24977489:NHEK;chr22:24973912:24998878:VACO 400

ASHG19SELNC2A100008186	2.1942	down	noncoding	ENST00000418554	noncoding	ENSG00000237243	AC022173.2	577	chr7:137638094-137642712:+	chr7:137547837:137685439:Aorta;chr7:137553704:137584984:u87;chr7:137554800:137687567:Adipose Nuclei;chr7:137555121:137577100:HSMMtube;chr7:137555122:137694058:Osteoblasts;chr7:137557067:137568482:Astrocytes;chr7:137593088:137639682:NHLF;chr7:137604649:137687377:Duodenum Smooth Muscle;chr7:137607422:137687098:Astrocytes;chr7:137607525:137687119:Skeletal Muscle Myoblast;chr7:137607525:137687119:HSMM;chr7:137617110:137693771:K562;chr7:137617125:137687164:Small Intestine;chr7:137617129:137687914:Fetal Intestine Large;chr7:137619190:137688004:Fetal Intestine;chr7:137620547:137687600:HSMMtube;chr7:137636282:137693717:Esophagus;chr7:137637016:137694029:u87;chr7:137637809:137688187:HUVEC;chr7:137642825:137686950:Adrenal Gland;chr7:137651409:137687395:Brain Hippocampus Middle;chr7:137654671:137687008:NHLF;chr7:137654894:137687749:Fetal Muscle;chr7:137655243:137687096:Gastric;chr7:137655346:137685403:Ovary;chr7:137658847:137687032:NHDF-Ad;chr7:137659004:137687209:Sigmoid Colon;chr7:137663061:137686865:IMR90;chr7:137663463:137694162:HBL1;chr7:137663926:137675940:Adipose Tissue;chr7:137664409:137678967:Toledo;chr7:137664421:137694313:Ly3;chr7:137667788:137686994:HepG2

ASHG19SELNC2A100012118	3.8577	down	noncoding	ENST0000442605	noncoding	ENSG00000235772	AP001625.6	Gold	575	chr21:43980484-43982044:-	chr21:43730630:43758781:Fetal Intestine;chr21:43730685:43758943:Fetal Intestine Large;chr21:43757982:43806895:Gastric;chr21:43780529:43802188:MCF-7;chr21:43799180:43852233:Ly4;chr21:43803401:43822621:Fetal Intestine Large;chr21:43804333:43884935:CD4p CD25- Il17-PMAstim Th;chr21:43811298:43847226:RPMI-8402;chr21:43812665:43860867:CD4p CD25- Il17p PMAstim Th17;chr21:43812947:43846499:Thymus;chr21:43817601:43885276:CD4p CD25- CD45RAp Naive;chr21:43821017:43884964:CD4 Memory Primary 7pool;chr21:43821140:43847081:CD4p CD25- CD45ROp Memory;chr21:43822733:43849247:DND41;chr21:43823272:43846161:CD4p CD225int CD127p Tmem;chr21:43823401:43845869:CD3;chr21:43823558:43846366:CD4 Naive Primary 8pool;chr21:43869691:43914914:Fetal Intestine Large;chr21:43869692:43913942:Colon Crypt 2;chr21:43873255:43914930:Fetal Intestine;chr21:43875255:43914249:Small Intestine;chr21:43876507:43895539:Sigmoid Colon;chr21:43877926:43913973:Colon Crypt 1;chr21:43879292:43899707:Ly3;chr21:43905520:43968853:CD34 Primary RO01536;chr21:43923342:43955458:CD34 Primary RO01549;chr21:43923429:43959315:Ly1;chr21:43923441:43957929:Ly4;chr21:43932351:43960870:Panc

ASHG19SELNC2A100006111	5.6732	down	noncoding	ENST0000055822 6	noncoding	ENSG00000259503	RP11-543G18.1	Reliable	527	chr15:70613915-70619081:+	chr15:70527791:70578137:Ly3;chr15:70538923:70621552:Ly4
ASHG19SELNC2A100000375	2.2308	down	noncoding	ENST0000042076 2	noncoding	ENSG00000230024	RP11-95P13.1	Reliable	451	chr1:219608494-219615984:-	chr1:219595400:219639325:u87
ASHG19SELNC2A100014651	2.3221	up	noncoding	ENST0000060741 4	noncoding	ENSG00000271780	RP11-1017G21.5	Reliable	1079	chr14:102414684-102415762:+	chr14:102413635:102440255:VACO503;chr14:102413638:102448327:NHDF-Ad;chr14:102413684:102439387:Colon Crypt 3

ASHG19SELNC2A100426625	2.3754	down	noncoding	ENST0000050722 2	noncoding	ENSG00000250095	NREP-AS1		610	chr5:111248205-111353003:+	chr5:111262196:111343599:Osteoblasts;chr5:111318991:111342063:Astrocytes
ASHG19SELNC2A100571319	2.3459	down	noncoding	ENST0000041827 3	noncoding	ENSG00000272734	ADIRF-AS1		2627	chr10:88725497-88731068:-	chr10:88719833:88732392:Ly3;chr10:88725396:88733241:HCT-116;chr10:88725450:88733264:Aorta;chr10:88726022:88733482:Lung;chr10:88726149:88733229:VACO 503;chr10:88726170:88733391:Esophagus;chr10:88726179:88734770:NHEK;chr10:88727449:88732182:Ovary;chr10:88727564:88732348:NHLF
ASHG19SELNC2A100010015	12.685	up	noncoding	ENST0000057696 3	noncoding	ENSG00000262772	RP11-353N14.2	Reliable	1788	chr17:77797251-77801503:+	chr17:77760586:77818726:LNCaP;chr17:77763821:77789395:VACO 503;chr17:77770172:77788111:GLC16;chr17:77773273:77827285:Toledo;chr17:77775993:77811118:K562;chr17:77802901:77825355:Gastric;chr17:77804578:77818301:Pancreas;chr17:77804734:77826977:Esophagus;chr17:77805883:77825877:VACO 503;chr17:77809448:77827375:Fetal Intestine

ASHG19SELNC2A100016998	9.062	up	noncoding	ENST00000602192	noncoding	ENSG00000267868	RP11-120K24.3	Reliable	1297	chr13:113619149-113620445:-	chr13:113528283:113706264:Pancreatic islets;chr13:113612236:113642719:Lung;chr13:113612250:113641315:Left Ventricle;chr13:113612285:113757507:Brain Hippocampus Middle;chr13:113633553:113668596:Gastric;chr13:113635638:113668660:Colon Crypt 3;chr13:113636776:113657006:Colon Crypt 1;chr13:113636800:113656732:Colon Crypt 2;chr13:113637770:113669757:LNCaP;chr13:113637848:113668615:VACO 400;chr13:113650879:113757822:Brain Inferior Temporal Lobe;chr13:113688788:113757608:Brain Cingulate Gyrus;chr13:113689195:113739320:Brain Anterior Caudate;chr13:113694428:113748561:Brain Hippocampus Middle 150;chr13:113695156:113757432:Brain Angular Gyrus;chr13:113698306:113718391:Brain Mid Frontal Lobe

ASHG19SELNC2A100868230	2.1217	up	noncoding	ENST000004120 68	noncoding	ENSG00000223907	RP11-439L8.4	2741	chr1:31984036-31989846:+	chr1:31944906:31977293:Toledo;chr1:31948563:31981686:DND41;chr1:31974167:32058180:Fetal Intestine;chr1:31974366:32058187:Colon Crypt 1;chr1:32001720:32055384:Small Intestine;chr1:32014154:32058191:VACO 503;chr1:32028014:32063653:HUVEC;chr1:32029384:32058106:Colon Crypt 2;chr1:32033757:32059228:Aorta;chr1:32033763:32062625:Left Ventricle;chr1:32033943:32062686:Spleen;chr1:32034109:32062571:Right Atrium;chr1:32037967:32057981:Esophagus;chr1:32038018:32055476:Lung;chr1:32038201:32058632:Stomach Smooth Muscle;chr1:32038276:32055532:Sigmoid Colon;chr1:32038364:32058589:Duodenum Smooth Muscle;chr1:32038461:32055389:Gastric;chr1:32038488:32055215:VACO 400;chr1:32038607:32054952:Pancreas
ASHG19SELNC2A100500809	2.9481	down	noncoding	ENST00000457234	noncoding	ENSG00000213904	LIPE-AS1	1501	chr19:42928421-43030021:+	chr19:42899140:42938638:Brain Hippocampus Middle;chr19:42899739:42938263:Brain Inferior Temporal Lobe;chr19:42899800:42938152:Brain Anterior Caudate;chr19:42899816:42938392:Brain Cingulate Gyrus;chr19:42899939:42942433:Adipose Nuclei;chr19:42900120:42938388:Brain Hippocampus Middle 150;chr19:42900412:42927712:Brain Angular Gyrus;chr19:42925113:42954769:Gastric;chr19:42941647:42994085:HCC1954;chr19:42984371:43004012:HepG2
ASHG19SELNC2A100006894	2.1097	up	noncoding	ENST00000563192	noncoding	ENSG00000260260	RP11-304L19.5	342	chr16:2204798-2205359:-	chr16:2195325:2205839:Pancreatic islets;chr16:2197196:2210499:HCT-116;chr16:2199546:2229416:Esophagus;chr16:2200572:2214258:Colon Crypt 2;chr16:2202672:2212123:NHLF;chr16:2202718:2214221:VACO 9m

ASHG19SELNC2A100012316	2.2498	up	noncoding	ENST0000056929 1	noncoding	ENSG00000261474	RP11-452L6.1	Reliable	449	chr16:31460856-31464814:-	chr16:31441143:31472106:Psoas Muscle;chr16:31463739:31472341:K562
ASHG19SELNC2A100013135	5.2941	up	noncoding	ENST0000060949 7	noncoding	ENSG00000272894	RP5-1159O4.1		1578	chr7:7592093-7606627:-	chr7:7604880:7614065:CD20

ASHG19SELNC2A100011348	5.043	down	noncoding	ENST000004784 68	noncoding	ENSG00000240687	RP11-521D12.1	Reliable	832	chr2:9778901-9789568:+	chr2:9765172:9817103:Panc1;chr2:9767472:9795391:Brain Anterior Caudate;chr2:9768796:9795405:Brain Inferior Temporal Lobe;chr2:9768879:9795656:Brain Hippocampus Middle;chr2:9768972:9795590:Brain Cingulate Gyrus;chr2:9768979:9795389:Brain Hippocampus Middle 150

ASHG19SELNC2A100151940	2.3196	up	noncoding	ENST00000518988	noncoding	ENSG00000253535	RP11-624C23.1		741	chr8:24153327-24245028:-	chr8:24117286:24247817:CD20;chr8:24125659:24169134:HBL1;chr8:24150260:24190101:Tonsil;chr8:24205065:24255630:Tonsil;chr8:24206035:24246836:CD19 Primary

ASHG19SELNC2A100010696	9.8906	up	noncoding	ENST00000537269	noncoding	ENSG00000257084	U47924.27	Gold	350	chr12:7072409-7073610:+	chr12:7021310:7026081:H2171;chr12:7022600:7051380:Brain Cingulate Gyrus;chr12:7022685:7051255:Stomach Smooth Muscle;chr12:7022964:7047898:GLC16;chr12:7032434:7039694:H2171;chr12:7032586:7039696:Lung;chr12:7033029:7039703:Esophagus;chr12:7033041:7048410:Right Atrium;chr12:7033067:7050686:Brain Angular Gyrus;chr12:7033253:7051404:LNCaP;chr12:7033291:7048082:Fetal Muscle;chr12:7033383:7048045:Ovary;chr12:7033514:7048028:H1;chr12:7033555:7047595:Bladder;chr12:7033612:7047812:Pancreas;chr12:7033631:7055666:VACO 503;chr12:7033645:7047280:Right Ventricle;chr12:7044264:7072113:CD14;chr12:7044426:7072735:CD34 Primary RO01536;chr12:7044970:7072648:CD19 Primary;chr12:7045010:7071657:CD4 Naive Primary 7pool;chr12:7045041:7071857:CD4p CD225int CD127p Tmem;chr12:7045046:7072000:CD3;chr12:7045125:7071857:CD8 Naive 7pool;chr12:7045276:7072350:CD56;chr12:7045399:7071773:Ly3;chr12:7045533:7071721:CD8 Naive 8pool;chr12:7045550:7072074:CD4 Naive Primary 8pool;chr12:7045555:7071876:CD4 Memory Primary 8pool;chr12:7045612:7071479:Toledo;chr12:7045636:7072391:Thymus;chr12:7045722:7069352:Ly4;chr12:7045931:7071909:GM12878;chr12:7050709:7057

ASHG19SELNC2A100010140	2.1298	up	noncoding	HBMT00000529265	noncoding	CATG00000026163	CATG00000026163.1	2947	chr16:192735-195681:+	chr16:114836:157699:Brain Hippocampus Middle;chr16:114993:164358:Brain Cingulate Gyrus;chr16:116673:152882:Brain Anterior Caudate;chr16:117014:152935:Brain Inferior Temporal Lobe;chr16:117398:143501:LNCaP;chr16:117767:135878:NHLF;chr16:120486:137250:Aorta;chr16:120741:136981:Lung;chr16:120811:153746:Left Ventricle;chr16:120938:136571:Sigmoid Colon;chr16:120960:135466:Fetal Muscle;chr16:121054:153287:Right Atrium;chr16:122601:135653:Ovary;chr16:124312:157202:Brain Angular Gyrus;chr16:152411:170614:CD34 fetal;chr16:154129:214661:CD4 Memory Primary 7pool;chr16:154617:171086:CD34 adult

ASHG19SELNC2A100004606	2.5822	up	noncoding	ENST000005424 5	noncoding	ENSG00000258560	CTD-2376I20.1	Reliable	548	chr14:100519140-100541215:-	chr14:100435239:100507436:MCF-7;chr14:100478260:100500070:Osteoblasts;chr14:100482837:100516659:HSMM;chr14:100482934:100516645:Skeletal Muscle Myoblast;chr14:100514734:100586793:DND41;chr14:100520595:100549173:CD34 Primary RO01536;chr14:100520682:100546624:CD34 Primary RO01549;chr14:100522443:100594774:CD4p CD25- Il17- PMAstim Th;chr14:100522811:100614800:CD4p CD25- CD45RAp Naive;chr14:100523024:100582652:CD4p CD25- CD45ROp Memory;chr14:100523234:100586008:CD20;chr14:100523285:100550411:Fetal Thymus;chr14:100523340:100588458:CD4 Memory Primary 7pool;chr14:100523524:100549949:Tonsil;chr14:100523769:100549857:Jurkat;chr14:100524339:100580540:CD8 primiary;chr14:100524448:100574855:CD3;chr14:100524674:100598765:CD4p CD25- Il17p PMAstim Th17;chr14:100524703:100553687:CD56;chr14:100525251:100546411:Small Intestine;chr14:100527280:100548954:Thymus;chr14:100527557:100553587:CD4p CD225int CD127p Tmem;chr14:100528449:100546559:CD8 Memory 7pool;chr14:100528456:100549806:CD4 Memory Primary 8pool;chr14:100528476:100549448:CD4 Naive Primary

ASHG19SELNC2A100186253	5.7559	down	noncoding	ENST00000598447	noncoding	ENSG00000232934	RP11-324O2.3		792	chr10:114166032-114169248:-	chr10:114089841:114099802:Fetal Intestine Large;chr10:114091751:114167822:Colon Crypt 1;chr10:114118589:114150503:HeLa;chr10:114119245:114199281:Fetal Intestine Large;chr10:114119280:114166412:HCT-116;chr10:114119392:114167923:Small Intestine;chr10:114119440:114170199:Fetal Intestine
ASHG19SELNC2A100008499	2.0033	up	noncoding	ENST00000609281	noncoding	ENSG00000273320	RP11-22N19.2	Reliable	847	chr7:105925926-105926772:+	chr7:105920170:105927354:CD4p CD25- Il17p PMAstim Th17;chr7:105921719:105932507:Fetal Intestine Large;chr7:105922548:105932471:Fetal Intestine
ASHG19SELNC2A100018023	2.2099	up	noncoding	ENST00000422554	noncoding	ENSG00000223716	RP11-113O24.3	Reliable	399	chr9:38360424-38376427:+	chr9:38373991:38395597:Fetal Intestine Large;chr9:38376064:38397910:Aorta

ASHG19SELNC2A100008929	2.9684	up	noncoding	ENST000004162 37	noncoding	ENSG00000232465	RP5-1022J11.2	Reliable	573	chr20:51104461-51117130:-	chr20:51105907:51114975:HUVEC
ASHG19SELNC2A100000773	2.8357	up	noncoding	ENST0000006047 16	noncoding	ENSG00000270605	RP5-1092A3.4	Reliable	1945	chr1:28566020-28567964:-	chr1:28561517:28594636:CD8 Memory 7pool;chr1:28561754:28594379:Fetal Intestine Large;chr1:28567934:28588340:NHEK;chr1:28570203:28594681:CD4 Memory Primary 7pool;chr1:28572835:28606098:K562

ASHG19SELNC2A100013854	3.8629	down	noncoding	ENST000005632 80	noncoding	ENSG00000260944	RP11-463O9.5	319	chr16:86598751-86601367:-	chr16:86591173:86653733:Aorta;chr16:86596520:86644503:HSMMtube;chr16:86596603:86670683:Osteoblasts;chr16:86596748:86630272:u87;chr16:86596888:86625094:HUVEC;chr16:86597302:86623506:Panc1;chr16:86598500:86625119:Astrocytes;chr16:86602139:86624292:NHLF
ASHG19SELNC2A100740135	2.476	up	noncoding	ENST00000607215	noncoding	ENSG00000235033	RP11-61I13.3	3053	chr6:39851292-39854344:-	chr6:39720961:39829617:Brain Anterior Caudate;chr6:39732733:39871672:Brain Hippocampus Middle 150;chr6:39732791:39876756:Brain Hippocampus Middle;chr6:39732850:39876155:Brain Inferior Temporal Lobe;chr6:39732882:39876926:Brain Cingulate Gyrus;chr6:39758289:39829579:Brain Angular Gyrus;chr6:39768980:39830070:Aorta;chr6:39771319:39860627:Spleen;chr6:39776033:39824152:Duodenum Smooth Muscle;chr6:39777127:39803586:Brain Mid Frontal Lobe;chr6:39832635:39860559:Jurkat;chr6:39844382:39860932:Aorta;chr6:39846831:39860544:Osteoblasts;chr6:39847655:39857422:HSMM;chr6:39847754:39857272:Skeletal Muscle Myoblast;chr6:39848548:39858066:HSMMtube

ASHG19SELNC2A100010649	2.352	down	noncoding	ENST00000507997	noncoding	ENSG00000250551	RP11-254I22.1	Reliable	704	chr5:95385819-95551223:+	chr5:95358200:95387335:u87;chr5:95358700:95385947:HMEC
ASHG19SELNC2A100010649	2.352	down	noncoding	ENST00000507997	noncoding	ENSG00000250551	RP11-254I22.1	Reliable	704	chr5:95385819-95551223:+	chr5:95400495:95433256:u87;chr5:95400831:95430864:HMEC;chr5:95401054:95431114:NHEK

ASHG19SELNC2A100010649	2.352	down	noncoding	ENST00000507997	noncoding	ENSG00000250551	RP11-254I22.1	Reliable	704	chr5:95385819-95551223:+	chr5:95449665:95456469:HepG2
ASHG19SELNC2A100010649	2.352	down	noncoding	ENST00000507997	noncoding	ENSG00000250551	RP11-254I22.1	Reliable	704	chr5:95385819-95551223:+	chr5:95533625:95596006:Osteoblasts;chr5:95554848:95596132:Skeletal Muscle Myoblast;chr5:95554848:95596194:HSMM;chr5:95554865:95565313:HMEC;chr5:95577923:95596155:IMR90

ASHG19SELNC2A100001112	3.6504	up	noncoding	ENST00000429328	noncoding	ENSG00000224805	LINC00853	661	chr1:47644922-47646011:+	chr1:47644197:47660664:K562;chr1:47644485:47659072:VACO 400;chr1:47645265:47648938:CD34 fetal

ASHG19SELNC2A100005356	2.3565	down	noncoding	ENST00000564381	noncoding	ENSG00000261045	RP11-673P17.4		Reliable	689		chr16:27169775-27187908:-	chr16:27180738:27219316:Toledo;chr16:27210289:27270958:Small Intestine;chr16:27210329:27271069:Sigmoid Colon;chr16:27211121:27269924:Gastric;chr16:27219367:27254473:Pancreas;chr16:27232475:27265537:NHDF-Ad;chr16:27232583:27270134:IMR90;chr16:27232897:27250352:Toledo;chr16:27233000:27271199:Lung;chr16:27233020:27270465:NHLF;chr16:27234684:27257134:DND41;chr16:27236435:27266186:CD8 primiary;chr16:27236550:27266388:CD4p CD25- CD45RAp Naive;chr16:27239895:27267778:CD4p CD25- Il17- PMAstim Th;chr16:27241177:27266265:CD56;chr16:27241349:27266178:CD4p CD25- Il17p PMAstim Th17;chr16:27241372:27250118:CD3;chr16:27241410:27250160:CD4 Naive Primary 8pool;chr16:27245404:27272047:HUVEC;chr16:27245453:27271543:Duodenum Smooth Muscle;chr16:27245721:27299114:u87;chr16:27245810:27298846:Osteoblasts;chr16:27278542:27286412:HepG2;chr16:27282675:27411338:CD14;chr16:27284252:27343050:Tonsil;chr16:27297077:27344019:Spleen;chr16:27323721:27343148:Sigmoid Colon;chr16:27323935:27342883:CD4p CD25- Il17p PMAstim Th17;chr16:27323967:27347443:IMR90;chr16:27324066:27389987:CD4p CD25- CD45RAp Naive;chr16:27324110:27347987:Duodenum Smooth Muscle;chr16:27324129:27347889:Osteoblasts;chr16:

ASHG19SELNC2A100006314	5.3044	down	noncoding	ENCT000003566 26	noncoding	CATG00000079483	CATG00000079483.1		2129	chr5:34584233-34586361:-	chr5:34562075:34612134:u87;chr5:34563079:34640959:Osteoblasts;chr5:34570192:34589139:Ly3;chr5:34570810:34629237:HUVEC;chr5:34585276:34610586:HCC1954
ASHG19SELNC2A100015859	2.1861	up	noncoding	ENST0000448272	noncoding	ENSG00000233256	RP11-445K13.2	Reliable	454	chr10:13457202-13465133:+	chr10:13432444:13460519:K562
ASHG19SELNC2A100006220	2.0547	up	noncoding	ENST0000608876	noncoding	ENSG00000273087	RP11-566J3.4	Reliable	640	chr14:101203922-101204561:+	chr14:100802621:100843836:CD14;chr14:100827954:100908202:Sigmoid Colon;chr14:100838663:100843727:K562;chr14:100838710:100875111:Small Intestine;chr14:100847356:100916786:Pancreatic islets;chr14:100849438:100914373:DND41;chr14:100849724:100908815:Colon Crypt 1;chr14:100850213:100908776:Colon Crypt 2;chr14:100871635:100914256:HeLa;chr14:100880989:101160433:H2171;chr14:100998206:101015206:Pancreas;chr14:101010832:101032668:Colon Crypt 2;chr14:101082708:101114187:DND41;chr14:101119022:101150896:Fetal Muscle;chr14:101124062:101147937:Fetal Intestine;chr14:101124290:101148211:VACO 400;chr14:101125048:101181157:Adrenal Gland;chr14:101130625:101147924:Ovary;chr14:101174098:101207742:Fetal Muscle

ASHG19SELNC2A100503198	2.3594	up	noncoding	ENST0000042709 3	noncoding	ENSG00000227695	DNMBP-AS1	474	chr10:101686767-101689595:+	chr10:101648092:101770791:Osteoblasts;chr10:101664796:101770704:HUVEC;chr10:101664881:101695667:HCT-116;chr10:101665653:101706057:NHEK;chr10:101668336:101692968:Colon Crypt 1;chr10:101671600:101679256:H1;chr10:101672994:101693123:Fetal Intestine;chr10:101673005:101693213:Fetal Intestine Large;chr10:101691338:101769690:NHDF-Ad;chr10:101720491:101781196:Panc1;chr10:101722368:101770605:Astrocytes;chr10:101722470:101769727:NHLF;chr10:101722958:101769501:NHEK;chr10:101722972:101745859:HMEC;chr10:101735144:101769681:Duodenum Smooth Muscle;chr10:101735315:101766481:Fetal Muscle;chr10:101735570:101786058:Fetal Thymus

ASHG19SELNC2A100000614	2.2073	down	noncoding	ENST0000052235 4	noncoding	ENSG00000254337	RP11-865I6.2		515	chr8:69760977-69764998:-	chr8:69762144:69787444:MCF-7
ASHG19SELNC2A100795225	2.2494	down	noncoding	ENST0000051939 3	noncoding	ENSG00000254319	RP11-134O21.1		564	chr8:2523614-2585929:-	chr8:2463918:2525201:Ly1
ASHG19SELNC2A100014662	2.8314	up	noncoding	ENST0000060961 1	noncoding	ENSG00000273141	RP11-820I16.4	Reliable	512	chr18:11908711-11909222:+	chr18:11880570:11920297:CD14

ASHG19SELNC2A100010804	2.0818	up	noncoding	ENST0000060273 9	noncoding	ENSG00000270020	RP11-463O9.9	Reliable	3515	chr16:86553989-86557503:-	chr16:86374860:86431236:u87;chr16:86376516:86431254:NHLF;chr16:86416810:86433203:IMR90;chr16:86428756:86497607:Stomach Smooth Muscle;chr16:86480878:86565786:Sigmoid Colon;chr16:86480959:86565581:Gastric;chr16:86504285:86554076:Lung;chr16:86510165:86565537:Esophagus;chr16:86510530:86552725:Stomach Smooth Muscle;chr16:86513308:86551280:Bladder;chr16:86524338:86565693:Small Intestine;chr16:86525013:86551948:Duodenum Smooth Muscle;chr16:86525585:86554162:Fetal Intestine Large;chr16:86526034:86552021:Fetal Intestine;chr16:86529885:86552655:IMR90;chr16:86530125:86553839:NHLF
ASHG19SELNC2A100013144	3.4772	up	noncoding	ENST0000061003 6	noncoding	ENSG00000272994	RP11-332H14.2	Reliable	3449	chr2:105950484-105953932:-	chr2:105913213:105957119:CD34 Primary RO01536;chr2:105936284:105948605:CD34 Primary RO01549;chr2:105939084:105957462:CD34 Primary RO01480
ASHG19SELNC2A100018416	4.7538	up	noncoding	ENST0000056516 2	noncoding	ENSG00000260924	AC004463.6	Reliable	1445	chr22:19158908-19160352:+	chr22:19156696:19168167:Colon Crypt 3;chr22:19157026:19167980:CD4 Memory Primary 7pool;chr22:19158029:19173513:VACO 503

ASHG19SELNC2A100263911	2.2433	down	noncoding	ENST0000033201 2	noncoding	ENSG00000185168	LINC00482		2023	chr17:79277571-79283048:-	chr17:79278020:79346912:Spleen;chr17:79289389:79371797:Colon Crypt 1;chr17:79289831:79323226:Sigmoid Colon;chr17:79289981:79323303:VACO 503;chr17:79290171:79403662:Esophagus;chr17:79300756:79321472:Right Atrium;chr17:79300771:79323073:Lung;chr17:79300804:79320863:Small Intestine;chr17:79300868:79339893:Fetal Intestine Large;chr17:79300913:79321146:Psoas Muscle;chr17:79301530:79306049:HCT-116;chr17:79301586:79321519:Panc1;chr17:79301633:79340371:Fetal Intestine;chr17:79301694:79319932:HeLa;chr17:79301741:79324408:VACO 400;chr17:79301790:79320769:VACO 9m;chr17:79301804:79324787:Fetal Muscle;chr17:79301805:79323383:LNCaP;chr17:79301842:79323135:Pancreatic islets;chr17:79302282:79323578:Gastric;chr17:79307665:79340673:Pancreas;chr17:79309282:79320792:Colon Crypt 2;chr17:79337759:79404370:Pancreatic islets;chr17:79337783:79409252:Gastric;chr17:79337835:79405362:HCT-116;chr17:79337884:79403652:Brain Hippocampus Middle;chr17:79337961:79404678:Lung;chr17:79338103:79377122:Brain Cingulate Gyrus;chr17:79338165:79371677:LNCaP;chr17:79338202:79375949:VACO 503;chr17:79338743:79379032:Brain Anterior
ASHG19SELNC2A100004696	2.0955	up	noncoding	ENST00000608515	noncoding	ENSG00000273055	CTB-13F3.1	Reliable	625	chr7:107582561-107583185:+	chr7:107530331:107645829:Adipose Nuclei;chr7:107572746:107613504:Osteoblasts;chr7:107573771:107614243:Duodenum Smooth Muscle;chr7:107599350:107651886:Sigmoid Colon;chr7:107600201:107644110:Fetal Muscle;chr7:107629021:107671709:HUVEC

ASHG19SELNC2A100004655	2.0576	up	noncoding	ENST000005915 01	noncoding	ENSG00000267100	ILF3-AS1	1983	chr19:10762538-10764520:-	chr19:10741990:10767090:Spleen;chr19:10752771:10767663:CD8 primiary;chr19:10753493:10767602:CD56

ASHG19SELNC2A100246467	2.0548	down	noncoding	ENST00000559292	noncoding	ENSG00000259448	RP11-16E12.1		1450	chr15:31508223-31512923:+	chr15:31488741:31529489:Ly3;chr15:31493248:31567464:Fetal Thymus;chr15:31494406:31509316:Thymus;chr15:31498214:31601710:H2171;chr15:31501187:31521891:Colon Crypt 3;chr15:31506380:31529829:Lung;chr15:31506686:31529709:Gastric;chr15:31506849:31521905:Colon Crypt 2;chr15:31540520:31560349:CD34 adult;chr15:31546224:31561885:MM1S;chr15:31546396:31565926:Sigmoid Colon;chr15:31546427:31566598:CD14;chr15:31546551:31566005:Colon Crypt 2;chr15:31547101:31560613:Ly3;chr15:31547210:31562077:Thymus;chr15:31547899:31559717:CD34 fetal;chr15:31553451:31691257:Brain Hippocampus Middle;chr15:31553552:31601202:NCI-H69;chr15:31554198:31565550:Spleen;chr15:31554706:31685021:Brain Cingulate Gyrus;chr15:31554850:31684654:Brain Anterior Caudate;chr15:31555127:31672882:Brain Inferior Temporal Lobe;chr15:31555415:31601468:Brain Hippocampus Middle 150;chr15:31558669:31601096:Brain Angular Gyrus;chr15:31588809:31660765:Fetal Thymus;chr15:31590716:31599435:CD34 fetal;chr15:31611693:31675292:CD14;chr15:31613604:31699171:CD20;chr15:31615375:31658617:Ly3;chr15:31615488:31685063:Brain Hippocampus Middle 150;chr15:31615508:31660953:Tonsil;chr15:316156
ASHG19SELNC2A100004612	2.087	up	noncoding	ENST00000558702	noncoding	ENSG00000259215	RP11-253M7.4	Reliable	391	chr15:69750861-69754145:-	chr15:69735057:69762697:CD4 Memory Primary 7pool;chr15:69754181:69778076:LNCaP

ASHG19SELNC2A100017012	2.7933	down	noncoding	ENST00000604485	noncoding	ENSG00000271216	LINC01050		760	chr13:43384502-43386698:-	chr13:43377524:43424659:u87
ASHG19SELNC2A100016218	2.2835	up	noncoding	ENST00000609434	noncoding	ENSG00000272791	RP11-464F9.22	Reliable	935	chr10:75390314-75391248:+	chr10:75384396:75417277:Fetal Muscle;chr10:75384653:75417403:Skeletal Muscle;chr10:75384884:75416695:Psoas Muscle;chr10:75403720:75417344:Left Ventricle;chr10:75403902:75416581:Right Atrium
ASHG19SELNC2A100542690	3.4093	down	noncoding	ENST00000481898	noncoding	ENSG00000175061	FAM211A-AS1		993	chr17:16342301-16345342:+	chr17:16341332:16348323:HepG2;chr17:16344475:16393335:Spleen;chr17:16345196:16424854:Fetal Intestine Large;chr17:16345354:16395538:Small Intestine;chr17:16346062:16393620:Adrenal Gland;chr17:16346673:16395506:Colon Crypt 1;chr17:16348624:16395296:Fetal Intestine;chr17:16363328:16370398:Colon Crypt 2;chr17:16365702:16395445:Sigmoid Colon

ASHG19SELNC2A100288393	2.1325	down	noncoding	ENST00000509036	noncoding	ENSG00000251131	CTD-2035E11.3	Gold	472	chr5:43018531-43024349:+	chr5:42974744:43010144:Astrocytes;chr5:42982065:43021566:CD4 Memory Primary 7pool;chr5:42982072:43021311:CD34 Primary RO01536;chr5:42982188:43021349:CD8 Memory 7pool;chr5:42982625:43009005:CD34 adult;chr5:42982779:43008925:CD34 fetal;chr5:42984005:43021232:HeLa;chr5:42984195:43020926:Esophagus;chr5:42984237:43021003:Osteoblasts;chr5:42984869:43020974:NHLF;chr5:42985069:43010090:VACO 9m;chr5:42987481:42997335:CD8 primiary;chr5:42988207:43021793:CD56;chr5:42988303:42997081:CD4p CD25- Il17- PMAstim Th;chr5:42988340:43022194:CD4p CD25- CD45ROp Memory;chr5:42988541:42996957:CD4p CD25- CD45RAp Naive;chr5:42988679:43021490:CD4 Naive Primary 8pool;chr5:42988731:43021440:CD8 Naive 8pool;chr5:42988739:43021396:CD8 Naive 7pool;chr5:42988783:43021518:CD3;chr5:42988888:43021861:CD4p CD25- Il17p PMAstim Th17;chr5:42988915:43022042:Tonsil;chr5:42988940:43021536:CD4p CD225int CD127p Tmem;chr5:42989202:42996790:HUVEC;chr5:42989365:42996520:CD20;chr5:42989548:43021294:CD4 Naive Primary 7pool;chr5:42989568:43021469:CD4 Memory Primary 8pool;chr5:42989815:43020989:Spleen;chr5:42989931:43020950:Sigmoid Colon;chr5:42989941:43020943:Small

ASHG19SELNC2A100016153	5.5613	up	noncoding	ENST000005711 97	noncoding	ENSG00000262160	RP11-96D1.11		3177	chr16:68259872-68263048:-	chr16:68212700:68272196:Thymus;chr16:68249856:68272392:Gastric;chr16:68261418:68272282:VACO 400;chr16:68261815:68274246:Colon Crypt 2;chr16:68262082:68272197:LNCaP;chr16:68265378:68274180:Colon Crypt 3;chr16:68266153:68272416:Esophagus;chr16:68267165:68272241:VACO 9m;chr16:68267481:68272278:Pancreas;chr16:68270515:68333501:Right Atrium;chr16:68270633:68335789:Adipose Nuclei;chr16:68270826:68307258:Adrenal Gland;chr16:68271675:68292320:Right Ventricle;chr16:68277977:68320878:MM1S;chr16:68278112:68323969:DHL6;chr16:68285503:68335472:CD20;chr16:68285736:68332888:Thymus;chr16:68285778:68325411:Tonsil;chr16:68291070:68305604:HepG2;chr16:68291072:68324941:Sigmoid Colon;chr16:68291183:68318135:Colon Crypt 2;chr16:68296590:68326585:CD4p CD25- CD45ROp Memory;chr16:68296946:68326806:CD8 primiary;chr16:68302759:68326322:CD3
ASHG19SELNC2A100016228	4.6678	up	noncoding	ENST000006101 85	noncoding	ENSG00000273355	RP11-672L10.6	Reliable	483	chr18:813275-813757:+	chr18:804720:842932:CD4 Memory Primary 7pool;chr18:804957:817093:CD8 Memory 7pool;chr18:807287:814934:CD4p CD25- Il17- PMAstim Th;chr18:807552:842678:K562

ASHG19SELNC2A100014588	2.4044	down	noncoding	ENST0000057026 9	noncoding	ENSG00000259976	RP11-553L6.5		1679	chr3:114033348-114035026:-	chr3:114003952:114058432:CD8 primiary;chr3:114003955:114033752:CD4 Naive Primary 8pool;chr3:114003976:114054019:CD4p CD25- Il17- PMAstim Th;chr3:114004013:114058398:Tonsil;chr3:114004021:114033604:CD8 Memory 7pool;chr3:114004022:114028685:CD56;chr3:114004096:114058306:CD3
ASHG19SELNC2A100013735	3.1615	up	noncoding	ENST00000510419	noncoding	ENSG00000249942	AC142293.3	Reliable	732	chr4:75418301-75514664:-	chr4:75452158:75493257:HMEC;chr4:75452163:75492984:NHEK;chr4:75480189:75492733:VACO 400

ASHG19SELNC2A100573558	2.5192	up	noncoding	MICT000002712 16	noncoding	CATG00000070175	CATG00000070175.1		7096	chr4:129574878-129588472:+	chr4:129578348:129598633:MM1S
ASHG19SELNC2A100015370	2.5393	up	noncoding	ENST00000578226	noncoding	ENSG00000265342	RP11-16C1.1	Reliable	372	chr17:73390204-73391044:+	chr17:73284104:73345918:HBL1;chr17:73287335:73369120:Tonsil;chr17:73287754:73415375:CD14;chr17:73289760:73295202:CD19 Primary;chr17:73289805:73357070:Spleen;chr17:73305109:73364292:CD34 Primary RO01536;chr17:73307734:73368344:CD19 Primary;chr17:73317653:73362184:DND41;chr17:73321133:73403212:CD4p CD25- Il17- PMAstim Th;chr17:73324621:73349939:CD20;chr17:73349943:73394321:CD20
ASHG19SELNC2A100006113	2.2665	up	noncoding	ENST00000562538	noncoding	ENSG00000260246	AC000032.2	Reliable	491	chr1:110235739-110236364:-	chr1:110221396:110276610:HCC1954

ASHG19SELNC2A100009987	2.7714	up	noncoding	ENST00000568560	noncoding	ENSG00000260755	RP11-403P17.3	Reliable	511	chr16:66543340-66543951:+	chr16:66535918:66584785:Adipose Nuclei;chr16:66536008:66554759:Aorta;chr16:66536124:66560302:Lung;chr16:66536193:66582859:Right Atrium;chr16:66536246:66559860:Esophagus;chr16:66536953:66561850:CD14;chr16:66542160:66560665:Brain Hippocampus Middle;chr16:66542400:66552090:LNCaP;chr16:66542514:66584999:Pancreatic islets;chr16:66542534:66552236:Pancreas;chr16:66542716:66558847:Colon Crypt 2;chr16:66544127:66561658:Spleen;chr16:66544583:66558358:HUVEC;chr16:66544758:66556721:VACO 9m;chr16:66544956:66582850:Small Intestine;chr16:66548929:66588057:MM1S;chr16:66549025:66560784:CD56;chr16:66549058:66560556:Sigmoid Colon;chr16:66549099:66595846:DND41;chr16:66550328:66559743:Adipose Tissue;chr16:66582086:66598370:Jurkat
ASHG19SELNC2A100008010	2.2047	up	noncoding	ENST00000487567	noncoding	ENSG00000223776	LGALS8-AS1		1440	chr1:236686369-236687808:-	chr1:236634110:236713915:Panc1;chr1:236661774:236694741:HCC1954

ASHG19SELNC2A100015202	2.005	up	noncoding	ENST00000510234	noncoding	ENSG00000249306	RP11-267A15.1	844	chr5:173763498-173954048:+	chr5:173908514:173960764:NCI-H69;chr5:173928928:173962818:VACO 503

ASHG19SELNC2A100004368	2.0074	up	noncoding	ENST00000453579	noncoding	ENSG00000232529	RP1-257I9.2	Reliable	554	chr6:155701645-155702317:-	chr6:155695399:155722912:u87
ASHG19SELNC2A100828506	2.2218	down	noncoding	ENST00000438195	noncoding	ENSG00000230812	RP4-794H19.4		306	chr1:59486260-59510286:+	chr1:59473624:59529508:u87
ASHG19SELNC2A101049578	3.3092	down	noncoding	uc002qyd.1	noncoding	ENSG00000188765	TMSB4XP2	Reliable	405	chr2:3664890-3665294:-	chr2:3651194:3704067:Ovary;chr2:3689523:3716270:HBL1;chr2:3697967:3716182:Ly3

ASHG19SELNC2A100024822	3.0534	down	noncoding	MICT0000369286	noncoding	CATG00000110132	CATG00000110132.1	9706	chr9:139454744-139482374:-	chr9:139375697:139380563:HeLa;chr9:139377671:139492039:Pancreatic islets;chr9:139377687:139467997:Lung;chr9:139384093:139525246:Colon Crypt 3;chr9:139387062:139501507:Brain Hippocampus Middle;chr9:139387064:139447553:Brain Cingulate Gyrus;chr9:139387407:139459693:Brain Hippocampus Middle 150;chr9:139387413:139447503:Brain Anterior Caudate;chr9:139402936:139468095:Right Atrium;chr9:139403716:139442661:Left Ventricle;chr9:139404221:139463290:Adipose Nuclei;chr9:139404817:139464701:Sigmoid Colon;chr9:139404844:139460044:Small Intestine;chr9:139404943:139463564:Esophagus;chr9:139405020:139447486:Brain Inferior Temporal Lobe;chr9:139405028:139461453:Fetal Muscle;chr9:139405239:139438180:Brain Angular Gyrus;chr9:139407512:139445889:Colon Crypt 2;chr9:139412822:139442518:Colon Crypt 1;chr9:139412956:139460358:CD14;chr9:139413605:139438320:VACO 400;chr9:139420117:139446243:HCT-116;chr9:139420323:139471373:Stomach Smooth Muscle;chr9:139420329:139463202:Gastric;chr9:139420405:139453240:Pancreas;chr9:139420496:139446026:VACO 503;chr9:139420602:139461721:Adrenal Gland;chr9:139422133:139445956:LNCaP;chr9:139423110:139447563:Tonsil;chr9:139430279:139442814:Spleen;chr9:139470097:139525295:H2171;chr9:13

ASHG19SELNC2A100003179	4.2974	up	noncoding	ENST00000607241	noncoding	ENSG00000271855	RP11-214N9.1	Reliable	877	chr2:9696028-9696904:+	chr2:9677223:9706542:HMEC;chr2:9678117:9697760:DHL6;chr2:9691786:9706213:NHEK
ASHG19SELNC2A100562079	2.7566	down	noncoding	ENST00000532002	noncoding	ENSG00000247416	RP11-629G13.1		566	chr11:112831050-112832021:-	chr11:112801787:112849540:HSMMtube

ASHG19SELNC2A100017623	2.5086	up	noncoding	ENST0000560769	noncoding	ENSG00000259279	CTD-2033D15.1	Reliable	281	chr15:39885781-39886432:-	chr15:39865186:39885963:NHDF-Ad;chr15:39865313:39892941:Osteoblasts;chr15:39869915:39877299:IMR90;chr15:39869915:39888876:HUVEC;chr15:39870628:39888468:HSMM;chr15:39870701:39888468:Skeletal Muscle Myoblast;chr15:39870728:39934231:Sigmoid Colon;chr15:39900509:39924276:CD56;chr15:39908178:39935465:NHEK;chr15:39913693:39922988:CD3;chr15:39914552:39937162:CD4p CD25- Il17p PMAstim Th17
ASHG19SELNC2A100011591	2.7196	up	noncoding	ENST0000607122	noncoding	ENSG00000272444	RP11-1017G21.6	Reliable	648	chr14:102418753-102419400:+	chr14:102413635:102440255:VACO503;chr14:102413638:102448327:NHDF-Ad;chr14:102413684:102439387:Colon Crypt 3

ASHG19SELNC2A100013654	2.1714	down	noncoding	ENST00000471719	noncoding	ENSG00000241135	LINC00881	791	chr3:156807670-156818924:+	chr3:156780833:156808483:Brain Hippocampus Middle 150;chr3:156781155:156808652:Brain Anterior Caudate;chr3:156781701:156822304:Brain Cingulate Gyrus;chr3:156781703:156809508:Brain Hippocampus Middle;chr3:156782059:156809879:Brain Inferior Temporal Lobe;chr3:156783670:156817525:Osteoblasts;chr3:156784215:156808538:Brain Angular Gyrus;chr3:156794674:156855343:Right Atrium;chr3:156795252:156825935:MM1S;chr3:156798403:156807714:HMEC;chr3:156798626:156853389:CD4 Memory Primary 7pool;chr3:156798763:156808423:NHEK;chr3:156802888:156855556:Left Ventricle;chr3:156802993:156855459:Lung;chr3:156803058:156831056:Right Ventricle;chr3:156803413:156809144:Fetal Muscle;chr3:156804380:156826978:Ly4;chr3:156804477:156811628:Fetal Intestine Large;chr3:156804608:156808840:Fetal Intestine;chr3:156804660:156822268:Small Intestine;chr3:156804954:156823499:Sigmoid Colon;chr3:156832630:156855726:Fetal Intestine Large;chr3:156832745:156855695:Fetal Intestine;chr3:156833836:156855198:VACO 503;chr3:156836974:156855523:Sigmoid Colon;chr3:156837068:156855821:HepG2;chr3:156837190:156855546:Small Intestine;chr3:156837212:156855183:Colon Crypt

ASHG19SELNC2A100012357	2.3729	up	noncoding	ENST00000597592	noncoding	ENSG00000269350	CTD-2278I10.1	Reliable	661	chr19:17471741-17473562:+	chr19:17457576:17524836:Spleen
ASHG19SELNC2A100200307	2.4072	down	noncoding	ENST00000599946	noncoding	ENSG00000260496	RP11-161M6.3		984	chr16:1041148-1050926:-	chr16:987327:1043016:Brain Hippocampus Middle;chr16:987380:1042968:Brain Cingulate Gyrus;chr16:987581:1042744:Brain Hippocampus Middle 150;chr16:1001178:1042456:Brain Anterior Caudate;chr16:1013186:1042763:Brain Inferior Temporal Lobe;chr16:1014623:1041623:Brain Angular Gyrus;chr16:1022902:1041491:Brain Mid Frontal Lobe
ASHG19SELNC2A100004004	2.4729	up	noncoding	ENST00000609194	noncoding	ENSG00000272931	RP5-943J3.2	Reliable	695	chr1:90285733-90286427:-	chr1:90277508:90410092:CD14;chr1:90284891:90322643:CD4 Memory Primary 7pool;chr1:90285072:90310876:Brain Hippocampus Middle 150;chr1:90285126:90311060:Brain Inferior Temporal Lobe;chr1:90285395:90314172:Brain Cingulate Gyrus;chr1:90307860:90337707:Tonsil;chr1:90307980:90378248:Osteoblasts;chr1:90335992:90376985:CD4 Memory Primary 7pool;chr1:90338335:90385797:CD34 Primary RO01536;chr1:90342913:90374716:CD8 Memory 7pool;chr1:90343713:90375012:K562;chr1:90348830:90376992:HUVEC;chr1:90358848:90419578:HSM Mtube;chr1:90362136:90372507:CD34 fetal;chr1:90369156:90423654:Brain Anterior Caudate;chr1:90371516:90379707:H1

ASHG19SELNC2A100007725	2.9474	down	noncoding	ENST0000062 17	noncoding	ENSG00000272121	RP4-555D20.4	Reliable	1069	chr3:44039573-44040641:-	chr3:44036029:44064886:u87
ASHG19SELNC2A101016732	2.1241	up	noncoding	ENST0000439192	noncoding	ENSG00000237883	DGUOK-AS1		563	chr2:74185097-74208560:-	chr2:74152837:74230505:CD4p CD25- Il17- PMAstim Th;chr2:74190932:74215041:Gastric;chr2:74195960:74284592:CD14;chr2:74197681:74238940:Esophagus;chr2:74197864:74215272:Colon Crypt 3;chr2:74197915:74227390:Colon Crypt 2;chr2:74210450:74213656:VACO 400;chr2:74210530:74213466:VACO 9m;chr2:74243858:74266802:Ly3;chr2:74252781:74264602:CD34 adult;chr2:74253093:74263447:CD34 fetal

ASHG19SELNC2A100001560	2.2907	up	noncoding	ENST00000599382	noncoding	ENSG00000267838	AC008746.12	Reliable	516	chr19:54949847-54950362:-	chr19:54921528:54950915:Brain Inferior Temporal Lobe;chr19:54925536:54963097:Brain Hippocampus Middle;chr19:54925733:54963082:Brain Cingulate Gyrus;chr19:54925765:54950647:Brain Hippocampus Middle 150;chr19:54926568:54951103:Brain Anterior Caudate;chr19:54926765:54947982:Brain Angular Gyrus;chr19:54929083:54947767:Brain Mid Frontal Lobe;chr19:54929167:54983116:Pancreas;chr19:54929167:54983278:Pancreatic islets;chr19:54962054:54983242:Gastric;chr19:54962064:54983154:Colon Crypt 1;chr19:54962449:54981338:LNCaP;chr19:54970662:54983351:Small Intestine;chr19:54970705:54983132:Spleen;chr19:54970768:54983186:Sigmoid Colon;chr19:54971335:54983117:Colon Crypt 2;chr19:54971393:54983161:Colon Crypt 3;chr19:54972271:54983227:Fetal Intestine;chr19:54972299:54983257:Fetal Intestine Large
ASHG19SELNC2A101029045	2.4916	up	noncoding	ENST00000605437	noncoding	ENSG00000270820	RP11-355B11.2		569	chr2:61710508-61711076:+	chr2:61645792:61699770:DHL6;chr2:61684686:61721814:CD34 Primary RO01536

ASHG19SELNC2A100008485	2.2805	up	noncoding	ENST00000607710	noncoding	ENSG00000272384	RP11-44N11.2	Reliable	860	chr8:123792210-123793069:-	chr8:123784378:123822569:Ly1;chr8:123784540:123802897:Toledo;chr8:123791374:123827463:CD20;chr8:123791599:123812517:CD4p CD25- CD45ROp Memory;chr8:123791640:123810189:Brain Hippocampus Middle;chr8:123791657:123796406:CD56;chr8:123791698:123811809:Tonsil;chr8:123791740:123813147:CD4p CD25- CD45RAp Naive;chr8:123791830:123872108:Brain Cingulate Gyrus;chr8:123791831:123812706:CD8 Naive 8pool;chr8:123791885:123796448:CD8 Memory 7pool;chr8:123791897:123822383:CD19 Primary;chr8:123791903:123812493:CD8 primiary;chr8:123791925:123872233:Brain Hippocampus Middle 150;chr8:123791951:123877195:Left Ventricle;chr8:123791991:123876684:Psoas Muscle;chr8:123792006:123816260:Brain Inferior Temporal Lobe;chr8:123792022:123796317:CD4p CD225int CD127p Tmem;chr8:123792030:123796609:CD4p CD25- Il17p PMAstim Th17;chr8:123792055:123812548:CD4 Naive Primary 8pool;chr8:123792091:123796339:CD4 Memory Primary 8pool;chr8:123792116:123796311:CD4 Naive Primary 7pool;chr8:123792116:123796424:CD8 Naive 7pool;chr8:123792157:123842563:Esophagus;chr8:123792221:123796154:CD3;chr8:123792234:1238754

ASHG19SELNC2A100016948	2.2435	down	noncoding	ENST000005682 44	noncoding	ENSG00000261730	RP4-668J24.2	Reliable	1277	chr6:1384025-1385301:-	chr6:1381610:1408672:Esophagus;chr6:1381779:1419324:Sigmoid Colon;chr6:1381898:1397393:Stomach Smooth Muscle;chr6:1382269:1397250:NHLF

ASHG19SELNC2A100000280	4.1779	up	noncoding	ENST00000441932	noncoding	ENSG00000223396	RPS10P7	2329	chr1:201487831-201499602:+	chr1:201494568:201532965:u87;chr1:201498845:201533209:NHDF-Ad;chr1:201499023:201579537:HSMMtube;chr1:201499254:201569312:Brain Inferior Temporal Lobe;chr1:201499296:201578800:Osteoblasts;chr1:201499466:201554633:Brain Hippocampus Middle;chr1:201499539:201554513:Brain Cingulate Gyrus;chr1:201499826:201533160:Skeletal Muscle Myoblast;chr1:201499826:201533164:HSMM;chr1:201499829:201533172:NHLF;chr1:201499897:201554112:Brain Hippocampus Middle 150;chr1:201499897:201584056:Pancreatic islets;chr1:201499964:201540850:Brain Angular Gyrus;chr1:201500984:201535523:Brain Mid Frontal Lobe;chr1:201505191:201533031:Astrocytes;chr1:201506512:201554222:Brain Anterior Caudate
ASHG19SELNC2A100001633	7.3162	down	noncoding	ENCT00000191243	noncoding	CATG00000035279	CATG00000035279.1	1077	chr18:20046289-20047365:+	chr18:20028365:20084909:Right Atrium;chr18:20045383:20051165:NHEK

ASHG19SELNC2A100013815	3.9454	up	noncoding	ENST00000554679	noncoding	ENSG00000258837	CTD-2566J3.1	Reliable	537	chr14:68591721-68596913:-	chr14:68554737:68614521:Fetal Muscle;chr14:68577448:68642169:Fetal Intestine Large;chr14:68590700:68614976:Osteoblasts;chr14:68591170:68615328:HSMMtube;chr14:68591341:68737027:Stomach Smooth Muscle;chr14:68591365:68615072:HSMM;chr14:68591472:68614275:Duodenum Smooth Muscle;chr14:68591506:68615055:Skeletal Muscle Myoblast;chr14:68591560:68625251:u87;chr14:68591577:68625555:NHDF-Ad;chr14:68591622:68625859:NHEK;chr14:68591661:68625836:HMEC;chr14:68603422:68723615:HCC1954;chr14:68657764:68719730:Duodenum Smooth Muscle;chr14:68657799:68776455:Fetal Muscle;chr14:68665359:68776268:Fetal Thymus;chr14:68696698:68731840:Fetal Intestine;chr14:68696948:68718223:HSMMtube;chr14:68697493:68731429:Fetal Intestine Large;chr14:68697803:68809598:Tonsil;chr14:68713947:68800771:HBL1;chr14:68714257:68809954:CD20;chr14:68714552:68809686:GM12878;chr14:68714591:68775012:Ly1;chr14:68714763:68780763:Ly4;chr14:68715210:68761544:CD19 Primary;chr14:68716778:68753049:Ly3;chr14:68732305:68761095:DHL6;chr14:68732586:68762984:Toledo;chr14:68732676:68760612:CD56;chr14:68745751:68777072:VACO 400;chr14:68793306:68884369:Fetal Muscle;chr14:68813249:68847179:VACO 400;chr14:68815660:68880386:Fetal

ASHG19SELNC2A100009129	6.3739	down	noncoding	ENST00000550290	noncoding	ENSG00000257354	RP11-631N16.2	Reliable	555	chr12:62996532-63007855:+	chr12:62994321:62999146:Toledo;chr12:62994460:62999610:CD20;chr12:62995209:62999729:CD4p CD25- CD45ROp Memory;chr12:62995321:62999251:Fetal Muscle;chr12:62995364:62999232:CD56
ASHG19SELNC2A100463180	5.5005	down	noncoding	ENST00000412571	noncoding	ENSG00000233895	RP1-122P22.2		415	chr20:19738792-19789926:+	chr20:19680519:19716860:Ly4;chr20:19713677:19767915:H2171;chr20:19715694:19742104:Esophagus;chr20:19717468:19740094:Colon Crypt 3;chr20:19737239:19768983:HSMMtube;chr20:19737269:19809215:Left Ventricle;chr20:19737283:19767891:Lung;chr20:19782303:19821568:HUVEC

ASHG19SELNC2A100010820	3.4083	up	noncoding	ENST00000608142	noncoding	ENSG00000273445	RP11-1399P15.1	Reliable	540	chr2:87777014-87777553:-	chr2:87741930:87850250:CD4p CD25- Il17-PMAstim Th;chr2:87741939:87838628:CD8 primiary;chr2:87741950:87835062:Sigmoid Colon;chr2:87742030:87838478:CD56;chr2:87742086:87799484:MM1S;chr2:87742118:87849039:CD3;chr2:87742123:87839551:Small Intestine;chr2:87742227:87849883:CD4p CD25-Il17p PMAstim Th17;chr2:87742275:87840521:Spleen;chr2:87744062:87770117:u87;chr2:87744157:87814392:HSMMtube;chr2:87744465:87770062:HSMM;chr2:87744472:87812291:IMR90;chr2:87744542:87769924:Skeletal Muscle Myoblast;chr2:87744557:87803367:HUVEC;chr2:87744763:87759323:HMEC;chr2:87744766:87860202:Osteoblasts;chr2:87744785:87808232:NHLF;chr2:87744801:87809293:Astrocytes;chr2:87744806:87797783:HeLa;chr2:87744891:87840736:CD14;chr2:87744961:87767255:NHEK;chr2:87748585:87770601:NHDF-Ad;chr2:87750583:87769736:Aorta;chr2:87750709:87815611:Panc1;chr2:87751176:87822848:Duodenum Smooth Muscle;chr2:87751194:87819799:Adipose Nuclei;chr2:87751406:87769489:Fetal Muscle;chr2:87751432:87798788:Lung;chr2:87751559:87835511:Skeletal Muscle;chr2:87751577:87798947:HCC1954;chr2:87752153:87850421:CD4 Memory Primary 7pool;chr2:87752715:87844316:CD34 Primary RO01536;chr2:87752926:87769391:CD34 Primary

ASHG19SELNC2A100013006	2.2141	up	noncoding	ENST000005293 69	noncodi ng	ENSG000002 54682	RP11-660L16.2	Gold	680	chr11:71159720-71163203:+	chr11:71101232:71132644:Jurkat;chr11:71128818:71177079:Brain Cingulate Gyrus;chr11:71143042:71176809:Brain Hippocampus Middle

ASHG19SELNC2A100008449	3.5274	up	noncoding	ENST00000583409	noncoding	ENSG00000266040	CTB-58E17.3	Reliable	486	chr17:36859733-36862514:-	chr17:36841483:36864135:Spleen;chr17:36849347:36863638:Left Ventricle;chr17:36849416:36867442:Fetal Muscle;chr17:36849427:36868217:Lung;chr17:36849443:36863581:Small Intestine;chr17:36849446:36869721:Right Atrium;chr17:36849486:36864348:Sigmoid Colon;chr17:36849658:36862895:Right Ventricle;chr17:36849707:36864129:Gastric;chr17:36852020:36863435:MM1S;chr17:36852687:36863936:Tonsil;chr17:36854835:36891315:CD4p CD25- Il17p PMAstim Th17;chr17:36854933:36891548:CD4p CD25- Il17- PMAstim Th;chr17:36856374:36863732:Ly3;chr17:36856433:36863910:CD56;chr17:36856888:36888783:CD8 primiary;chr17:36857029:36864323:Pancreatic islets;chr17:36857157:36863577:Esophagus;chr17:36857165:36863536:Adrenal Gland;chr17:36857173:36861522:K562;chr17:36857186:36861774:HeLa;chr17:36857283:36862851:Pancreas;chr17:36857283:36862900:VACO 9m;chr17:36857336:36862962:Thymus;chr17:36857431:36876540:VACO 503;chr17:36880595:36907474:Gastric;chr17:36880935:36900163:Colon Crypt 2;chr17:36881475:36907782:Small Intestine;chr17:36881476:36907973:Colon Crypt 1;chr17:36883977:36907951:Lung;chr17:36886485:36906657:Pancreas;chr17:36886615:36890751:LNCaP
ASHG19SELNC2A100016225	2.1807	up	noncoding	ENST00000608397	noncoding	ENSG00000273407	RP11-757A13.1	Reliable	350	chr7:99364166-99364515:+	chr7:99317323:99393672:Fetal Intestine Large;chr7:99317435:99393579:Fetal Intestine

ASHG19SELNC2A100016997	2.6999	up	noncoding	ENST0000059795 3	noncoding	ENSG00000268129	RP11-91G21.1	561	chr3:142719749-142720309:-	chr3:142716077:142722234:K562
ASHG19SELNC2A100009252	2.324	down	noncoding	ENST00000610103	noncoding	ENSG00000272761	RP11-572C15.6	5109	chr3:112315641-112320749:-	chr3:112317092:112416252:Osteoblasts;chr3:112323193:112377100:u87;chr3:112344841:112374149:Adipose Nuclei;chr3:112346174:112374310:Duodenum Smooth Muscle;chr3:112347706:112375700:HSMMtube;chr3:112350638:112375251:NHDF-Ad;chr3:112353228:112374918:IMR90;chr3:112353537:112375297:NHLF;chr3:112353786:112375136:Astrocytes;chr3:112354527:112375302:HSMM;chr3:112356164:112375299:Skeletal Muscle Myoblast

ASHG19SELNC2A100007599	2.6094	down	noncoding	ENST00000548322	noncoding	ENSG00000257285	RP11-298I3.1	591	chr14:23398854-23424771:+	chr14:23384926:23403521:CD20;chr14:23385986:23390185:CD4 Memory Primary 7pool
ASHG19SELNC2A100296239	2.2403	down	noncoding	MICT00000062622	noncoding	CATG00000006004	CATG00000006004.1	1636	chr11:69040085-69042067:-	chr11:69019019:69070837:HCC1954;chr11:69035833:69089097:Pancreatic islets;chr11:69040170:69070245:Esophagus;chr11:69040207:69079860:Gastric;chr11:69040227:69070579:Colon Crypt 1;chr11:69040530:69066696:Pancreas;chr11:69040623:69070143:Colon Crypt 2;chr11:69041149:69070238:VACO 400;chr11:69041330:69070368:Sigmoid Colon;chr11:69041617:69072027:VACO 503;chr11:69043247:69081064:Ly4;chr11:69048050:69089929:u87;chr11:69048698:69070633:VACO 9m;chr11:69059646:69088726:Panc1;chr11:69059793:69071009:HCT-116;chr11:69059907:69089985:HUVEC;chr11:69061501:69070316:K562;chr11:69063032:69089949:Osteoblasts;chr11:69063158:69072297:HeLa;chr11:69063267:69089937:NHLF;chr11:69063296:69089921:NHDF-Ad;chr11:69063593:69089932:HSMM;chr11:69063607:69089917:Skeletal Muscle Myoblast;chr11:69063939:69070881:Astrocytes

ASHG19SELNC2A100735568	3.5021	up	noncoding	ENST00000432368	noncoding	ENSG00000228791	THRB-AS1	1216	chr3:24535578-24541833:+	chr3:24522776:24567093:Fetal Intestine Large;chr3:24522920:24548330:Fetal Intestine
ASHG19SELNC2A100007046	2.3699	up	noncoding	MICT00000291141	noncoding	CATG00000078011	CATG00000078011.1	1083	chr5:148505082-148509583:+	chr5:148494011:148524070:VACO 503;chr5:148509370:148604667:Right Atrium;chr5:148509613:148609765:Left Ventricle;chr5:148512508:148556019:u87;chr5:148512944:148610042:Adipose Nuclei;chr5:148513084:148566045:HSMMtube;chr5:148513364:148558772:Lung;chr5:148578617:148616086:HSMMtube;chr5:148582893:148601148:NHLF;chr5:148583745:148601508:IMR90

ASHG19SELNC2A101601719	2.5271	down	noncoding	ENST00000505701	noncoding	ENSG00000249899	CTD-2175A23.1	595	chr5:52228249-52286108:-	chr5:52278686:52341416:u87;chr5:52279203:52341614:HUVEC;chr5:52280156:52305573:NHEK;chr5:52280307:52321022:Ly3;chr5:52281822:52335162:HMEC;chr5:52283967:52328432:Brain Hippocampus Middle 150;chr5:52284029:52334970:Brain Cingulate Gyrus;chr5:52318977:52336406:NHEK

ASHG19SELNC2A100005228	2.7752	down	noncoding	ENST000005005 90	noncoding	ENSG00000245261	RP3-330M21.5	Reliable	980	chr6:43181539-43191598:-	chr6:43188593:43194873:CD20;chr6:43190623:43194520:CD19 Primary
ASHG19SELNC2A100016778	7.1284	down	noncoding	ENST0000046823 2	noncoding	ENSG00000242578	RP11-469J4.3		664	chr3:170128300-170136403:+	chr3:170131702:170167659:Brain Cingulate Gyrus;chr3:170135437:170167310:Brain Hippocampus Middle;chr3:170135527:170167574:Brain Inferior Temporal Lobe;chr3:170135615:170167650:Brain Hippocampus Middle 150;chr3:170135678:170167261:Brain Anterior Caudate;chr3:170140567:170167029:Brain Angular Gyrus

ASHG19SELNC2A100002836	2.4313	up	noncoding	ENST00000445833	noncoding	ENSG00000232290	RP1-292B18.3	Reliable	396	chr6:151376033-151377193:-	chr6:151323859:151393377:HUVEC;chr6:151324807:151397937:HSMMtube;chr6:151325157:151418342:HCT-116;chr6:151325315:151393091:Astrocytes;chr6:151354393:151393793:Osteoblasts;chr6:151356764:151392804:u87;chr6:151356767:151403524:HMEC;chr6:151361391:151393112:Panc1;chr6:151380439:151397528:Skeletal Muscle Myoblast;chr6:151380439:151397540:HSMM
ASHG19SELNC2A100003622	5.754	up	noncoding	ENST00000440397	noncoding	ENSG00000230533	RP11-95M15.1	Gold	523	chr6:137994597-137995691:+	chr6:137957798:138068513:DND41;chr6:137999688:138039938:CD4p CD25- Il17p PMAstim Th17;chr6:138018230:138068482:Fetal Thymus;chr6:138019585:138066867:Jurkat;chr6:138019709:138071429:u87;chr6:138027743:138039791:CD4p CD225int CD127p Tmem;chr6:138027835:138039806:CD3;chr6:138027981:138039926:CD4 Naive Primary 8pool

ASHG19SELNC2A100006863	3.5105	up	noncoding	ENST0000540392	noncoding	ENSG00000256234	RP11-283G6.4	699	chr12:26364097-26488789:-	chr12:26423624:26453350:u87;chr12:26423782:26453012:HMEC
ASHG19SELNC2A100012058	5.5009	up	noncoding	ENST0000414452	noncoding	ENSG00000232480	RP11-224O19.2	557	chr1:218517538-218519020:-	chr1:218508854:218559528:HSMMtube;chr1:218509141:218577202:Fetal Muscle;chr1:218517509:218589915:Skeletal Muscle;chr1:218517549:218576410:Panc1;chr1:218517751:218613713:Osteoblasts

ASHG19SELNC2A100564182	3.4971	down	noncoding	ENST00000524045	noncoding	ENSG00000251003	RP11-152P17.2	987	chr8:106797033-107072705:-	chr8:107056599:107074501:u87;chr8:107057040:107074578:Astrocytes

ASHG19SELNC2A100795040	2.0923	down	noncoding	ENST00000544868	noncoding	ENSG00000251562	MALAT1	4585	chr11:65266516-65271100:+	chr11:65236062:65284689:Brain Anterior Caudate;chr11:65236141:65278601:Sigmoid Colon;chr11:65236162:65278849:HeLa;chr11:65236264:65284293:Brain Angular Gyrus;chr11:65236275:65278454:Psoas Muscle;chr11:65236293:65278497:Esophagus;chr11:65236328:65278486:Gastric;chr11:65236367:65276489:Right Atrium;chr11:65236575:65276218:Brain Mid Frontal Lobe;chr11:65237462:65278685:Skeletal Muscle;chr11:65237615:65278442:Lung;chr11:65237705:65276621:Duodenum Smooth Muscle;chr11:65237718:65284903:Brain Inferior Temporal Lobe;chr11:65237743:65278340:Adrenal Gland;chr11:65237750:65276614:Stomach Smooth Muscle;chr11:65237768:65276595:Osteoblasts;chr11:65237773:65276387:NHDF-Ad;chr11:65237780:65272476:Ly4;chr11:65237780:65284684:Brain Cingulate Gyrus;chr11:65237782:65278645:HCT-116;chr11:65237792:65276547:K562;chr11:65237795:65276373:Aorta;chr11:65237797:65278629:Left Ventricle;chr11:65237834:65274588:Right Ventricle;chr11:65237836:65278618:HBL1;chr11:65237837:65278347:VACO 503;chr11:65237837:65278583:Small Intestine;chr11:65237876:65276599:Brain Hippocampus Middle 150;chr11:65237892:65277160:Ovary;chr11:65237900:65276454:CD4 Memory Primary

ASHG19SELNC2A100012312	2.4222	up	noncoding	ENST00000563601	noncoding	ENSG00000260589	RP11-390B4.5		2595	chr10:17683283-17685877:-	chr10:17674017:17740847:HBL1;chr10:17684185:17716976:Osteoblasts;chr10:17684235:17712354:CD4 Memory Primary 7pool;chr10:17684615:17727064:CD34 Primary RO01536;chr10:17684736:17689060:NHEK;chr10:17684838:17705250:K562;chr10:17684843:17690184:CD34 Primary RO01549;chr10:17687104:17727939:Jurkat
ASHG19SELNC2A100016157	3.4257	up	noncoding	ENST00000584252	noncoding	ENSG00000264269	RP11-15F12.1	Reliable	659	chr18:46550073-46574844:+	chr18:46539864:46589157:Fetal Thymus;chr18:46542392:46548534:VACO 503;chr18:46545572:46587035:DND41;chr18:46545833:46554362:Toledo;chr18:46567230:46591886:Toledo

ASHG19SELNC2A100010488	2.284	up	noncoding	ENST000004224 73	noncoding	ENSG00000233393	AP000688.29	2336	chr21:37477179-37481988:+	chr21:37469810:37530016:Esophagus;chr21:37485083:37539354:Gastric;chr21:37485091:37532822:Colon Crypt 2;chr21:37485332:37530139:Colon Crypt 3

ASHG19SELNC2A100004504	8.9395	up	noncoding	ENST00000506335	noncoding	ENSG00000249116	CTD-2194D22.3	Gold	425	chr5:1884080-1884763:+	chr5:1881509:1890183:HMEC
ASHG19SELNC2A100277814	2.0412	down	noncoding	ENST00000539543	noncoding	ENSG00000232527	RP11-14N7.2		605	chr1:148928345-148951595:+	chr1:148927828:148932626:Fetal Muscle;chr1:148928709:148932451:VACO 400
ASHG19SELNC2A100886364	2.2374	up	noncoding	ENST00000517582	noncoding	ENSG00000253447	RP11-619L12.4	Reliable	545	chr5:173508509-173513562:-	chr5:173491039:173548730:Brain Anterior Caudate

ASHG19SELNC2A100004005	3.8923	up	noncoding	ENST00000609813	noncoding	ENSG00000272719	CTB-161C1.1	Reliable	515	chr7:5596362-5596876:+	chr7:5558981:5573663:Lung;chr7:5564670:5573934:Sigmoid Colon;chr7:5564674:5572947:Left Ventricle;chr7:5564679:5573567:Stomach Smooth Muscle;chr7:5564693:5574167:Small Intestine;chr7:5564772:5572640:Right Atrium;chr7:5564850:5573400:Esophagus;chr7:5564889:5573668:Spleen;chr7:5564905:5574008:Aorta;chr7:5565201:5598917:Psoas Muscle;chr7:5565310:5573796:Gastric;chr7:5565322:5573222:LNCaP;chr7:5565338:5599172:Skeletal Muscle;chr7:5565359:5573202:Thymus;chr7:5565457:5572489:Right Ventricle;chr7:5565687:5744325:Fetal Muscle;chr7:5565836:5573211:VACO 503;chr7:5566034:5610583:Tonsil;chr7:5566111:5573678:Colon Crypt 2;chr7:5566754:5573047:Bladder;chr7:5567241:5610587:Ly3;chr7:5567405:5573812:Colon Crypt 3;chr7:5567495:5573966:DHL6;chr7:5568218:5573150:VACO 9m;chr7:5568917:5573171:H1;chr7:5586839:5615194:HeLa;chr7:5587306:5610600:VACO 503;chr7:5588087:5598869:Small Intestine;chr7:5588676:5639042:Lung;chr7:5588677:5615316:K562;chr7:5588711:5615292:Spleen;chr7:5588726:5603419:Sigmoid Colon;chr7:5588801:5603517:Gastric;chr7:5592299:5610301:VACO 400;chr7:5669472:5743117:Lung;chr7:5691544:5744744:Brain Hippocampus Middle

ASHG19SELNC2A100824122	2.2998	down	noncoding	ENST00000421616	noncoding	ENSG00000224066	RP4-622L5.7	786	chr1:32671099-32672415:-	chr1:32670496:32683809:Colon Crypt 1
ASHG19SELNC2A100013162	4.1488	down	noncoding	ENCT00000105829	noncoding	CATG00000012985	CATG00000012985.1	1505	chr12:97961003-97962507:-	chr12:97928133:97969609:MCF-7

ASHG19SELNC2A100004335	2.0211	up	noncoding	ENST00000416263	noncoding	ENSG00000229980	TOB1-AS1	1351	chr17:48944040-48945668:+	chr17:48911301:48946126:Brain Hippocampus Middle;chr17:48911900:48946435:Brain Cingulate Gyrus;chr17:48913503:48946005:Brain Anterior Caudate;chr17:48914089:48946508:Brain Hippocampus Middle 150;chr17:48914258:48946511:Brain Inferior Temporal Lobe;chr17:48914584:48945718:Brain Angular Gyrus;chr17:48921127:48947082:Skeletal Muscle;chr17:48921569:48946679:Fetal Muscle;chr17:48922220:48950371:Psoas Muscle;chr17:48922255:48945757:Lung;chr17:48922255:48946779:HeLa;chr17:48922302:48945677:Left Ventricle;chr17:48922332:48945577:Right Atrium;chr17:48922434:49028461:Fetal Intestine Large;chr17:48928158:48946406:Colon Crypt 1;chr17:48928263:48945553:Colon Crypt 2;chr17:48928752:48946765:Duodenum Smooth Muscle;chr17:48928781:48945692:Esophagus;chr17:48928840:48949433:Tonsil;chr17:48928846:48945623:Sigmoid Colon;chr17:48928866:48945814:Spleen;chr17:48928884:48962640:CD4 Memory Primary 7pool;chr17:48928908:48946693:HMEC;chr17:48928914:48946487:Colon Crypt 3;chr17:48928927:48946491:VACO 503;chr17:48928933:48945685:Gastric;chr17:48928936:48945737:Small Intestine;chr17:48929029:48946261:CD56;chr17:48929048:48946519:CD8 Memory 7pool;chr17:48929248:48946061:CD4 Naive Primary

ASHG19SELNC2A100001518	3.1591	up	noncoding	ENST0000056415 2	noncoding	ENSG00000260708	CTA-29F11.1	Reliable	670	chr22:47157791-47158460:-	chr22:47117108:47173881:Pancreatic islets;chr22:47120891:47171009:Left Ventricle;chr22:47120937:47151339:Right Atrium;chr22:47121652:47212523:Lung;chr22:47121797:47171731:Gastric;chr22:47122081:47199614:Ly 1;chr22:47122150:47151099:Colon Crypt 2;chr22:47123289:47173139:Small Intestine;chr22:47123707:47150600:CD4p CD25- Il17p PMAstim Th17;chr22:47124404:47151119:Right Ventricle;chr22:47126133:47189776:CD4p CD25- CD45ROp Memory;chr22:47126143:47189151:CD4p CD25- Il17- PMAstim Th;chr22:47126225:47156135:Fetal Intestine Large;chr22:47126257:47171810:Skeletal Muscle;chr22:47126276:47156003:Fetal Intestine;chr22:47126309:47189121:CD4 Memory Primary 7pool;chr22:47126406:47151212:Esophagus;chr22:47126491:47187734:Sigmoid Colon;chr22:47126710:47189645:CD56;chr22:47126759:47194919:CD14;chr22:47126769:47177146:Colon Crypt 1;chr22:47129710:47188820:Tonsil;chr22:47129860:47155690:HUVEC;chr22:47129977:47189264:CD4p CD25- CD45RAp Naive;chr22:47130708:47189319:Spleen;chr22:47154439:47199722:HBL1;chr22:47154582:47174370:DHL6;chr22:47156346:47184970:Ly3;chr22:47157222:47203732:CD20;chr22:47159882:47174367:CD19

ASHG19SELNC2A100276093	2.2422	up	noncoding	ENST00000426709	noncoding	ENSG00000231605	RP11-277B15.2	971	chr1:167145154-167165026:-	chr1:167034150:167060489:Small Intestine;chr1:167034909:167060442:VACO 503;chr1:167049094:167060421:Sigmoid Colon;chr1:167050266:167091822:Colon Crypt 1;chr1:167050811:167106924:RPMI-8402;chr1:167055109:167088187:Skeletal Muscle;chr1:167061598:167107859:Psoas Muscle;chr1:167073414:167102219:Jurkat;chr1:167099223:167128757:HSMMtube;chr1:167125796:167146570:VACO 503
ASHG19SELNC2A100015886	3.5426	up	noncoding	ENST00000446789	noncoding	ENSG00000235280	MCF2L-AS1	712	chr13:113621798-113623138:-	chr13:113528283:113706264:Pancreatic islets;chr13:113612236:113642719:Lung;chr13:113612250:113641315:Left Ventricle;chr13:113612285:113757507:Brain Hippocampus Middle;chr13:113633553:113668596:Gastric;chr13:113635638:113668660:Colon Crypt 3;chr13:113636776:113657006:Colon Crypt 1;chr13:113636800:113656732:Colon Crypt 2;chr13:113637770:113669757:LNCaP;chr13:113637848:113668615:VACO 400;chr13:113650879:113757822:Brain Inferior Temporal Lobe;chr13:113688788:113757608:Brain Cingulate Gyrus;chr13:113689195:113739320:Brain Anterior Caudate;chr13:113694428:113748561:Brain Hippocampus Middle 150;chr13:113695156:113757432:Brain Angular Gyrus;chr13:113698306:113718391:Brain Mid Frontal Lobe

ASHG19SELNC2A100003990	8.5713	up	noncoding	ENST00000602992	noncoding	ENSG00000270933	CTD-2227E11.1	Reliable	747	chr7:25988277-25989023:-	chr7:25979571:26012296:MM1S
ASHG19SELNC2A100006881	2.0658	up	noncoding	ENST00000553596	noncoding	ENSG00000258655	ARHGAP5-AS1		1375	chr14:32544625-32545999:-	chr14:32543177:32560581:HMEC;chr14:32543582:32560333:NHEK;chr14:32543731:32554126:Fetal Intestine Large;chr14:32543855:32554243:Fetal Intestine;chr14:32544066:32547960:Spleen;chr14:32544117:32548103:Esophagus

ASHG19SELNC2A100013976	2.0657	up	noncoding	MICT000002195 36	noncoding	CATG00000053508	CATG00000053508.1		3942	chr20:48367399-48378366:+	chr20:48358527:48449414:Esophagus;chr20:48363884:48468134:Spleen;chr20:48380887:48462335:CD14;chr20:48383102:48412240:HeLa;chr20:48384482:48449420:Sigmoid Colon;chr20:48384597:48423399:Adrenal Gland;chr20:48388660:48440895:Colon Crypt 1;chr20:48392206:48441118:Ly3;chr20:48401596:48463292:DHL6;chr20:48441986:48506238:Aorta;chr20:48490820:48534315:Pancreatic islets;chr20:48502151:48533649:Spleen;chr20:48502312:48555717:Brain Anterior Caudate;chr20:48502318:48555951:Brain Inferior Temporal Lobe;chr20:48524524:48534532:Colon Crypt 3;chr20:48525080:48555317:Fetal Intestine;chr20:48525121:48563588:Fetal Intestine Large;chr20:48525266:48541269:Colon Crypt 2;chr20:48525668:48555827:Colon Crypt 1;chr20:48525728:48555911:Small Intestine;chr20:48525739:48555882:Sigmoid Colon
ASHG19SELNC2A100017023	2.2087	down	noncoding	ENST00000607270	noncoding	ENSG00000272239	CTB-108O6.2	Reliable	237	chr5:146781323-146781559:+	chr5:146767343:146846803:Stomach Smooth Muscle;chr5:146775535:146870873:Ovary;chr5:146779092:146807795:Osteoblasts;chr5:146779966:146836223:Aorta;chr5:146810115:146868285:Fetal Muscle;chr5:146829832:146848915:H1;chr5:146845556:146867847:HUVEC;chr5:146858606:146866294:Osteoblasts;chr5:146861072:146865567:CD34 Primary RO01536;chr5:146861643:146865306:CD34 Primary RO01480
ASHG19SELNC2A100017065	3.56	up	noncoding	ENCT000001767 37	noncoding	CATG00000031817	CATG00000031817.1		2596	chr17:53522653-53525248:+	chr17:53467780:53534629:Adipose Nuclei;chr17:53493092:53532366:Duodenum Smooth Muscle

ASHG19SELNC2A100781733	2.3616	down	noncoding	ENST000004400 07	noncoding	ENSG00000229847	EMX2OS	754	chr10:119249996-119301845:-	chr10:119291814:119313346:NHDF-Ad

ASHG19SUPERLNC2A100001252	2.2981	up	noncoding	ENST0000055405 5	noncoding	ENSG00000258757	RP11-841O20.2	Reliable	581	chr14:53107557-53108284:+	
ASHG19SELNC2A100015910	3.1591	up	noncoding	ENST0000041519 5	noncoding	ENSG00000235781	XXbac-BPG249D20.9	Gold	851	chr6:30484043-30486994:+	chr6:30456364:30512797:Tonsil;chr6:30456489:30511165:Spleen;chr6:30456531:30496380:Sigmoid Colon;chr6:30459866:30525894:Small Intestine;chr6:30483089:30526577:CD8 Memory 7pool;chr6:30483105:30525972:Pancreatic islets;chr6:30522089:30526621:CD4 Memory Primary 7pool;chr6:30522426:30525860:Fetal Muscle;chr6:30522440:30525857:NHLF;chr6:30522461:30526297:CD8 primiary;chr6:30522483:30526459:CD4p CD25-Il17p PMAstim Th17
ASHG19SELNC2A100043741	2.0072	up	noncoding	ENST0000041412 0	noncoding	ENSG00000214145	LINC00887		2880	chr3:194018988-194030589:-	chr3:193963033:194033612:RPMI-8402;chr3:193966874:194004090:Fetal Muscle;chr3:193971946:193990154:HUVEC;chr3:193982988:193997694:Adrenal Gland;chr3:193983051:193997202:Ovary;chr3:193988211:194015486:Pancreas;chr3:194004041:194015552:H1;chr3:194022156:194028209:MM1S;chr3:194032286:194055090:HeLa

ASHG19SELNC2A100011218	2.0876	up	noncoding	ENST00000428142	noncoding	ENSG00000230709	AC104024.1	Reliable	2201	chr17:16879313-16884667:-	chr17:16841711:16905645:Toledo;chr17:16846365:16895884:CD20;chr17:16848995:16895735:HBL1;chr17:16849963:16895450:GM12878;chr17:16857543:16895465:CD19 Primary;chr17:16867134:16895512:Ly3;chr17:16868372:16895656:Tonsil;chr17:16868489:16895794:MM1S;chr17:16871372:16895547:DND41;chr17:16904912:16994597:VACO 503;chr17:16904926:17032793:Pancreatic islets;chr17:16904952:16957284:Fetal Intestine Large;chr17:16905178:17024731:Fetal Intestine;chr17:16910885:17057075:Brain Hippocampus Middle;chr17:16910923:16948823:HCT-116;chr17:16911039:17056941:Lung;chr17:16911053:16951863:HeLa;chr17:16911068:16932103:HCC1954;chr17:16911294:17030580:Gastric;chr17:16912112:16955078:u87;chr17:16912295:17008666:Pancreas;chr17:16912540:16979530:HSMMtube;chr17:16912929:16985611:Osteoblasts;chr17:16912952:16978959:HMEC;chr17:16912965:17059812:Brain Cingulate Gyrus;chr17:16913012:17063100:Brain Anterior Caudate;chr17:16913151:16961046:Left Ventricle;chr17:16913179:16932552:VACO 9m;chr17:16913199:16979561:HSMM;chr17:16913231:16978948:Skeletal Muscle Myoblast;chr17:16913232:16967371:Esophagus;chr17:16913280:16957311:Colon Crypt 1;chr17:16913349:16936008:Colon Crypt 2;chr17:16921052:16995066:LNCaP;chr17:16930832

ASHG19SELNC2A100005541	2.2482	up	noncoding	FTMT22200011278	noncoding	CATG00000090647	CATG00000090647.1	1625	chr6:158187915-158189539:-	chr6:158168374:158196042:CD4p CD25- Il17-PMAstim Th;chr6:158174308:158195733:CD4p CD25- Il17p PMAstim Th17

ASHG19SELNC2A100614107	10.351	down	noncoding	ENST0000558888	noncoding	ENSG00000259370	RP11-1069G10.1	2978	chr15:63124323-63127596:-	chr15:63125186:63201059:Stomach Smooth Muscle;chr15:63165410:63194089:Duodenum Smooth Muscle;chr15:63171167:63190796:HUVEC;chr15:63178350:63198071:u87;chr15:63178431:63190422:Astrocytes;chr15:63187301:63234200:Left Ventricle

ASHG19SELNC2A101129761	2.222	down	noncoding	ENST00000565256	noncoding	ENSG00000234912	LINC00338	1234	chr17:75084831-75091065:+	chr17:75078960:75107007:MM1S;chr17:75089372:75177504:Gastric;chr17:75089434:75179269:Lung;chr17:75090732:75185340:Brain Inferior Temporal Lobe;chr17:75090920:75183340:Brain Cingulate Gyrus;chr17:75091070:75162154:Brain Angular Gyrus;chr17:75091936:75244237:Brain Hippocampus Middle;chr17:75092038:75162995:Skeletal Muscle;chr17:75092246:75210377:Brain Anterior Caudate;chr17:75092282:75162618:Adipose Nuclei;chr17:75092469:75179721:Brain Hippocampus Middle 150;chr17:75092478:75157123:Osteoblasts;chr17:75092668:75179784:Fetal Muscle;chr17:75092686:75161313:Left Ventricle;chr17:75092688:75178581:Psoas Muscle;chr17:75092740:75185725:Spleen;chr17:75092774:75145755:Small Intestine;chr17:75092796:75125744:Brain Mid Frontal Lobe;chr17:75092800:75205783:Adrenal Gland;chr17:75092825:75161754:Right Atrium;chr17:75094383:75128210:HSMMtube;chr17:75110737:75126418:HUVEC;chr17:75111947:75130104:Panc1;chr17:75117759:75142519:HMEC;chr17:75130900:75161298:MM1S;chr17:75134520:75145850:HUVEC;chr17:75134802:75185828:Ly4

ASHG19SELNC2A100011950	6.6766	up	noncoding	ENST00000457079	noncoding	ENSG00000224652	LINC00885	1828	chr3:195869507-195887761:+	chr3:195834121:195887811:CD4 Memory Primary 7pool;chr3:195834564:195873620:K562;chr3:195853704:195896426:HCC1954;chr3:195886180:195930991:CD14;chr3:195886382:195921896:Small Intestine;chr3:195886668:195921587:Colon Crypt 2;chr3:195905320:195923896:Ly3;chr3:195905452:195920877:Fetal Thymus;chr3:195905480:195930175:HBL1;chr3:195906708:195922858:CD34 adult;chr3:195908864:195922707:CD34 fetal;chr3:195913496:195955891:Ovary

ASHG19SELNC2A100088326	2.8138	down	noncoding	ENST000004454 61	noncoding	ENSG00000215386	LINC00478	710	chr21:17566715-17979542:+	chr21:17675058:17727232:LNCaP

ASHG19SELNC2A100088326	2.8138	down	noncoding	ENST00000445461	noncoding	ENSG00000215386	LINC00478	710	chr21:17566715-17979542:+	chr21:17776670:17798151:Fetal Muscle;chr21:17790506:17800624:HUVEC

ASHG19SELNC2A100088326	2.8138	down	noncoding	ENST000004454 61	noncoding	ENSG00000215386	LINC00478	710	chr21:17566715-17979542:+	chr21:17904299:17910609:Fetal Muscle

ASHG19SELNC2A100088326	2.8138	down	noncoding	ENST00000445461	noncoding	ENSG00000215386	LINC00478	710	chr21:17566715-17979542:+	chr21:17941411:17967670:u87

ASHG19SELNC2A100009738	5.5753	down	noncoding	ENST0000433442	noncoding	ENSG00000236591	RP11-162J8.3	Reliable	130	chr6:149348836-149353709:-	chr6:149319908:149356643:u87;chr6:149349238:149419120:DND41;chr6:149349433:149410166:HBL1;chr6:149349666:149401648:Toledo;chr6:149349702:149401761:Ly4;chr6:149349844:149403817:Ly3;chr6:149352296:149386196:HSMMtube
ASHG19SELNC2A100016268	4.2494	up	noncoding	ENCT0000263909	noncoding	CATG00000054027	CATG00000054027.1		1226	chr20:344934-351058:-	chr20:326788:366598:K562;chr20:327187:354654:VACO 400;chr20:327465:364442:CD8 Memory 7pool;chr20:328015:364988:VACO 503;chr20:340590:365017:NHEK;chr20:342413:364265:HeLa;chr20:342738:359360:HCC1954;chr20:343643:365042:CD4 Memory Primary 7pool

ASHG19SELNC2A100001934	3.0292	up	noncoding	ENST000004494977	noncoding	ENSG00000226012	AP001434.2		287	chr21:39609139-39610123:-	chr21:39589919:39725911:u87;chr21:39590687:39684328:CD14;chr21:39610820:39701924:HSMM;chr21:39611470:39699447:Skeletal Muscle Myoblast;chr21:39611805:39725186:Osteoblasts;chr21:39634533:39700615:HSMMtube;chr21:39634821:39699618:NHDF-Ad;chr21:39638616:39664746:NHEK
ASHG19SELNC2A100006908	2.3455	up	noncoding	ENST00000568809	noncoding	ENSG00000261295	RP11-524D16__A.3	Reliable	652	chrX:99928327-99928978:+	chrX:99897948:99941436:Adipose Nuclei;chrX:99897993:99941188:HUVEC

ASHG19SELNC2A100000712	29.651	up	noncoding	ENST000005627 60	noncoding	ENSG00000261437	RP11-22C11.2		2183	chr8:95649513-95651695:-	chr8:95625168:95655730:Gastric;chr8:95625246:95666334:Colon Crypt 1;chr8:95645639:95655599:Colon Crypt 2;chr8:95649813:95665769:Colon Crypt 3;chr8:95650653:95671119:HCC1954;chr8:95650807:95671072:NHEK;chr8:95651039:95655110:VACO9m
ASHG19SELNC2A100002183	2.2457	up	noncoding	ENST00000512712	noncoding	ENSG00000249673	NOP14-AS1	Gold	3188	chr4:2937273-2943778:+	chr4:2925304:2963931:LNCaP;chr4:2925418:2963890:Gastric;chr4:2931220:2946939:Colon Crypt 3;chr4:2931273:2945707:Colon Crypt 2;chr4:2936426:2940017:CD4p CD25- Il17p PMAstim Th17;chr4:2936474:2940031:NHEK

ASHG19SELNC2A100981099	2.3468	down	noncoding	ENST00000558071	noncoding	ENSG00000259347	RP11-798K3.2	Reliable	741	chr15:67278699-67351591:-	chr15:67273066:67444892:HSMMtube;chr15:67303551:67494412:Osteoblasts;chr15:67313358:67492820:Panc1;chr15:67315459:67370465:HeLa;chr15:67315776:67489681:Esophagus;chr15:67315940:67343228:HMEC;chr15:67315957:67490205:Small Intestine;chr15:67316001:67381111:Fetal Intestine;chr15:67316023:67455233:Fetal Intestine Large;chr15:67317794:67468217:Colon Crypt 1;chr15:67331697:67482584:Gastric;chr15:67333711:67343350:Skeletal Muscle Myoblast;chr15:67333711:67343392:HSMM;chr15:67335503:67451448:Adipose Nuclei;chr15:67336274:67492619:Lung;chr15:67336292:67374242:Aorta;chr15:67336346:67360297:Pancreas;chr15:67336445:67451264:Right Atrium;chr15:67339819:67443576:Duodenum Smooth Muscle;chr15:67340158:67490067:Sigmoid Colon;chr15:67340644:67441951:Colon Crypt 2;chr15:67342858:67447290:Ly1;chr15:67347840:67491869:CD20;chr15:67348117:67430609:CD8 primiary;chr15:67354926:67408793:Ly4;chr15:67355589:67420519:HCT-116;chr15:67355772:67469893:CD4p CD25- Il17-PMAstim Th;chr15:67355940:67496057:Psoas Muscle;chr15:67355971:67443321:CD4 Memory Primary 7pool;chr15:67355995:67443338:CD4p CD25- Il17p PMAstim Th17;chr15:67355996:67443611:CD4p CD25-CD45ROp Memory;chr15:67356047:67391700:K562;chr15:673

ASHG19SELNC2A100007702	2.0012	up	noncoding	ENST0000058667 5	noncoding	ENSG00000267304	AC004637.1	Reliable	482	chr19:3672580-3674293:+	chr19:3666824:3700550:Stomach Smooth Muscle;chr19:3666968:3674428:Aorta;chr19:3667225:3723445:Esophagus;chr19:3687476:3726402:Gastric;chr19:3707627:3726433:Sigmoid Colon;chr19:3707697:3726343:Small Intestine;chr19:3707812:3726260:Colon Crypt 1;chr19:3707830:3726357:Colon Crypt 2
ASHG19SELNC2A100009234	2.4286	up	noncoding	ENST0000060523 3	noncoding	ENSG00000270344	RP11-734K2.4	Reliable	338	chr12:89919431-89934079:+	chr12:89885863:89920609:Fetal Intestine;chr12:89886006:89933579:Fetal Intestine Large
ASHG19SELNC2A100000460	7.028	up	noncoding	ENST0000043367 3	noncoding	ENSG00000236682	AC068282.3	Gold	2690	chr2:128146706-128158156:+	chr2:128107200:128174489:CD14;chr2:128143043:128150313:CD20;chr2:128143818:128166773:Fetal Intestine Large;chr2:128144076:128181450:Fetal Intestine;chr2:128144112:128148979:CD19 Primary;chr2:128144245:128148044:Spleen;chr2:128144272:128166882:Sigmoid Colon;chr2:128144280:128181466:Colon Crypt 1;chr2:128144282:128174209:Small Intestine;chr2:128144293:128181545:Colon Crypt 3;chr2:128144334:128169985:Lung;chr2:128144434:128181412:Colon Crypt 2
ASHG19SELNC2A100009527	2.8538	down	noncoding	ENST0000032673 4	noncoding	ENSG00000177757	FAM87B		1944	chr1:752751-755214:+	chr1:751471:763425:Osteoblasts;chr1:752366:774266:Left Ventricle;chr1:752370:772533:Lung;chr1:752489:794895:Pancreatic islets;chr1:752546:772570:Right Atrium;chr1:760218:786035:DND41;chr1:771395:794487:Jurkat

ASHG19SELNC2A100010082	3.1499	up	noncoding	ENST0000606539	noncoding	ENSG00000271969	U47924.29	Reliable	434	chr12:7074112-7074545:+	chr12:7021310:7026081:H2171;chr12:7022600:7051380:Brain Cingulate Gyrus;chr12:7022685:7051255:Stomach Smooth Muscle;chr12:7022964:7047898:GLC16;chr12:7032434:7039694:H2171;chr12:7032586:7039696:Lung;chr12:7033029:7039703:Esophagus;chr12:7033041:7048410:Right Atrium;chr12:7033067:7050686:Brain Angular Gyrus;chr12:7033253:7051404:LNCaP;chr12:7033291:7048082:Fetal Muscle;chr12:7033383:7048045:Ovary;chr12:7033514:7048028:H1;chr12:7033555:7047595:Bladder;chr12:7033612:7047812:Pancreas;chr12:7033631:7055666:VACO 503;chr12:7033645:7047280:Right Ventricle;chr12:7044264:7072113:CD14;chr12:7044426:7072735:CD34 Primary RO01536;chr12:7044970:7072648:CD19 Primary;chr12:7045010:7071657:CD4 Naive Primary 7pool;chr12:7045041:7071857:CD4p CD225int CD127p Tmem;chr12:7045046:7072000:CD3;chr12:7045125:7071857:CD8 Naive 7pool;chr12:7045276:7072350:CD56;chr12:7045399:7071773:Ly3;chr12:7045533:7071721:CD8 Naive 8pool;chr12:7045550:7072074:CD4 Naive Primary 8pool;chr12:7045555:7071876:CD4 Memory Primary 8pool;chr12:7045612:7071479:Toledo;chr12:7045636:7072391:Thymus;chr12:7045722:7069352:Ly4;chr12:7045931:7071909:GM12878;chr12:7050709:7057

ASHG19SELNC2A100929764	3.3227	up	noncoding	ENST000005138 68	noncoding	ENSG00000249859	PVT1	1699	chr8:128902874-129113499:+	chr8:128793812:128824254:HCC1954;chr8:128793447:128824370:MCF-7;chr8:128800421:128841375:NCI-H82;chr8:128800542:128824452:DHL6;chr8:128805716:128945203:Panc1;chr8:128805831:128844962:NHDF-Ad;chr8:128805875:128965109:Osteoblasts;chr8:128805887:128813518:GLC16;chr8:128805907:128824571:NHLF;chr8:128805989:128825280:HUVEC;chr8:128810486:128990391:LNCaP;chr8:128810566:128907192:Ovary;chr8:128814718:128836148:H1;chr8:128843114:128888912:HUVEC;chr8:128843308:128883898:Astrocytes;chr8:128843389:128868801:HSMM tube;chr8:128843496:128868262:HSMM;chr8:128843510:128868207:Skeletal Muscle Myoblast;chr8:128857207:128868734:IMR90;chr8:128857956:128964803:Adrenal Gland;chr8:128860824:128884150:MCF-7;chr8:128860946:128903357:HCC1954;chr8:128861014:128868643:NHLF;chr8:128862297:128868773:NHDF-Ad;chr8:128862757:128888657:HMEC;chr8:128863156:128888485:NHEK;chr8:128863390:128904642:Esophagus;chr8:128882161:128964080:HSMMtube;chr8:128900610:128941873:NHLF;chr8:128901448:128947020:HUVEC;chr8:128901547:128941881:NHDF-Ad;chr8:128901634:128963789:u87;chr8:128901635:128963280:HSMM;chr8:128901711:128963214:Astrocytes;chr8:128901721:128963158:Skeletal Muscle

ASHG19SELNC2A100929764	3.3227	up	noncoding	ENST000005138 68	noncoding	ENSG00000249859	PVT1	1699	chr8:128902874-129113499:+	chr8:129045436:129065022:CD14;chr8:129046437:129091141:K562;chr8:129057564:129063683:CD34 adult;chr8:129058554:129143259:HBL1;chr8:129059294:129143247:Ly3;chr8:129087461:129118095:CD4p CD25- Il17- PMAstim Th

ASHG19SELNC2A100668904	3.3284	down	noncoding	ENST00000412788	noncoding	ENSG00000130600	H19		2281	chr11:2016406-2019044:-	chr11:1924529:1991431:Left Ventricle;chr11:1927730:1991499:Right Atrium;chr11:1937073:1954345:Skeletal Muscle;chr11:1937589:1954304:Fetal Muscle;chr11:1967535:1971731:Colon Crypt 3;chr11:1967588:1971636:VACO 400;chr11:1967605:1976290:Adrenal Gland;chr11:1968470:1971027:Colon Crypt 2;chr11:1968499:1981090:Fetal Muscle;chr11:1968517:2015633:HSMM;chr11:1968536:2009985:Skeletal Muscle Myoblast;chr11:1970465:2015366:Ovary;chr11:1971932:1976153:VACO 400;chr11:1982329:1993706:Fetal Muscle;chr11:1984055:1999144:Psoas Muscle;chr11:1986257:1993492:HSMMtube;chr11:1987729:1994124:VACO 400;chr11:1999934:2007267:VACO 400;chr11:2007360:2011387:VACO 400;chr11:2007505:2020554:Fetal Intestine Large;chr11:2007634:2020503:Fetal Intestine;chr11:2008546:2021217:Fetal Muscle;chr11:2011395:2019037:VACO 400

ASHG19SELNC2A100586240	3.4078	down	noncoding	ENST000004270 20	noncoding	ENSG00000205054	LINC01121	722	chr2:45392009-45419404:-	chr2:45389724:45402031:Stomach Smooth Muscle;chr2:45393131:45428128:HSMM;chr2:45394542:45428363:HSMMtube;chr2:45394767:45427999:Skeletal Muscle Myoblast;chr2:45395340:45412703:Osteoblasts

ASHG19SELNC2A100342200	2.2239	down	noncoding	MICT000000626 23	noncoding	CATG00000006004	CATG00000006004.1	1632	chr11:69040085-69042067:-	chr11:69019019:69070837:HCC1954;chr11:69035833:69089097:Pancreatic islets;chr11:69040170:69070245:Esophagus;chr11:69040207:69079860:Gastric;chr11:69040227:69070579:Colon Crypt 1;chr11:69040530:69066696:Pancreas;chr11:69040623:69070143:Colon Crypt 2;chr11:69041149:69070238:VACO 400;chr11:69041330:69070368:Sigmoid Colon;chr11:69041617:69072027:VACO 503;chr11:69043247:69081064:Ly4;chr11:69048050:69089929:u87;chr11:69048698:69070633:VACO 9m;chr11:69059646:69088726:Panc1;chr11:69059793:69071009:HCT-116;chr11:69059907:69089985:HUVEC;chr11:69061501:69070316:K562;chr11:69063032:69089949:Osteoblasts;chr11:69063158:69072297:HeLa;chr11:69063267:69089937:NHLF;chr11:69063296:69089921:NHDF-Ad;chr11:69063593:69089932:HSMM;chr11:69063607:69089917:Skeletal Muscle Myoblast;chr11:69063939:69070881:Astrocytes

ASHG19SELNC2A100006093	2.6247	up	noncoding	ENST000005452 02	noncoding	ENSG00000255980	AP000439.1	Reliable	2376	chr11:69240458-69244389:+	chr11:69234493:69260008:VACO 400;chr11:69256707:69279345:Fetal Muscle;chr11:69276903:69296403:Colon Crypt 2
ASHG19SELNC2A100012372	2.2558	up	noncoding	ENST0000060705 2	noncoding	ENSG00000271870	RP11-97C16.1	Reliable	494	chr3:3194626-3195119:+	chr3:3167247:3235052:CD4p CD25- CD45RAp Naive;chr3:3167559:3235215:CD4p CD25- Il17- PMAstim Th;chr3:3190673:3235082:CD4 Memory Primary 7pool;chr3:3191066:3235177:CD8 primiary

ASHG19SELNC2A100004698	2.1775	up	noncoding	ENST00000609755	noncoding	ENSG00000273230	RP11-1246C19.1	Reliable	3026	chr7:1504133-1507158:-	chr7:1483916:1507349:Adrenal Gland;chr7:1486001:1507516:Spleen;chr7:1486213:1515763:Colon Crypt 3;chr7:1486269:1507327:Sigmoid Colon;chr7:1486352:1514892:Esophagus;chr7:1487024:1514387:Gastric;chr7:1489958:1514795:Colon Crypt 1;chr7:1494100:1514700:Colon Crypt 2;chr7:1494178:1514569:VACO 400;chr7:1494491:1507434:Fetal Intestine Large;chr7:1496273:1503283:K562;chr7:1496418:1502047:CD34 adult
ASHG19SELNC2A100017434	2.7354	down	noncoding	ENST00000434990	noncoding	ENSG00000236469	AC007040.8	Gold	570	chr2:71229661-71291873:-	chr2:71289500:71312132:CD14;chr2:71289657:71311762:CD20;chr2:71289943:71300709:Ly3;chr2:71290095:71300515:DHL6;chr2:71290265:71301717:Esophagus;chr2:71290385:71301563:CD19 Primary;chr2:71290494:71311637:Spleen
ASHG19SELNC2A100534975	2.7915	down	noncoding	NR_138484	noncoding	ENSG00000134255	CEPT1		2408	chr1:111682785-111727726:+	chr1:111658932:111700441:CD20;chr1:111667233:111691882:Tonsil;chr1:111668533:111685066:Ly3

ASHG19SELNC2A100534975	2.7915	down	noncoding	NR_138484	noncoding	ENSG00000134255	CEPT1	2408	chr1:111682785-111727726:+	chr1:111707642:111732738:CD20;chr1:111720463:111773369:CD4p CD25- CD45RAp Naive;chr1:111720877:111748580:Adipose Nuclei;chr1:111721356:111773351:CD4p CD25- Il17- PMAstim Th;chr1:111721546:111771793:CD8 primiary;chr1:111722467:111770686:CD56;chr1:111722584:111771572:CD3;chr1:111724894:111769353:CD4 Memory Primary 7pool;chr1:111725046:111766110:CD14;chr1:111725074:111771067:CD4p CD25- CD45ROp Memory;chr1:111727706:111778392:DND41;chr1:111730652:111771012:CD8 Naive 8pool;chr1:111730953:111769306:CD4p CD25- Il17p PMAstim Th17;chr1:111731924:111786958:Spleen;chr1:111732684:111775273:Tonsil;chr1:111732689:111766358:Sigmoid Colon;chr1:111732755:111766318:Small Intestine;chr1:111734292:111748221:Gastric;chr1:111734374:111769514:GM12878;chr1:111734461:111748269:Esophagus;chr1:111734723:111748126:Colon Crypt 2;chr1:111734737:111768995:CD19 Primary;chr1:111734752:111768907:CD4p CD225int CD127p Tmem;chr1:111734884:111769467:CD8 Memory 7pool;chr1:111734917:111772142:Fetal Thymus;chr1:111734952:111769864:CD4 Naive Primary 8pool;chr1:111735057:111770041:CD8 Naive 7pool;chr1:111735190:111768994:CD4 Memory Primary 8pool;chr1:111735195:111775083:Ly1;chr1:1117352

ASHG19SELNC2A100013127	2.1068	up	noncoding	ENST00000602919	noncoding	ENSG00000270177	CTD-2410N18.3	Reliable	1418	chr5:133562101-133563518:+	chr5:133554238:133583971:Skeletal Muscle;chr5:133558980:133563313:CD4p CD25-Il17p PMAstim Th17;chr5:133559389:133592083:Left Ventricle
ASHG19SELNC2A100052488	2.3065	up	noncoding	ENST00000430679	noncoding	ENSG00000234139	RP4-550H1.4		458	chr20:34660363-34662831:+	chr20:34651707:34655126:VACO 503;chr20:34651941:34686940:Esophagus;chr20:34652078:34694626:Colon Crypt 3;chr20:34667259:34691804:HeLa;chr20:34668921:34721786:VACO 503;chr20:34670504:34711458:Gastric;chr20:34672865:34711004:Sigmoid Colon;chr20:34672908:34711170:Colon Crypt 1;chr20:34673151:34720941:Colon Crypt 2;chr20:34679127:34746032:Adipose Nuclei;chr20:34679509:34694907:Pancreas;chr20:34680074:34731861:Ly4;chr20:34730341:34785517:Adrenal Gland
ASHG19SELNC2A100012947	4.427	up	noncoding	ENST00000502995	noncoding	ENSG00000248898	CTD-2288O8.1	Reliable	593	chr5:51971027-52083860:-	chr5:52000414:52040505:DND41;chr5:52005413:52040298:Jurkat
ASHG19SELNC2A100012947	4.427	up	noncoding	ENST00000502995	noncoding	ENSG00000248898	CTD-2288O8.1	Reliable	593	chr5:51971027-52083860:-	chr5:52052598:52138481:Adipose Nuclei;chr5:52069539:52160201:Spleen;chr5:52082216:52172660:Duodenum Smooth Muscle;chr5:52093242:52106729:IMR90;chr5:52094567:52106662:NHLF;chr5:52132254:52162287:IMR90

ASHG19SELNC2A100893215	4.0351	down	noncoding	ENCT000001520 82	noncoding	CATG00000025802	CATG00000025802.1		2080	chr15:87711187-87733604:-	chr15:87729919:87764695:MCF-7
ASHG19SELNC2A100935709	2.5612	up	noncoding	ENST00000562577	noncoding	ENSG00000260493	RP11-219B4.7	Reliable	487	chr8:86084312-86089276:-	chr8:86083942:86113880:DHL6;chr8:86083947:86114627:Ly1;chr8:86086319:86134266:CD20;chr8:86087035:86114989:CD19 Primary;chr8:86094080:86111892:Ly4
ASHG19SELNC2A100007733	2.4314	up	noncoding	ENST00000610202	noncoding	ENSG00000272902	RP11-299H21.1	Reliable	2476	chr2:101768122-101771872:+	chr2:101662520:101769918:Panc1;chr2:101720955:101770878:Left Ventricle;chr2:101722090:101769940:Skeletal Muscle;chr2:101722600:101769642:Lung;chr2:101722728:101769515:Gastric;chr2:101722759:101747184:Psoas Muscle;chr2:101722802:101770839:Right Atrium;chr2:101725422:101741815:Right Ventricle;chr2:101725706:101744776:HUVEC

ASHG19SELNC2A100013855	2.8802	up	noncoding	ENST0000056389 7	noncoding	ENSG00000261005	CTB-58E17.1	Reliable	2296	chr17:36858705-36861000:+	chr17:36841483:36864135:Spleen;chr17:36849347:36863638:Left Ventricle;chr17:36849416:36867442:Fetal Muscle;chr17:36849427:36868217:Lung;chr17:36849443:36863581:Small Intestine;chr17:36849446:36869721:Right Atrium;chr17:36849486:36864348:Sigmoid Colon;chr17:36849658:36862895:Right Ventricle;chr17:36849707:36864129:Gastric;chr17:36852020:36863435:MM1S;chr17:36852687:36863936:Tonsil;chr17:36854835:36891315:CD4p CD25- Il17p PMAstim Th17;chr17:36854933:36891548:CD4p CD25- Il17- PMAstim Th;chr17:36856374:36863732:Ly3;chr17:36856433:36863910:CD56;chr17:36856888:36888783:CD8 primiary;chr17:36857029:36864323:Pancreatic islets;chr17:36857157:36863577:Esophagus;chr17:36857165:36863536:Adrenal Gland;chr17:36857173:36861522:K562;chr17:36857186:36861774:HeLa;chr17:36857283:36862851:Pancreas;chr17:36857283:36862900:VACO 9m;chr17:36857336:36862962:Thymus;chr17:36857431:36876540:VACO 503;chr17:36880595:36907474:Gastric;chr17:36880935:36900163:Colon Crypt 2;chr17:36881475:36907782:Small Intestine;chr17:36881476:36907973:Colon Crypt 1;chr17:36883977:36907951:Lung;chr17:36886485:36906657:Pancreas;chr17:36886615:36890751:LNCaP

ASHG19SELNC2A100017831	7.6401	down	noncoding	ENCT0000036785	noncoding	CATG00000090897	CATG00000090897.1	1470	chr1:205250559-205265599:-	chr1:205183427:205322469:Toledo;chr1:205195003:205257756:Brain Inferior Temporal Lobe;chr1:205195303:205291843:Brain Hippocampus Middle;chr1:205195357:205255709:Brain Cingulate Gyrus;chr1:205195435:205256085:Brain Hippocampus Middle 150;chr1:205198707:205257315:Brain Anterior Caudate;chr1:205198731:205254801:Brain Angular Gyrus;chr1:205212503:205240648:Brain Mid Frontal Lobe;chr1:205216538:205294796:Pancreatic islets;chr1:205226962:205289498:Osteoblasts;chr1:205242037:205294994:HBL1;chr1:205242169:205295027:Ly1;chr1:205242236:205295022:Ly3;chr1:205242267:205294889:DHL6;chr1:205242326:205284722:Ly4;chr1:205242383:205291943:CD20;chr1:205242393:205294944:Tonsil;chr1:205242416:205289483:Sigmoid Colon;chr1:205242795:205294653:VACO 400;chr1:205244073:205289461:Small Intestine;chr1:205244127:205289484:Esophagus;chr1:205244180:205263684:Thymus;chr1:205252499:205258684:MM1S;chr1:205252644:205288278:H1;chr1:205252675:205289470:Spleen;chr1:205252877:205284629:IMR90;chr1:205252917:205296755:u87;chr1:205253201:205283802:Astrocytes;chr1:205253543:205294850:Fetal Intestine Large;chr1:205267271:205294703:Pancreas;chr1:205270131:205289265:LNCaP;chr1:205305762:205355411:Ovary
ASHG19SELNC2A100006843	3.6245	up	noncoding	ENST00000525023	noncoding	ENSG00000254690	GS1-393G12.12	444	chr8:145538253-145538801:-	chr8:145517464:145548516:Adrenal Gland;chr8:145519520:145562137:HCT-116;chr8:145536383:145555666:Colon Crypt 3;chr8:145536386:145551382:Small Intestine;chr8:145537471:145550558:Colon Crypt 1;chr8:145537473:145555571:Colon Crypt 2

ASHG19SELNC2A100004083	3.2857	down	noncoding	FTMT21200006826	noncoding	CATG00000062028	CATG00000062028.1		1747	chr3:141175340-141177086:+	chr3:141012280:141141298:u87;chr3:141029873:141089855:Fetal Intestine Large;chr3:141029943:141089529:Fetal Intestine;chr3:141049278:141207535:Osteoblasts;chr3:141071097:141089363:K562;chr3:141079002:141162760:Brain Hippocampus Middle;chr3:141079164:141144945:HBL1;chr3:141079175:141160355:MM1S;chr3:141079314:141089545:HSMMtube;chr3:141079331:141151807:Brain Anterior Caudate;chr3:141079389:141162728:Brain Inferior Temporal Lobe;chr3:141079392:141161781:Brain Hippocampus Middle 150;chr3:141079393:141151934:Brain Angular Gyrus;chr3:141079404:141170275:Aorta;chr3:141079417:141168709:IMR90;chr3:141079427:141159821:Brain Cingulate Gyrus;chr3:141079440:141175901:Small Intestine;chr3:141079477:141089627:HUVEC;chr3:141079503:141132303:GM12878;chr3:141079528:141103947:Astrocytes;chr3:141079553:141134828:Tonsil;chr3:141079598:141089459:NHLF;chr3:141079660:141089224:HSMM;chr3:141079674:141089224:Skeletal Muscle Myoblast;chr3:141083519:141136674:NHDF-Ad;chr3:141083866:141089271:HMEC;chr3:141084544:141132639:NHEK;chr3:141084779:141146409:CD8 Memory 7pool;chr3:141084784:141088680:VACO 9m;chr3:141084789:141146536:CD4 Memory

ASHG19SELNC2A100007729	2.1894	up	noncoding	ENST0000060831 8	noncoding	ENSG00000272993	RP11-196G18.24	Reliable	671	chr1:149817383-149818053:+	chr1:149800183:149806539:NCI-H82;chr1:149800302:149806721:Stomach Smooth Muscle;chr1:149800343:149806744:Fetal Thymus;chr1:149800366:149806664:Duodenum Smooth Muscle;chr1:149800408:149836226:Panc1;chr1:149801218:149806733:Fetal Muscle;chr1:149801461:149806380:Fetal Intestine Large;chr1:149801554:149806661:Fetal Intestine;chr1:149801661:149805683:Small Intestine;chr1:149801665:149807694:CD4 Memory Primary 7pool;chr1:149801740:149806764:HeLa;chr1:149801747:149806411:NHEK;chr1:149801769:149806231:Spleen;chr1:149801785:149806372:Sigmoid Colon;chr1:149801798:149806278:Esophagus;chr1:149801803:149805524:Right Atrium;chr1:149801810:149805853:Left Ventricle;chr1:149801815:149805529:Lung;chr1:149801950:149806735:HCC1954;chr1:149801975:149805417:Gastric;chr1:149802018:149806664:CD8 Memory 7pool;chr1:149802074:149806387:K562;chr1:149802097:149805677:Thymus;chr1:149802251:149806522:CD4 Memory Primary 8pool;chr1:149802448:149806583:CD8 Naive 7pool;chr1:149802939:149806808:CD4 Naive Primary 8pool;chr1:149807697:149814294:NCI-H82;chr1:149808078:149815957:K562;chr1:149808745:149816200:HeLa;chr1:149808786:149818154:Fet

ASHG19SELNC2A100013551	2.0194	up	noncoding	ENST00000436105	noncoding	ENSG00000231638	AC011738.4	2916	chr7:43548327-43562141:-	chr7:43546657:43562481:Jurkat

ASHG19SELNC2A100002493	5.6398	down	noncoding	FTMT21200007268	noncoding	CATG00000062183	CATG00000062183.1	1165	chr3:149160771-149161935:+	chr3:149159355:149198593:Osteoblasts;chr3:149160169:149226975:HSMMtube;chr3:149160625:149193347:HSMM;chr3:149192850:149381018:Panc1;chr3:149244777:149377700:HUVEC;chr3:149245212:149377188:Osteoblasts;chr3:149245536:149334284:Fetal Muscle;chr3:149246067:149274411:HMEC;chr3:149248327:149340070:HSMMtube;chr3:149249990:149340241:u87;chr3:149251990:149274195:Astrocytes;chr3:149289875:149307448:Astrocytes;chr3:149289900:149307910:NHLF;chr3:149289997:149304831:NHEK;chr3:149290104:149307572:HMEC;chr3:149290708:149324824:Skeletal Muscle Myoblast;chr3:149290708:149324889:HSMM;chr3:149334685:149378399:Aorta;chr3:149351458:149379446:Stomach Smooth Muscle;chr3:149357660:149377280:HepG2

ASHG19SELNC2A100000378	5.5976	up	noncoding	ENST00000444210	noncoding	ENSG00000230733	AC092171.4	Reliable	3251	chr7:5515435-5519442:+	chr7:5387467:5498044:Pancreatic islets;chr7:5397267:5470493:CD34 Primary RO01536;chr7:5420053:5470212:CD34 Primary RO01549;chr7:5420083:5482855:CD14;chr7:5424510:5470587:Colon Crypt 3;chr7:5434456:5470607:Colon Crypt 2;chr7:5434488:5470641:Colon Crypt 1;chr7:5434559:5470799:HCT-116;chr7:5435840:5470621:Sigmoid Colon;chr7:5435847:5470623:Brain Anterior Caudate;chr7:5435875:5470729:Lung;chr7:5435973:5470680:Esophagus;chr7:5435995:5470579:Gastric;chr7:5436159:5480069:VACO 400;chr7:5446568:5470510:Ovary;chr7:5446579:5470318:Aorta;chr7:5450705:5471544:Panc1;chr7:5452374:5498183:Brain Hippocampus Middle;chr7:5452391:5482021:Spleen;chr7:5456781:5470293:Duodenum Smooth Muscle;chr7:5456797:5470794:Stomach Smooth Muscle;chr7:5456932:5470951:Fetal Intestine Large;chr7:5456956:5470903:Brain Inferior Temporal Lobe;chr7:5456988:5470596:Right Atrium;chr7:5457003:5470597:Small Intestine;chr7:5457008:5470544:VACO 503;chr7:5457011:5470824:Brain Cingulate Gyrus;chr7:5457048:5470208:Brain Hippocampus Middle 150;chr7:5457056:5470456:Psoas Muscle;chr7:5457070:5470750:Fetal Intestine;chr7:5457083:5470669:CD34 Primary RO01480;chr7:5457084:5478285:Left

ASHG19SELNC2A100010217	2.0799	up	noncoding	ENCT000004304 94	noncoding	CATG00000100853	CATG00000100853.1	1691	chr8:129154492-129156182:+	chr8:129147244:129191727:MCF-7;chr8:129153748:129219173:HCC1954;chr8:129158962:129219148:Ly3;chr8:129159080:129218964:HBL1;chr8:129163751:129203247:HSMMtube;chr8:129165089:129210112:u87;chr8:129179300:129198539:HUVEC;chr8:129179447:129215108:Osteoblasts;chr8:129179643:129210014:Skeletal Muscle Myoblast;chr8:129179643:129210119:HSMM;chr8:129179715:129198151:NHEK;chr8:129179902:129198300:NHDF-Ad;chr8:129179996:129198145:HMEC;chr8:129180089:129198014:Astrocytes
ASHG19SELNC2A100014690	6.697	down	noncoding	FTMT26000002861	noncoding	CATG00000023598	CATG00000023598.1	1223	chr15:74249438-74250660:+	chr15:74206556:74230296:LNCaP;chr15:74213348:74225141:u87;chr15:74213376:74258891:Osteoblasts;chr15:74213387:74247399:Aorta;chr15:74213452:74250776:NHDF-Ad;chr15:74213638:74234284:IMR90;chr15:74214230:74282055:Pancreatic islets;chr15:74214460:74249890:NHLF;chr15:74214625:74258665:HSMMtube;chr15:74215187:74249875:Astrocytes;chr15:74215781:74257985:HSMM;chr15:74215847:74257892:Skeletal Muscle Myoblast;chr15:74242859:74301215:Tonsil;chr15:74243131:74300516:Ly3;chr15:74272874:74305861:Adipose Nuclei;chr15:74273370:74285288:HSMMtube;chr15:74273452:74305486:Stomach Smooth Muscle;chr15:74273515:74290001:Fetal Muscle;chr15:74273575:74285697:Left Ventricle;chr15:74273682:74344709:Spleen;chr15:74273688:74304323:Esophagus;chr15:74273721:74318349:Lung;chr15:74273767:74304358:Small Intestine;chr15:74273774:74285497:Right Atrium;chr15:74273803:74304414:Sigmoid Colon;chr15:74273819:74304427:Gastric;chr15:74273850:74300796:Thymus

ASHG19SELNC2A100017499	3.1319	up	noncoding	ENST0000050792 6	noncoding	ENSG00000248605	CTD-2306M5.1		369	chr5:25415755-25446016:-	chr5:25443805:25449125:H1
ASHG19SELNC2A101371129	3.1341	down	noncoding	NR_125796	noncoding	ENSG00000251003	RP11-152P17.2	Gold	1011	chr8:106792638-107072731:-	chr8:107056599:107074501:u87;chr8:107057040:107074578:Astrocytes
ASHG19SELNC2A100021876	2.2022	up	noncoding	ENST0000060208 3	noncoding	ENSG00000269371	CTD-2207O23.11	Reliable	293	chr19:7584802-7585346:-	chr19:7585325:7611185:Spleen

ASHG19SELNC2A100018732	4.0753	down	noncoding	ENST00000412754	noncoding	ENSG00000236305	RP11-126L15.4		369	chr7:100434936-100450238:-	chr7:100398301:100435555:Spleen;chr7:100398420:100435525:Adrenal Gland;chr7:100398433:100434682:VACO 400
ASHG19SELNC2A100017013	2.4806	down	noncoding	ENST00000605586	noncoding	ENSG00000271208	RP1-142L7.8	Reliable	594	chr6:112555366-112555959:-	chr6:112420456:112652968:Adipose Nuclei;chr6:112470164:112485340:Ly4;chr6:112500019:112620911:Stomach Smooth Muscle;chr6:112509677:112576958:Osteoblasts;chr6:112514688:112620667:Left Ventricle;chr6:112519021:112620635:Lung;chr6:112522254:112559801:u87;chr6:112523069:112576655:HUVEC;chr6:112523347:112576763:Duodenum Smooth Muscle;chr6:112523670:112576170:NHLF;chr6:112523883:112620772:Fetal Muscle;chr6:112538109:112590572:Right Atrium;chr6:112541266:112576548:NHDF-Ad;chr6:112589629:112633990:u87

ASHG19SELNC2A100004584	2.3341	up	noncoding	ENST000005381 13	noncoding	ENSG00000257042	RP11-993B23.3		792	chr12:28111450-28122746:+	chr12:28117440:28153947:HMEC;chr12:28117805:28153668:NHEK

ASHG19SELNC2A100009069	2.4862	up	noncoding	ENST00000512200	noncoding	ENSG00000250508	RP11-757G1.6	3252	chr11:68638132-68642010:+	chr11:68572105:68625963:LNCaP;chr11:68577606:68637837:Colon Crypt 1;chr11:68577679:68625778:Colon Crypt 2;chr11:68577695:68637841:Colon Crypt 3;chr11:68579709:68625996:Sigmoid Colon;chr11:68579710:68625902:Small Intestine;chr11:68584405:68611583:CD34 Primary RO01536;chr11:68592374:68626326:Fetal Intestine Large;chr11:68592393:68645914:Fetal Intestine;chr11:68592674:68623405:Gastric;chr11:68595052:68623717:Lung;chr11:68595181:68623887:Spleen;chr11:68600031:68623081:Adrenal Gland;chr11:68604978:68623116:Pancreas
ASHG19SELNC2A100665124	2.7941	down	noncoding	FTMT20800005640	noncoding	CATG00000043882	CATG00000043882.1	3773	chr2:98368166-98371938:+	chr2:98326092:98361442:CD4p CD25- CD45RAp Naive;chr2:98326429:98345268:Jurkat;chr2:98326885:98363002:CD4p CD25- Il17- PMAstim Th;chr2:98326948:98359916:CD4p CD25- Il17p PMAstim Th17;chr2:98328532:98359877:CD4p CD25- CD45ROp Memory;chr2:98328558:98363442:CD8 primiary;chr2:98328565:98379215:CD56

ASHG19SELNC2A100008453	2.4837	up	noncoding	ENST00000590513	noncoding	ENSG00000266962	RP11-400F19.6	Reliable	2311	chr17:40704456-40706766:-	chr17:40658405:40684432:Ovary;chr17:40660248:40690755:Aorta;chr17:40662787:40684718:Adipose Nuclei;chr17:40663804:40684677:Lung;chr17:40663811:40707842:Fetal Intestine Large;chr17:40663838:40684603:Left Ventricle;chr17:40663902:40684665:Right Atrium;chr17:40663925:40684645:Adrenal Gland;chr17:40663951:40684552:Stomach Smooth Muscle;chr17:40663953:40684495:Bladder;chr17:40663957:40709176:Brain Cingulate Gyrus;chr17:40663974:40708609:Brain Anterior Caudate;chr17:40663987:40708660:Brain Hippocampus Middle 150;chr17:40664061:40684631:Spleen;chr17:40664121:40675620:IMR90;chr17:40664744:40676005:Duodenum Smooth Muscle;chr17:40665081:40709281:Brain Inferior Temporal Lobe;chr17:40669175:40679696:Osteoblasts;chr17:40669185:40676001:Fetal Intestine;chr17:40669227:40707851:Fetal Muscle;chr17:40669248:40684649:u87;chr17:40669252:40684812:HUVEC;chr17:40669389:40675564:NHDF-Ad;chr17:40669427:40674802:Astrocytes;chr17:40669636:40674241:HSMM;chr17:40669650:40674227:Skeletal Muscle Myoblast;chr17:40669659:40678561:NHLF;chr17:40669779:40707582:Colon Crypt 3;chr17:40670048:40707606:VACO
ASHG19SELNC2A100012410	2.0561	down	noncoding	uc001voi.1	noncoding	AK123584	AK123584		2796	chr13:100143729-100148992:-	chr13:100136209:100160564:Spleen

ASHG19SELNC2A100015043	3.929	up	noncoding	ENST00000439423	noncoding	ENSG00000231010	RP6-109B7.2		1181	chr22:46451620-46454040:-	chr22:46435861:46436015:Brain Hippocampus Middle;chr22:46436214:46442955:Esophagus;chr22:46436313:46491627:Adipose Nuclei;chr22:46438115:46491495:Brain Hippocampus Middle 150;chr22:46445235:46456789:Brain Inferior Temporal Lobe;chr22:46445301:46456148:Brain Cingulate Gyrus;chr22:46445359:46455879:Brain Anterior Caudate;chr22:46445515:46454339:Lung;chr22:46445530:46452765:Sigmoid Colon;chr22:46445533:46455726:Esophagus;chr22:46445547:46452979:Gastric;chr22:46445554:46455707:Spleen;chr22:46445556:46455580:Right Atrium;chr22:46445577:46455681:Left Ventricle;chr22:46445655:46452991:Adrenal Gland;chr22:46445832:46455465:Tonsil;chr22:46447381:46491318:HSMMtube;chr22:46447605:46466955:Panc1;chr22:46447789:46491244:Osteoblasts;chr22:46447835:46473232:HCC1954;chr22:46447871:46452600:LNCaP;chr22:46447891:46457293:NHDF-Ad;chr22:46447955:46455571:NHLF;chr22:46448058:46467049:HMEC;chr22:46448292:46457565:u87;chr22:46451541:46485670:Bladder;chr22:46451675:46485305:VACO 9m;chr22:46451678:46486798:Right Ventricle;chr22:46451711:46485813:Colon Crypt 1;chr22:46451926:46469557:CD34 Primary RO01549;chr22:46452808:46469830:Ly3;chr22:46453193:46473350:CD34 Primary
ASHG19SELNC2A100000592	3.8019	up	noncoding	ENST00000508664	noncoding	ENSG00000251603	RP11-164P12.4	Reliable	304	chr4:152588376-152591654:+	chr4:152586171:152617153:Right Atrium

ASHG19SELNC2A100389223	2.7783	up	noncoding	MICT000002100059	noncoding	CATG00000051936	CATG00000051936.1	5242	chr2:235156750-235199271:-	chr2:235147238:235176281:Panc1;chr2:235149098:235171441:HMEC;chr2:235149154:235169603:u87;chr2:235149154:235171269:NHEK;chr2:235149185:235167891:Astrocytes;chr2:235149305:235168785:Osteoblasts;chr2:235149781:235169008:VACO 9m;chr2:235152068:235210364:DND41;chr2:235175304:235203223:Jurkat;chr2:235180344:235222848:CD56;chr2:235180686:235222778:CD8 primiary;chr2:235181359:235203307:CD4p CD25- Il17p PMAstim Th17;chr2:235187186:235203787:CD4 Naive Primary 8pool;chr2:235196775:235218846:CD4p CD25- CD45ROp Memory;chr2:235196776:235219293:CD4p CD25- Il17- PMAstim Th;chr2:235196855:235233139:CD4p CD25- CD45RAp Naive;chr2:235197112:235218282:CD4p CD225int CD127p Tmem;chr2:235197115:235203529:CD4 Memory Primary 7pool;chr2:235197255:235218330:CD3;chr2:235197260:235203385:CD8 Memory 7pool;chr2:235197417:235203263:CD8 Naive 8pool;chr2:235197495:235202520:CD8 Naive 7pool;chr2:235197813:235203468:CD4 Naive Primary 7pool

ASHG19SELNC2A100016215	12.237	up	noncoding	ENST00000607794	noncoding	ENSG00000272079	LA16c-380H5.5	Reliable	1266	chr16:3056121-3057386:+	chr16:3054493:3060932:Fetal Intestine;chr16:3054567:3061001:Fetal Intestine Large;chr16:3056482:3059299:H1
ASHG19SELNC2A100009233	2.0943	up	noncoding	ENST00000602760	noncoding	ENSG00000270019	RP11-141B14.1		1548	chr2:114645780-114647327:-	chr2:114611623:114730851:CD20;chr2:114615840:114655649:Tonsil;chr2:114633162:114690904:CD4p CD25- Il17- PMAstim Th;chr2:114634615:114682279:CD14
ASHG19SELNC2A100010774	2.7739	up	noncoding	ENST00000579368	noncoding	ENSG00000265962	RP11-674N23.1		818	chr18:8695854-8707619:-	chr18:8702720:8802804:Stomach Smooth Muscle;chr18:8704022:8758139:Ly1;chr18:8727827:8752563:HUVEC;chr18:8744562:8767510:NHDF-Ad;chr18:8745183:8753936:IMR90;chr18:8777013:8815112:HUVEC;chr18:8793031:8814089:NHDF-Ad;chr18:8793964:8814684:Astrocytes;chr18:8794127:8813331:NHLF;chr18:8796711:8814572:IMR90;chr18:8798740:8802517:HSMM;chr18:8798876:8802432:Skeletal Muscle Myoblast

ASHG19SELNC2A100005309	9.6877	down	noncoding	ENST00000550319	noncoding	ENSG00000258096	RP11-474P2.2	Reliable	627	chr12:46765246-46767561:+	chr12:46743309:46786096:Adipose Nuclei;chr12:46750690:46781406:HMEC;chr12:46750696:46801411:Osteoblasts;chr12:46750818:46797247:NHEK;chr12:46751076:46781994:Left Ventricle;chr12:46752905:46785767:CD20;chr12:46754023:46782918:CD14;chr12:46754278:46783404:CD19 Primary;chr12:46755152:46786747:Panc1;chr12:46758395:46797876:Duodenum Smooth Muscle;chr12:46758596:46785607:HUVEC;chr12:46758835:46779688:Brain Hippocampus Middle 150;chr12:46759700:46781118:CD4 Memory Primary 7pool;chr12:46760895:46797996:HeLa;chr12:46760905:46783619:NHDF-Ad;chr12:46760966:46798138:NHLF;chr12:46761022:46798920:Tonsil;chr12:46761111:46785610:Skeletal Muscle;chr12:46761122:46795854:Lung;chr12:46761154:46781460:CD34 Primary RO01536;chr12:46761219:46797045:Colon Crypt 1;chr12:46761973:46778834:CD34 Primary RO01480;chr12:46762301:46780693:HCT-116;chr12:46762605:46799048:IMR90;chr12:46762740:46799125:Astrocytes;chr12:46762818:46799053:HSMM;chr12:46763090:46784642:Colon Crypt 3;chr12:46763376:46781577:u87;chr12:46763621:46798940:Skeletal Muscle Myoblast;chr12:46763715:46785789:Toledo;chr12:46763765:46785105:Fetal
ASHG19SELNC2A100974679	2.315	up	noncoding	ENST00000500447	noncoding	ENSG00000247095	MIR210HG		1965	chr11:565660-568420:-	chr11:541055:566158:Colon Crypt 2;chr11:558473:564521:Colon Crypt 3

ASHG19SELNC2A100005830	2.6302	up	noncoding	ENST00000412838	noncoding	ENSG00000231073	RP11-316M1.3	Reliable	368	chr1:150945599-150948010:+	chr1:150940653:150952254:Adrenal Gland;chr1:150942608:150952428:HepG2;chr1:150943271:150956639:VACO 400;chr1:150943284:150956638:Pancreas;chr1:150944158:150952463:K562
ASHG19SELNC2A100008952	4.4434	down	noncoding	ENST00000432133	noncoding	ENSG00000235321	AC007556.3		307	chr2:169957253-169957720:+	chr2:169927476:169967083:HBL1
ASHG19SELNC2A101204393	2.3311	up	noncoding	NR_146191	noncoding	ENSG00000156831	NSMCE2		1379	chr8:126104065-126379367:+	chr8:126122373:126165172:NHEK;chr8:126122465:126166429:HCT-116;chr8:126130641:126189610:Fetal Intestine;chr8:126130794:126189662:Fetal Intestine Large;chr8:126180450:126252726:HCT-116;chr8:126224966:126243260:NHEK;chr8:126225015:126245341:HMEC;chr8:126230124:126245806:CD14
ASHG19SELNC2A101204393	2.3311	up	noncoding	NR_146191	noncoding	ENSG00000156831	NSMCE2		1379	chr8:126104065-126379367:+	chr8:126270470:126321395:HCT-116;chr8:126274580:126316300:MM1S;chr8:126285860:126307851:Jurkat

ASHG19SELNC2A100017024	2.9057	up	noncoding	ENST00000607419	noncoding	ENSG00000271952	RP11-245G13.2	461	chr2:11018395-11025244:+	chr2:10983264:11018807:Left Ventricle;chr2:10983310:11020167:Right Atrium

ASHG19SELNC2A100003658	2.0888	down	noncoding	ENST00000437416	noncoding	ENSG00000233384	RP11-100E13.1	Gold	403	chr1:224802998-224803922:-	chr1:224803044:224840981:u87
ASHG19SELNC2A100187807	2.2247	up	noncoding	ENST00000425008	noncoding	ENSG00000233885	YEATS2-AS1	Reliable	2442	chr3:183524245-183527303:-	chr3:183482390:183547547:Brain Inferior Temporal Lobe;chr3:183483604:183547230:Brain Cingulate Gyrus;chr3:183492820:183545105:Brain Hippocampus Middle 150;chr3:183507731:183546393:Brain Angular Gyrus;chr3:183511231:183546673:Brain Hippocampus Middle;chr3:183511582:183541262:Brain Anterior Caudate;chr3:183518411:183538120:Brain Mid Frontal Lobe

ASHG19SELNC2A100018490	2.0122	up	noncoding	ENST00000607353	noncoding	ENSG00000272369	RP11-446N19.1	Reliable	448	chr12:46931285-47046333:+	chr12:46909532:46955601:MM1S;chr12:46916854:46968424:HSMMtube;chr12:46940092:46957831:HMEC;chr12:46940146:46956777:NHLF;chr12:46940192:46957464:NHEK;chr12:46940196:46952685:IMR90;chr12:46940207:46957640:Osteoblasts;chr12:46940240:46958128:u87;chr12:46940255:46958244:HeLa
ASHG19SELNC2A100017561	2.1954	up	noncoding	ENST00000497872	noncoding	ENSG00000253701	AL928768.3		359	chr14:106170301-106170939:-	chr14:106134703:106178820:Toledo;chr14:106143072:106180530:CD20;chr14:106144201:106215700:CD19 Primary;chr14:106144863:106176282:GM12878;chr14:106145079:106173688:MM1S;chr14:106145131:106177266:CD56;chr14:106145141:106176987:Sigmoid Colon;chr14:106145254:106173704:Spleen;chr14:106145575:106171606:H2171;chr14:106145712:106176054:Small Intestine;chr14:106146139:106178977:Tonsil;chr14:106146296:106177340:DHL6;chr14:106148019:106169399:Ly4
ASHG19SELNC2A100012187	3.0687	up	noncoding	ENST00000500009	noncoding	ENSG00000246375	RP11-10L7.1		1654	chr4:89206094-89252573:+	chr4:89194528:89207593:CD20;chr4:89202585:89207138:CD4 Memory Primary 7pool;chr4:89203005:89207053:CD8 Naive 7pool;chr4:89203229:89206883:CD4 Naive Primary 7pool;chr4:89203439:89207065:Fetal Muscle;chr4:89203835:89219912:Lung

ASHG19SELNC2A100062160	3.2198	up	noncoding	NR_126339	noncoding	ENSG00000253320	KB-1507C5.2	Gold	807	chr8:103876499-103990104:+	chr8:103898697:103923636:RPMI-8402
ASHG19SELNC2A100015521	3.7702	down	noncoding	FTMT22400001414	noncoding	CATG00000082908	CATG00000082908.1		1132	chr6:14777961-14779092:+	chr6:14777649:14799417:H1

ASHG19SELNC2A100429611	3.0814	down	noncoding	ENST00000542598	noncoding	ENSG00000256448	RP11-809N8.4	334	chr11:73116342-73121717:+	chr11:72756687:72901663:CD20;chr11:72846374:72871204:Ly3;chr11:72846686:72901203:Spleen;chr11:72847687:72869983:DHL6;chr11:72848179:72871515:Tonsil;chr11:72848285:72870613:Toledo;chr11:72848432:72901825:Ly4;chr11:72848919:72865794:CD34 Primary RO01480;chr11:72849766:72866057:CD34 Primary RO01536;chr11:72849904:72901275:Ly1;chr11:72849937:72901910:HBL1;chr11:72850008:72869679:Fetal Muscle;chr11:72850207:72870983:GM12878;chr11:72850244:72889794:HSMMtube;chr11:72850428:72870499:MM1S;chr11:72850431:72887707:Fetal Intestine Large;chr11:72850513:72872020:CD19 Primary;chr11:72850594:72900096:Left Ventricle;chr11:72851228:72894684:Lung;chr11:72851301:72869995:Sigmoid Colon;chr11:72851423:72870073:Aorta;chr11:72852098:72865986:Thymus;chr11:72857024:72894861:Right Atrium;chr11:72857798:72889632:Right Ventricle;chr11:72888015:72952042:Esophagus;chr11:72895515:72954355:CD14;chr11:72895962:72913543:HCC1954;chr11:72916992:72952643:Psoas Muscle;chr11:72923727:72952810:Skeletal Muscle;chr11:72945387:73001156:Spleen;chr11:72967487:73001255:HSMMtube;chr11:72977978:73001095:Fetal Intestine Large;chr11:72998904:73072142:Osteoblasts;chr11:72999827:73046703:NHDF-Ad;chr11:73016977:73056710:Brain Hippocampus

ASHG19SELNC2A100009319	2.3673	down	noncoding	ENCT00000281389	noncoding	CATG00000058349	CATG00000058349.1	1343	chr22:27502558-27503900:-	chr22:27500962:27513287:Astrocytes;chr22:27503230:27513644:HUVEC;chr22:27507902:27532858:CD14
ASHG19SELNC2A100018000	2.7548	down	noncoding	ENST00000380722	noncoding	ENSG00000205562	RP11-497E19.1	1747	chr14:85991477-85996332:-	chr14:85982227:86003404:HUVEC;chr14:85982283:86003174:NHEK;chr14:85982527:86007566:Fetal Muscle;chr14:85992832:86002969:HSMM;chr14:85992877:86002960:Skeletal Muscle Myoblast

ASHG19SELNC2A100009362	6.1037	down	noncoding	FTMT23400002236	noncoding	CATG00000108059	CATG00000108059.1	1208	chr9:27598016-27599223:-	chr9:27329135:27504747:Brain Anterior Caudate;chr9:27329311:27370319:Brain Cingulate Gyrus;chr9:27332408:27370467:Brain Hippocampus Middle 150;chr9:27332486:27387544:Brain Inferior Temporal Lobe;chr9:27332721:27370542:Brain Hippocampus Middle;chr9:27333147:27418143:Fetal Intestine;chr9:27333300:27369656:Brain Angular Gyrus;chr9:27333419:27369924:Ovary;chr9:27337262:27368749:Brain Mid Frontal Lobe;chr9:27352664:27418216:Fetal Intestine Large;chr9:27385894:27461545:HMEC;chr9:27393290:27448314:Brain Hippocampus Middle 150;chr9:27393803:27548644:Brain Cingulate Gyrus;chr9:27394013:27435005:Brain Angular Gyrus;chr9:27394386:27448375:Brain Hippocampus Middle;chr9:27402236:27452194:Brain Inferior Temporal Lobe;chr9:27430855:27488425:Fetal Intestine Large;chr9:27430983:27478425:Fetal Intestine;chr9:27498149:27530621:NHEK;chr9:27527724:27603332:CD14

ASHG19SELNC2A100002397	3.7235	up	noncoding	ENST000006102 72	noncoding	ENSG00000272906	RP11-533E19.7	Reliable	989	chr1:179850742-179851730:-	chr1:179839958:179860126:CD20;chr1:179844669:179854441:NHEK;chr1:179849088:179853964:CD4 Memory Primary 7pool;chr1:179849314:179880318:CD34 Primary RO01536;chr1:179849391:179854038:CD8 Memory 7pool;chr1:179849924:179854643:CD4p CD25-CD45ROp Memory;chr1:179850026:179857966:CD4p CD25-Il17- PMAstim Th;chr1:179850177:179854496:CD8 primiary;chr1:179850202:179853726:CD4 Memory Primary 8pool;chr1:179850206:179854257:CD8 Naive 8pool;chr1:179850276:179853475:CD4 Naive Primary 7pool;chr1:179850280:179854565:CD56;chr1:179850291:179853582:CD8 Naive 7pool;chr1:179850344:179853874:CD3;chr1:179850363:179854530:CD4 Naive Primary 8pool;chr1:179850367:179857453:CD19 Primary;chr1:179850439:179854256:CD4p CD25-Il17p PMAstim Th17;chr1:179850474:179854145:CD4p CD225int CD127p Tmem
ASHG19SELNC2A100338406	2.214	down	noncoding	ENST00000558150	noncoding	ENSG00000259188	CTD-2647E9.3	Reliable	520	chr15:50172622-50175722:+	chr15:50123014:50159837:HeLa;chr15:50138719:50171586:Osteoblasts;chr15:50138790:50172005:IMR90;chr15:50139165:50171542:NHDF-Ad;chr15:50166048:50210873:Adipose Nuclei

ASHG19SELNC2A100020556	2.1895	up	noncoding	ENST00000610137	noncoding	ENSG00000273196	RP11-717A5.2	73	chr2:85614197-85614269:-	chr2:85610306:85674183:u87;chr2:85615286:85669864:CD14;chr2:85617835:85649228:DND41;chr2:85623138:85649311:Esophagus;chr2:85623502:85674409:Lung;chr2:85623516:85673793:Small Intestine;chr2:85631788:85678391:H2171;chr2:85632045:85666848:Panc1;chr2:85632068:85675124:HSMMtube;chr2:85632201:85674477:Sigmoid Colon;chr2:85635309:85675097:Brain Anterior Caudate;chr2:85635508:85675124:Brain Hippocampus Middle 150;chr2:85639655:85698966:HeLa;chr2:85639667:85673833:Gastric;chr2:85639748:85696795:HCT-116;chr2:85639942:85675051:Pancreatic islets;chr2:85640599:85666339:K562;chr2:85644628:85675281:Brain Hippocampus Middle;chr2:85644800:85648954:HepG2;chr2:85644802:85675152:Brain Inferior Temporal Lobe;chr2:85644821:85675289:Brain Cingulate Gyrus;chr2:85645069:85673833:Right Atrium;chr2:85645134:85674917:Brain Angular Gyrus;chr2:85645170:85676652:Pancreas;chr2:85645486:85665631:CD34 fetal;chr2:85645524:85674425:Brain Mid Frontal Lobe;chr2:85663480:85676787:Adrenal Gland;chr2:85664354:85665949:Adipose Tissue

ASHG19SUPERLNC2A100018928	2.373	down	noncoding	ENST00000487240	noncoding	ENSG00000242808	SOX2-OT	751	chr3:180707558-180775032:+

ASHG19SELNC2A100000822	3.0173	up	noncoding	HBMT00000484969	noncoding	CATG00000023105	CATG00000023105.1		1508	chr15:56286018-56287525:+	chr15:56283381:56299470:NHEK
ASHG19SELNC2A100560335	2.1483	down	noncoding	NR_110061	noncoding	ENSG00000244302	PEX5L-AS2	Gold	617	chr3:179617325-179639687:+	chr3:179638636:179710163:Brain Inferior Temporal Lobe;chr3:179638756:179704024:Brain Cingulate Gyrus;chr3:179641103:179698343:Ly1;chr3:179644834:179696126:DND41;chr3:179645438:179704091:Brain Hippocampus Middle 150;chr3:179645611:179703944:Brain Hippocampus Middle;chr3:179645746:179703644:Brain Anterior Caudate;chr3:179645972:179700720:Brain Angular Gyrus;chr3:179663448:179692843:Jurkat
ASHG19SELNC2A100688584	4.7528	down	noncoding	ENST00000420776	noncoding	ENSG00000229388	RP11-442N24__B.1	Gold	624	chr1:28969740-28975042:+	chr1:28968395:28971700:K562
ASHG19SELNC2A100015410	4.0422	up	noncoding	ENST00000607740	noncoding	ENSG00000271916	RP11-884K10.6	Reliable	256	chr3:53831791-53832046:-	chr3:53766774:53881421:Pancreatic islets;chr3:53801318:53810069:HBL1;chr3:53874893:53881624:HepG2

ASHG19SELNC2A100000660	2.6384	up	noncoding	ENST000005307 5	noncoding	ENSG00000257258	RP11-946L16.1	Reliable	593	chr12:29296129-29301608:-	chr12:29300482:29319315:CD34 Primary RO01549;chr12:29300528:29319925:CD34 Primary RO01536;chr12:29300531:29320008:CD34 Primary RO01480;chr12:29300779:29330669:CD14;chr12:29301255:29303417:Colon Crypt 2

ASHG19SELNC2A101025937	2.4752	up	noncoding	NR_120365	noncoding	ENSG00000259343	RP11-761I4.3	Gold	1077	chr15:81616674-81735417:+	chr15:81560841:81617674:CD4p CD25- CD45RAp Naive;chr15:81567470:81610802:Tonsil;chr15:81567732:81610103:CD4p CD25- Il17- PMAstim Th;chr15:81568447:81607720:Thymus;chr15:81575890:81617762:CD20;chr15:81576184:81624979:MM1S;chr15:81576243:81605254:Toledo;chr15:81576278:81610588:CD19 Primary;chr15:81576315:81609224:CD8 primiary;chr15:81576349:81607275:CD14;chr15:81576351:81605600:Ly1;chr15:81576441:81608661:Spleen;chr15:81576460:81609122:CD8 Naive 7pool;chr15:81576519:81615707:Sigmoid Colon;chr15:81576534:81615697:Small Intestine;chr15:81576723:81609203:CD56;chr15:81576780:81608956:CD8 Naive 8pool;chr15:81577272:81604284:CD4p CD25- Il17p PMAstim Th17;chr15:81577407:81600449:CD4p CD25- CD45ROp Memory;chr15:81577413:81604746:CD4 Naive Primary 7pool;chr15:81577667:81605046:Fetal Thymus;chr15:81577957:81599266:CD4 Memory Primary 7pool;chr15:81578040:81598967:CD3;chr15:81578270:81597142:CD4p CD225int CD127p Tmem;chr15:81578540:81596395:CD8 Memory 7pool;chr15:81578571:81608318:CD4 Naive Primary 8pool;chr15:81583158:81611021:Ly3;chr15:81583334:81595850:CD4 Memory Primary 8pool;chr15:81595978:81631354:NHDF-Ad

ASHG19SELNC2A100019746	2.4916	down	noncoding	ENST00000429512	noncoding	ENSG00000228235	AP001476.4	415	chr21:47472510-47473019:+	chr21:47368269:47414613:Aorta;chr21:47368978:47399878:Fetal Muscle;chr21:47370640:47418875:Stomach Smooth Muscle;chr21:47373022:47488156:Ovary;chr21:47373188:47425562:NHDF-Ad;chr21:47373962:47413579:Lung;chr21:47390790:47421807:Esophagus;chr21:47390883:47405694:Bladder;chr21:47390890:47405005:NHLF;chr21:47441142:47554313:NHDF-Ad;chr21:47441234:47488057:Sigmoid Colon;chr21:47441247:47488321:Fetal Muscle;chr21:47441257:47531436:Esophagus;chr21:47444324:47564240:Osteoblasts;chr21:47444870:47513373:Astrocytes;chr21:47447910:47520896:NHLF;chr21:47457705:47513717:u87;chr21:47458019:47488324:Aorta;chr21:47468502:47488351:Stomach Smooth Muscle;chr21:47468608:47520268:IMR90;chr21:47508616:47554180:Stomach Smooth Muscle
ASHG19SELNC2A100015769	2.4073	down	noncoding	ENST00000435287	noncoding	ENSG00000227220	RP11-69I8.3	495	chr6:132272086-132398533:+	chr6:132267550:132277507:HUVEC
ASHG19SELNC2A100015769	2.4073	down	noncoding	ENST00000435287	noncoding	ENSG00000227220	RP11-69I8.3	495	chr6:132272086-132398533:+	chr6:132380411:132409826:HUVEC

ASHG19SELNC2A100009973	2.5952	down	noncoding	ENST00000558061	noncoding	ENSG00000259705	RP11-227D13.1	Reliable	1943	chr15:48938148-48944213:+	chr15:48826879:48877845:Fetal Muscle;chr15:48827549:48879734:Adipose Nuclei;chr15:48827729:48939777:Osteoblasts;chr15:48827840:48895741:HSMMtube;chr15:48827922:48886063:HSMM;chr15:48828108:48860209:Skeletal Muscle Myoblast;chr15:48828210:48897197:NHDF-Ad;chr15:48829461:48896062:NHLF;chr15:48893707:48965905:Adipose Nuclei;chr15:48914213:48939235:NHDF-Ad;chr15:48919316:48938986:Astrocytes;chr15:48958694:48996557:Osteoblasts;chr15:48959207:49018161:u87;chr15:48959692:48988676:Astrocytes
ASHG19SELNC2A101296549	2.1039	up	noncoding	NR_136182	noncoding	ENSG00000138092	CENPO		4234	chr2:25015984-25045245:+	chr2:25013117:25042062:K562
ASHG19SELNC2A100018341	2.0557	up	noncoding	ENST00000518302	noncoding	ENSG00000254060	RP11-172E10.1	Gold	545	chr8:81949199-81953066:+	chr8:81933177:81982485:HSMMtube;chr8:81933554:81964092:HSMM;chr8:81933648:81990327:u87;chr8:81933714:81949507:Skeletal Muscle Myoblast;chr8:81972164:82025418:CD4p CD25- Il17- PMAstim Th;chr8:81972743:82025379:Tonsil;chr8:81972782:82025268:CD4p CD25- Il17p PMAstim Th17;chr8:81973555:82025340:CD8 primiary;chr8:81976027:82025174:DHL6;chr8:81976630:82025097:Ly1;chr8:81992109:82025086:CD4 Naive Primary 8pool;chr8:82004756:82067740:DND41;chr8:82004923:82066597:Jurkat;chr8:82005108:82074527:Spleen;chr8:82039455:82074688:CD14

ASHG19SELNC2A100012411	3.4038	down	noncoding	uc004ags.1	noncoding	AK130904	AK130904	Reliable	3865	chr9:71150806-71154670:+	chr9:71031513:71125151:Stomach Smooth Muscle;chr9:71031835:71124316:Right Atrium;chr9:71050822:71176451:Duodenum Smooth Muscle;chr9:71120258:71181035:CD14
ASHG19SELNC2A100005405	6.1464	down	noncoding	ENST00000597609	noncoding	ENSG00000269292	CTB-12A17.3	Reliable	296	chr19:47112534-47114036:-	chr19:47103218:47109496:Pancreatic islets;chr19:47103267:47107798:Colon Crypt 1;chr19:47103338:47120213:Colon Crypt 2;chr19:47103442:47123428:CD4 Naive Primary 7pool;chr19:47122887:47130350:Aorta
ASHG19SELNC2A100012387	2.846	up	noncoding	ENST00000609247	noncoding	ENSG00000273204	RP4-549L20.3	Reliable	429	chr1:101360484-101360912:+	chr1:101359021:101363256:HeLa;chr1:101359538:101363446:CD8 primiary
ASHG19SELNC2A100002934	2.4457	down	noncoding	ENST00000473893	noncoding	ENSG00000242012	RP11-338L18.1	Gold	328	chr3:182082935-182086044:+	chr3:182085910:182115627:MM1S

ASHG19SELNC2A100013008	2.2443	up	noncoding	ENST00000533552	noncoding	ENSG00000255008	AP000442.4	Reliable	769	chr11:59328619-59333547:-	chr11:59303935:59334687:CD8 Memory 7pool;chr11:59304004:59334736:CD4 Memory Primary 7pool;chr11:59304097:59334448:CD34 Primary RO01536;chr11:59304100:59334146:K562;chr11:59316606:59334199:Fetal Muscle
ASHG19SUPERLNC2A100007718	2.7376	down	noncoding	ENST00000499203	noncoding	ENSG00000245146	LINC01024		2769	chr5:139482507-139487228:-	

ASHG19SELNC2A100009314	3.1419	up	noncoding	FTMT20800013964	noncoding	CATG00000047093	CATG00000047093.1	2494	chr2:235216857-235219350:+	chr2:235147238:235176281:Panc1;chr2:235149098:235171441:HMEC;chr2:235149154:235169603:u87;chr2:235149154:235171269:NHEK;chr2:235149185:235167891:Astrocytes;chr2:235149305:235168785:Osteoblasts;chr2:235149781:235169008:VACO 9m;chr2:235152068:235210364:DND41;chr2:235175304:235203223:Jurkat;chr2:235180344:235222848:CD56;chr2:235180686:235222778:CD8 primiary;chr2:235181359:235203307:CD4p CD25- Il17p PMAstim Th17;chr2:235187186:235203787:CD4 Naive Primary 8pool;chr2:235196775:235218846:CD4p CD25- CD45ROp Memory;chr2:235196776:235219293:CD4p CD25- Il17- PMAstim Th;chr2:235196855:235233139:CD4p CD25- CD45RAp Naive;chr2:235197112:235218282:CD4p CD225int CD127p Tmem;chr2:235197115:235203529:CD4 Memory Primary 7pool;chr2:235197255:235218330:CD3;chr2:235197260:235203385:CD8 Memory 7pool;chr2:235197417:235203263:CD8 Naive 8pool;chr2:235197495:235202520:CD8 Naive 7pool;chr2:235197813:235203468:CD4 Naive Primary 7pool
ASHG19SELNC2A100012186	3.1388	up	noncoding	ENST00000491676	noncoding	ENSG00000242522	KLHL6-AS1	503	chr3:183266523-183270114:+	chr3:183200971:183274916:MM1S;chr3:183212304:183275431:CD4p CD25- Il17- PMAstim Th;chr3:183220239:183279244:Ly1;chr3:183220552:183274601:Toledo;chr3:183222014:183274594:DHL6;chr3:183224578:183274663:CD14;chr3:183229085:183274978:CD20;chr3:183229386:183311073:Tonsil;chr3:183249585:183272849:Fetal Intestine Large;chr3:183251026:183276917:CD34 Primary RO01536

ASHG19SELNC2A100571005	2.2525	down	noncoding	ENST000005991 27	noncoding	ENSG00000269729	AC006262.4	Reliable	612	chr19:46667150-46683904:-	chr19:46683563:46726215:HMEC;chr19:46702145:46712947:VACO 9m	

ASHG19SELNC2A100270769	2.034	up	noncoding	ENST00000452922	noncoding	ENSG00000224081	LINC01057	1331	chr1:95123089-95285837:-	chr1:95063039:95125029:VACO503;chr1:95067475:95121313:HCC1954;chr1:95082138:95090228:HMEC;chr1:95082362:95127570:HeLa;chr1:95086964:95115430:IMR90;chr1:95106865:95124196:VACO 9m

ASHG19SELNC2A100270769	2.034	up	noncoding	ENST00000452922	noncoding	ENSG00000224081	LINC01057	1331	chr1:95123089-95285837:-	chr1:95166344:95203706:RPMI-8402;chr1:95171551:95220502:u87;chr1:95191016:95222575:IMR90

ASHG19SELNC2A100010088	2.6387	down	noncoding	ENST00000609697	noncoding	ENSG00000272789	RP11-286H15.1	Reliable	852	chr2:128383572-128384423:-	chr2:128331989:128410775:Toledo;chr2:128343496:128408767:DND41;chr2:128343598:128400582:Jurkat;chr2:128344401:128381109:RPMI-8402;chr2:128377348:128434025:Spleen;chr2:128387883:128439505:Left Ventricle;chr2:128388413:128432789:Lung;chr2:128388560:128432873:Right Atrium;chr2:128389263:128442440:Stomach Smooth Muscle;chr2:128390495:128432769:Aorta;chr2:128394646:128433940:Brain Hippocampus Middle;chr2:128394715:128434019:Esophagus;chr2:128408605:128433645:Right Ventricle
ASHG19SELNC2A100894159	2.1788	up	noncoding	MICT00000219191	noncoding	CATG00000053438	CATG00000053438.1		14983	chr20:46653963-46696591:+	chr20:46691877:46711896:MCF-7

ASHG19SELNC2A100004629	2.1792	up	noncoding	ENST00000563225	noncoding	ENSG00000261211	RP1-80N2.3	Reliable	3325	chr6:6680542-6683866:-	chr6:6579291:6611287:CD14;chr6:6580795:6617577:Thymus;chr6:6583756:6594187:GM12878;chr6:6584625:6612024:Ly4;chr6:6584637:6621563:CD20;chr6:6586207:6612284:CD19 Primary;chr6:6618133:6786846:Adipose Nuclei;chr6:6654590:6705900:H2171;chr6:6676758:6729396:CD14;chr6:6676855:6735940:HeLa;chr6:6677167:6726548:Sigmoid Colon;chr6:6677214:6759788:Small Intestine;chr6:6677223:6760384:Fetal Muscle;chr6:6677228:6760155:Lung;chr6:6677240:6726479:Colon Crypt 3;chr6:6677241:6752818:Right Atrium;chr6:6677249:6726130:Colon Crypt 1;chr6:6677359:6760016:Colon Crypt 2;chr6:6677932:6725877:NHEK;chr6:6677946:6726782:Duodenum Smooth Muscle;chr6:6677976:6754403:Fetal Intestine;chr6:6678003:6726489:Esophagus;chr6:6678285:6760335:Gastric;chr6:6678777:6754397:Fetal Intestine Large;chr6:6684521:6725182:CD34 fetal;chr6:6689425:6753586:HSMMtube;chr6:6696674:6725727:DND41;chr6:6698514:6725690:RPMI-8402;chr6:6698606:6713749:Jurkat;chr6:6700517:6725237:CD34 adult;chr6:6717838:6752019:Left Ventricle;chr6:6723261:6767064:Psoas Muscle;chr6:6724345:6752510:Adrenal Gland;chr6:6781001:6827603:HeLa;chr6:6790127:6827876:Esophagus;chr6:6802457:6822261:HMEC

ASHG19SELNC2A100561896	2.461	up	noncoding	ENST0000051262 4	noncoding	ENSG00000249345	RP11-575F12.1	1344	chr12:127399766-127544942:-	chr12:127410071:127461787:RPMI-8402
ASHG19SELNC2A100017707	2.1197	up	noncoding	ENST0000060415 7	noncoding	ENSG00000268471	MIR4453	3000	chr4:153457416-153460415:+	chr4:153422084:153483950:GM12878;chr4:153454258:153478261:CD4p CD25- Il17- PMAstim Th;chr4:153454972:153459787:CD4 Memory Primary 7pool;chr4:153455385:153460256:CD4p CD25- Il17p PMAstim Th17;chr4:153455617:153459458:CD4 Naive Primary 7pool;chr4:153455976:153459423:Colon Crypt 3

ASHG19SELNC2A100013910	2.4645	up	noncoding	ENST00000602982	noncoding	ENSG00000269961	CTD-2033C11.1	Reliable	507	chr5:65220457-65220963:-	chr5:65207428:65227431:Fetal Intestine Large;chr5:65209341:65224899:Fetal Intestine

ASHG19SELNC2A100001968	2.7898	up	noncoding	ENST00000424518	noncoding	ENSG00000228630	HOTAIR	2421	chr12:54356092-54368740:-	chr12:54357641:54378730:Panc1;chr12:54365295:54381230:HSMMtube;chr12:54365669:54400290:Fetal Muscle;chr12:54365772:54383178:HeLa;chr12:54372269:54401036:Skeletal Muscle;chr12:54374456:54401235:Psoas Muscle;chr12:54378772:54424544:Panc1;chr12:54381997:54402772:NHDF-Ad;chr12:54383968:54409342:Adipose Nuclei;chr12:54402812:54428773:NHDF-Ad;chr12:54406197:54422033:Skeletal Muscle;chr12:54406332:54427917:Fetal Muscle;chr12:54406517:54423230:Psoas Muscle;chr12:54409713:54420978:NHEK;chr12:54410440:54427864:Adipose Nuclei;chr12:54410599:54425812:Adrenal Gland;chr12:54411076:54414317:Ovary

ASHG19SELNC2A100425354	3.1624	down	noncoding	NR_105018	noncoding	ENSG00000249061	RP11-72L22.1	Gold	1487	chr5:86415965-86543835:+	chr5:86393568:86437319:MM1S;chr5:86394511:86425151:HMEC;chr5:86407555:86451455:u87;chr5:86408318:86448368:Osteoblasts
ASHG19SELNC2A100003593	5.5871	up	noncoding	ENST00000420563	noncoding	ENSG00000229525	AC053503.4	Gold	1598	chr2:220253218-220268355:+	chr2:220261721:220319688:HSMMtube;chr2:220262508:220327202:Psoas Muscle;chr2:220262531:220327030:Skeletal Muscle;chr2:220266957:220275716:Stomach Smooth Muscle;chr2:220267752:220320006:Duodenum Smooth Muscle;chr2:220268155:220323436:Fetal Muscle;chr2:220268381:220340932:Left Ventricle;chr2:220268402:220331570:Esophagus;chr2:220268786:220327230:Lung;chr2:220268987:220289010:Right Ventricle;chr2:220278601:220294514:Stomach Smooth Muscle;chr2:220288406:220364010:Aorta;chr2:220294939:220335647:u87;chr2:220305298:220327943:Stomach Smooth Muscle;chr2:220305814:220319232:Right Ventricle

ASHG19SELNC2A100012378	2.7844	up	noncoding	ENST00000606924	noncoding	ENSG00000272341	RP1-151F17.2	Reliable	2538	chr6:16764577-16767114:+	chr6:16651465:16714539:CD4p CD25- Il17- PMAstim Th;chr6:16651709:16714493:CD4p CD25- Il17p PMAstim Th17;chr6:16659415:16770204:CD14;chr6:16668829:16770985:NHEK;chr6:16668888:16771136:HMEC;chr6:16670001:16771263:Spleen;chr6:16672019:16771851:Panc1;chr6:16674385:16784542:HSMMtube;chr6:16674509:16771483:Osteoblasts;chr6:16674745:16771289:HSMM;chr6:16674759:16771241:Skeletal Muscle Myoblast;chr6:16676542:16770991:u87;chr6:16697431:16771599:Duodenum Smooth Muscle;chr6:16698914:16771717:Stomach Smooth Muscle;chr6:16699036:16764567:Brain Anterior Caudate;chr6:16699517:16771184:Lung;chr6:16704167:16771389:Astrocytes;chr6:16731181:16764249:CD4p CD25- Il17p PMAstim Th17;chr6:16736801:16782756:Left Ventricle;chr6:16739584:16763862:Esophagus;chr6:16742449:16782423:Gastric;chr6:16752143:16771175:VACO 503;chr6:16758092:16765048:CD8 Memory 7pool;chr6:16758240:16764321:CD8 Naive 7pool;chr6:16758304:16770625:CD4p CD25- CD45ROp Memory;chr6:16758455:16764963:CD4 Memory Primary 7pool;chr6:16758493:16770384:CD4 Naive Primary 8pool;chr6:16758850:16770460:CD3;chr6:16758956:16764786:CD4p CD225int CD127p Tmem;chr6:16759123:16764973:CD4 Memory Primary 8pool

ASHG19SELNC2A100016204	3.5657	up	noncoding	ENST000006067 78	noncoding	ENSG00000271930	RP11-44N12.5	Reliable	504	chr8:99955823-99956326:+	chr8:99942276:99962147:Ovary;chr8:99948256:99963950:NHDF-Ad

ASHG19SELNC2A100002339	4.8772	down	noncoding	ENST00000579499	noncoding	ENSG00000263975	CTB-58E17.9	Reliable	563	chr17:36871581-36876525:-	chr17:36841483:36864135:Spleen;chr17:36849347:36863638:Left Ventricle;chr17:36849416:36867442:Fetal Muscle;chr17:36849427:36868217:Lung;chr17:36849443:36863581:Small Intestine;chr17:36849446:36869721:Right Atrium;chr17:36849486:36864348:Sigmoid Colon;chr17:36849658:36862895:Right Ventricle;chr17:36849707:36864129:Gastric;chr17:36852020:36863435:MM1S;chr17:36852687:36863936:Tonsil;chr17:36854835:36891315:CD4p CD25- Il17p PMAstim Th17;chr17:36854933:36891548:CD4p CD25- Il17- PMAstim Th;chr17:36856374:36863732:Ly3;chr17:36856433:36863910:CD56;chr17:36856888:36888783:CD8 primiary;chr17:36857029:36864323:Pancreatic islets;chr17:36857157:36863577:Esophagus;chr17:36857165:36863536:Adrenal Gland;chr17:36857173:36861522:K562;chr17:36857186:36861774:HeLa;chr17:36857283:36862851:Pancreas;chr17:36857283:36862900:VACO 9m;chr17:36857336:36862962:Thymus;chr17:36857431:36876540:VACO 503;chr17:36880595:36907474:Gastric;chr17:36880935:36900163:Colon Crypt 2;chr17:36881475:36907782:Small Intestine;chr17:36881476:36907973:Colon Crypt 1;chr17:36883977:36907951:Lung;chr17:36886485:36906657:Pancreas;chr17:36886615:36890751:LNCaP

ASHG19SELNC2A100761435	2.0668	up	noncoding	ENST00000449529	noncoding	ENSG00000152487	ARL5B-AS1		689	chr10:18943480-18948185:-	chr10:18945942:18951317:Fetal Intestine Large;chr10:18946186:18951042:Fetal Intestine;chr10:18946586:18950415:HepG2
ASHG19SELNC2A100002004	2.063	up	noncoding	ENST00000435306	noncoding	ENSG00000231154	MORF4L2-AS1		2086	chrX:102942212-102946700:+	chrX:102940451:102966372:Brain Hippocampus Middle 150;chrX:102940770:102967461:Brain Hippocampus Middle;chrX:102940814:102963203:Brain Inferior Temporal Lobe;chrX:102940926:102963060:Brain Anterior Caudate;chrX:102941044:102966244:Brain Cingulate Gyrus;chrX:102945975:102962814:Brain Angular Gyrus
ASHG19SELNC2A100571353	2.3203	up	noncoding	ENST00000606496	noncoding	ENSG00000272288	RP11-140K17.3	Reliable	365	chr6:34664722-34665247:+	chr6:34604278:34666118:Ly1;chr6:34605669:34666433:CD20;chr6:34605869:34662635:GM12878;chr6:34618640:34678202:Adipose Nuclei;chr6:34622784:34644104:MM1S;chr6:34623017:34665695:Fetal Intestine Large;chr6:34623068:34642580:CD8 Memory 7pool;chr6:34623117:34642804:CD4 Memory Primary 7pool;chr6:34623194:34649497:CD19 Primary;chr6:34623321:34642750:CD34 Primary RO01536;chr6:34623822:34642005:K562;chr6:34629943:34644374:HepG2
ASHG19SELNC2A100003061	12.491	up	noncoding	ENST00000553410	noncoding	ENSG00000258954	RP11-692C24.1	Reliable	431	chr15:92076121-92078325:+	chr15:92065855:92106662:Brain Hippocampus Middle 150

ASHG19SELNC2A100007995	2.412	down	noncoding	ENST0000031570 7	noncoding	ENSG00000178977	LINC00324	2082	chr17:8123960-8127361:-	chr17:8112579:8127854:HepG2;chr17:8121063:8131239:CD4 Memory Primary 7pool;chr17:8121656:8131346:Small Intestine;chr17:8122481:8127887:HeLa;chr17:8123431:8130749:Gastric;chr17:8123504:8127842:Fetal Intestine;chr17:8123530:8131218:Brain Hippocampus Middle 150;chr17:8123611:8131045:Fetal Intestine Large;chr17:8123637:8131108:Fetal Muscle;chr17:8123803:8131186:Spleen;chr17:8123967:8130884:Lung;chr17:8123971:8131178:Sigmoid Colon;chr17:8123987:8127792:Esophagus;chr17:8124127:8142235:LNCaP;chr17:8124203:8127610:Colon Crypt 2

ASHG19SELNC2A100009235	2.1381	down	noncoding	ENST00000605386	noncoding	ENSG00000271614	LINC00936	3632	chr12:90102736-90106367:+	chr12:90099431:90116571:Tonsil

ASHG19SELNC2A100008019	2.9141	down	noncoding	ENST00000450996	noncoding	ENSG00000223923	AC010136.2	Gold	568	chr2:218843430-218857338:+	chr2:218468439:218528688:HSMMtube;chr2:218506222:218579946:Esophagus;chr2:218509969:218580120:u87;chr2:218548183:218895526:Adipose Nuclei;chr2:218549230:218619480:HSMMtube;chr2:218552362:218620962:Aorta;chr2:218653278:218887396:Aorta;chr2:218653542:218735949:HSMMtube;chr2:218653746:218853837:Stomach Smooth Muscle;chr2:218653989:218894379:Left Ventricle;chr2:218653998:218760935:Lung;chr2:218654003:218875571:Esophagus;chr2:218654007:218725195:Right Atrium;chr2:218654030:218736842:Psoas Muscle;chr2:218654085:218820251:Spleen;chr2:218654112:218736192:Skeletal Muscle;chr2:218654125:218872122:Adrenal Gland;chr2:218660828:218882796:Ovary;chr2:218665357:218753185:Fetal Muscle;chr2:218668946:218724501:HSMM;chr2:218669821:218724987:Duodenum Smooth Muscle;chr2:218679869:218854013:Brain Hippocampus Middle;chr2:218686430:218809140:Osteoblasts;chr2:218692062:218724052:Right Ventricle;chr2:218743526:218876017:Pancreatic islets;chr2:218751029:218889973:Right Atrium;chr2:218752708:218831801:Psoas Muscle;chr2:218754400:218830001:Right Ventricle;chr2:218754638:218814123:Brain Cingulate Gyrus;chr2:218761678:218869783:Gastric;chr2:2187

ASHG19SELNC2A100006330	2.2299	down	noncoding	FTMT22600003935	noncoding	CATG00000096154	CATG00000096154.1	2798	chr7:77055446-77058243:-	chr7:77010620:77111780:CD14;chr7:77016591:77056363:HepG2;chr7:77036386:77095861:u87;chr7:77054037:77097807:HSMMtube;chr7:77054421:77095171:HSMM;chr7:77054423:77094996:Skeletal Muscle Myoblast
ASHG19SELNC2A100004791	2.3453	down	noncoding	FTMT22400000953	noncoding	CATG00000082734	CATG00000082734.1	1360	chr6:11650824-11652183:+	chr6:11584761:11657637:HSMMtube;chr6:11585953:11657271:HSMM;chr6:11585977:11634145:Skeletal Muscle Myoblast;chr6:11604834:11619187:HUVEC;chr6:11647355:11656665:Skeletal Muscle Myoblast;chr6:11648278:11657232:HUVEC
ASHG19SELNC2A100941269	2.8803	up	noncoding	MICT00000351436	noncoding	CATG00000104069	CATG00000104069.1	4239	chr8:129192183-129206123:-	chr8:129147244:129191727:MCF-7;chr8:129153748:129219173:HCC1954;chr8:129158962:129219148:Ly3;chr8:129159080:129218964:HBL1;chr8:129163751:129203247:HSMMtube;chr8:129165089:129210112:u87;chr8:129179300:129198539:HUVEC;chr8:129179447:129215108:Osteoblasts;chr8:129179643:129210014:Skeletal Muscle Myoblast;chr8:129179643:129210119:HSMM;chr8:129179715:129198151:NHEK;chr8:129179902:129198300:NHDF-Ad;chr8:129179996:129198145:HMEC;chr8:129180089:129198014:Astrocytes

ASHG19SELNC2A101470248	2.1892	down	noncoding	ENST0000050497 2	noncoding	ENSG00000230148	HOXB-AS1	680	chr17:46620913-46623449:+	chr17:46614519:46633817:K562;chr17:46616505:46643764:Stomach Smooth Muscle;chr17:46616495:46628430:Duodenum Smooth Muscle;chr17:46616604:46628510:Panc1;chr17:46616612:46638413:IMR90;chr17:46617686:46638459:Fetal Intestine;chr17:46617796:46628498:Astrocytes;chr17:46617893:46635291:CD4 Memory Primary 7pool;chr17:46617957:46626930:CD8 Memory 7pool;chr17:46617962:46634923:CD34 Primary RO01536;chr17:46617995:46638448:Fetal Intestine Large;chr17:46618028:46626453:Fetal Muscle;chr17:46618257:46633041:NHLF;chr17:46618362:46647118:NCI-H69;chr17:46618553:46642514:GLC16;chr17:46618902:46626390:CD34 adult;chr17:46628526:46699689:Panc1;chr17:46631233:46657163:Sigmoid Colon;chr17:46638491:46676520:IMR90;chr17:46638493:46660923:Spleen;chr17:46639530:46679459:Fetal Intestine Large;chr17:46641810:46667072:Adipose Nuclei;chr17:46641815:46655410:Duodenum Smooth Muscle;chr17:46643752:46661293:Esophagus;chr17:46643837:46661670:Stomach Smooth Muscle;chr17:46646373:46671210:NHLF;chr17:46652473:46663178:K562;chr17:46652672:46657163:VACO 400;chr17:46655441:46671367:NHDF-

ASHG19 SELNC2 A100005 499	2.1384	up	noncoding	MICT00 0001985 25	noncodi ng	CATG000000 49907	CATG000000 49907.1	1540	chr2:12789 9385- 127904755: -	chr2:127802089:127916166:Brain Hippocampus Middle;chr2:127802281:127866253:Psoas Muscle;chr2:127802319:127915961:Brain Cingulate Gyrus;chr2:127802471:127866282:Skeletal Muscle;chr2:127802710:127915790:Brain Hippocampus Middle 150;chr2:127802898:127935319:Fetal Muscle;chr2:127806748:127912517:Brain Inferior Temporal Lobe;chr2:127806783:127915950:Brain Anterior Caudate;chr2:127807526:127873345:VACO 400;chr2:127807911:127911516:Brain Angular Gyrus;chr2:127811208:127860003:Colon Crypt 2;chr2:127821031:127865759:HSMMtube;chr2:127821381:127879490:Adipose Nuclei;chr2:127821585:127843293:CD4p CD25- Il17p PMAstim Th17;chr2:127821634:127865515:Adrenal Gland;chr2:127821815:127865860:Sigmoid Colon;chr2:127821868:127886512:Gastric;chr2:127821870:127845265:CD8 primiary;chr2:127822206:127865564:LNCaP;chr2:127824973:127845272:Brain Mid Frontal Lobe;chr2:127837127:127865862:Spleen;chr2:127858009:127875840:Brain Mid Frontal Lobe;chr2:127900679:127934599:Psoas Muscle

ASHG19SELNC2A100001579	2.1183	up	noncoding	ENST0000607951	noncoding	ENSG00000272982	RP5-1180E21.4	Reliable	118	chr1:111723996-111724113:-	chr1:111707642:111732738:CD20;chr1:111720463:111773369:CD4p CD25- CD45RAp Naive;chr1:111720877:111748580:Adipose Nuclei;chr1:111721356:111773351:CD4p CD25- Il17- PMAstim Th;chr1:111721546:111771793:CD8 primiary;chr1:111722467:111770686:CD56;chr1:111722584:111771572:CD3;chr1:111724894:111769353:CD4 Memory Primary 7pool;chr1:111725046:111766110:CD14;chr1:111725074:111771067:CD4p CD25- CD45ROp Memory;chr1:111727706:111778392:DND41;chr1:111730652:111771012:CD8 Naive 8pool;chr1:111730953:111769306:CD4p CD25- Il17p PMAstim Th17;chr1:111731924:111786958:Spleen;chr1:111732684:111775273:Tonsil;chr1:111732689:111766358:Sigmoid Colon;chr1:111732755:111766318:Small Intestine;chr1:111734292:111748221:Gastric;chr1:111734374:111769514:GM12878;chr1:111734461:111748269:Esophagus;chr1:111734723:111748126:Colon Crypt 2;chr1:111734737:111768995:CD19 Primary;chr1:111734752:111768907:CD4p CD225int CD127p Tmem;chr1:111734884:111769467:CD8 Memory 7pool;chr1:111734917:111772142:Fetal Thymus;chr1:111734952:111769864:CD4 Naive Primary 8pool;chr1:111735057:111770041:CD8 Naive 7pool;chr1:111735190:111768994:CD4 Memory Primary 8pool;chr1:111735195:111775083:Ly1;chr1:1117352

ASHG19 SELNC2 A100000 492	13.68	up	noncoding	ENST00 0004194 40	noncodi ng	ENSG000002 38117	AP004372.1	Gold	509	chr11:1317 47574- 131767002: -	chr11:131690473:131763837:Ly4;chr11:131737165: 131795496:Osteoblasts;chr11:131757070:131795305 :Astrocytes

ASHG19SELNC2A100159211	9.1881	up	noncoding	MICT000002605 57	noncoding	CATG00000067637	CATG00000067637.1		3277	chr4:6765235-6776239:+	chr4:6747543:6755479:Sigmoid Colon;chr4:6747688:6775877:Colon Crypt 1;chr4:6747805:6754014:LNCaP;chr4:6747980:6755423:Colon Crypt 2;chr4:6748436:6775971:Esophagus;chr4:6769138:6790306:Spleen;chr4:6769697:6896339:CD14;chr4:6893090:6913819:HepG2;chr4:6893265:6972483:Sigmoid Colon;chr4:6893286:6950427:Small Intestine;chr4:6904027:6966281:Gastric;chr4:6909951:6995625:Fetal Intestine Large;chr4:6909995:6959132:Lung;chr4:6910012:6966350:Adrenal Gland;chr4:6910151:6920583:K562;chr4:6911789:6950465:Duodenum Smooth Muscle;chr4:6916638:6995431:Fetal Intestine;chr4:6916719:6972539:Colon Crypt 1;chr4:6916746:6972431:Colon Crypt 2;chr4:6917822:6945824:Spleen;chr4:6918165:6945833:Thymus;chr4:6985595:7004711:Esophagus;chr4:6992335:7008299:HMEC
ASHG19SELNC2A100011176	2.7347	down	noncoding	ENST00000454877	noncoding	ENSG00000228005	AC020743.2	Reliable	767	chr7:50181136-50182419:-	chr7:50180182:50208003:Ly1;chr7:50180370:50209842:Ly3;chr7:50180593:50207933:DHL6
ASHG19SELNC2A100002759	2.6327	up	noncoding	ENST00000421051	noncoding	ENSG00000226527	AP000289.6		1361	chr21:34484005-34496009:-	chr21:34481477:34546723:H2171;chr21:34482797:34500507:Brain Hippocampus Middle 150

ASHG19SELNC2A100082995	3.6485	down	noncoding	NR_034059	noncoding	ENSG00000173692	PSMD1		3159	chr2:231921578-232037540:+	chr2:231967334:231990673:Duodenum Smooth Muscle
ASHG19SELNC2A100082995	3.6485	down	noncoding	NR_034059	noncoding	ENSG00000173692	PSMD1		3159	chr2:231921578-232037540:+	chr2:232023929:232057846:NHDF-Ad;chr2:232025570:232057244:Adrenal Gland;chr2:232038399:232111292:DHL6;chr2:232045467:232093068:Aorta
ASHG19SELNC2A100014661	2.5403	up	noncoding	ENST00000608517	noncoding	ENSG00000273062	RP11-428K3.1	Reliable	1980	chr1:178693441-178695420:-	chr1:178685662:178773611:CD20
ASHG19SELNC2A101026405	5.3655	down	noncoding	ENST00000558454	noncoding	ENSG00000259504	RP11-352D13.5		421	chr15:69576501-69587661:+	chr15:69583026:69621894:Colon Crypt 1;chr15:69593457:69621946:Sigmoid Colon;chr15:69597309:69621782:Colon Crypt 2

ASHG19SELNC2A100005265	3.1104	up	noncoding	ENST0000515627	noncoding	ENSG00000251169	AC005355.2		1107	chr5:133842243-133844920:+	chr5:133836971:133866999:Panc1;chr5:133837064:133928572:Pancreatic islets;chr5:133837467:133864452:HeLa;chr5:133837686:133864566:HCT-116;chr5:133837691:133864074:VACO 400;chr5:133838361:133905185:CD4p CD25- Il17-PMAstim Th;chr5:133838388:133866725:Fetal Intestine;chr5:133838537:133866603:Fetal Intestine Large;chr5:133838541:133869042:Gastric;chr5:133838572:133916041:Lung;chr5:133838583:133931632:Right Atrium;chr5:133838586:133912623:Small Intestine;chr5:133838591:133931800:Left Ventricle;chr5:133838611:133864360:Sigmoid Colon;chr5:133838659:133907026:Spleen;chr5:133838666:133863953:Colon Crypt 1;chr5:133838667:133863766:Colon Crypt 2;chr5:133838668:133917568:Adrenal Gland;chr5:133838670:133867130:Esophagus;chr5:133838672:133892328:Tonsil;chr5:133838676:133863986:VACO 9m;chr5:133838696:133907397:Brain Hippocampus Middle;chr5:133838963:133866754:Pancreas;chr5:133840955:133864689:HepG2;chr5:133841422:133864268:VACO 503;chr5:133846492:133877168:H1;chr5:133856931:133920988:CD20;chr5:133858546:133892597:CD4p CD25- CD45RAp Naive;chr5:133858888:133892472:HBL1;chr5:133858968:133893355:CD4p CD25- CD45ROp Memory;chr5:133859042:133893012:CD8
ASHG19SELNC2A100009910	3.0236	up	noncoding	ENST0000524499	noncoding	ENSG00000255224	CTD-3065J16.9	Reliable	1264	chr8:145132905-145134168:-	chr8:145100141:145136169:HCT-116;chr8:145103882:145135645:Colon Crypt 3

ASHG19SELNC2A100009257	2.0512	up	noncoding	ENST00000609211	noncoding	ENSG00000273403	RP11-329B9.3	Reliable	526	chr3:184459725-184460250:+	chr3:184274276:184303185:Gastric;chr3:184279295:184303121:Esophagus;chr3:184280246:184445487:Fetal Muscle;chr3:184281212:184287586:LNCaP;chr3:184281285:184322501:Ovary;chr3:184444168:184485359:HMEC;chr3:184454658:184491921:Esophagus;chr3:184455452:184492084:Gastric
ASHG19SELNC2A100009678	4.5334	up	noncoding	ENST00000411847	noncoding	ENSG00000233110	RP11-301L8.2	Reliable	1789	chr4:186509063-186515157:+	chr4:186421060:186464953:Skeletal Muscle;chr4:186421360:186499390:Stomach Smooth Muscle;chr4:186433975:186523178:Aorta;chr4:186479908:186520031:HSMMtube;chr4:186486507:186509539:Fetal Muscle;chr4:186486565:186515249:Skeletal Muscle;chr4:186487403:186511232:HSMM
ASHG19SELNC2A100000421	13.481	up	noncoding	ENST00000432711	noncoding	ENSG00000234919	AC064834.3	Gold	555	chr2:196434352-196437694:-	chr2:196332206:196461723:DND41;chr2:196335194:196360792:Jurkat;chr2:196395093:196445631:CD4p CD25- Il17- PMAstim Th;chr2:196395930:196445779:Tonsil;chr2:196422737:196441571:Ly3
ASHG19SELNC2A100005773	2.051	up	noncoding	ENST00000431729	noncoding	ENSG00000227925	RP11-191N8.2		423	chr1:222001008-222014008:-	chr1:221974668:222061847:u87;chr1:221975004:222043453:HMEC;chr1:221989710:222020004:HSMM;chr1:221989823:222019970:Skeletal Muscle Myoblast;chr1:222058928:222066721:HMEC

ASHG19SELNC2A100219751	3.5648	down	noncoding	ENST0000436293	noncoding	ENSG00000189223	PAX8-AS1	2687	chr2:113993846-114024587:+	chr2:113905849:113962952:CD20;chr2:113913797:113999675:Gastric;chr2:113913871:113962659:CD4p CD25- Il17- PMAstim Th;chr2:113914112:114004833:Pancreatic islets;chr2:113914421:113962467:CD4p CD25- Il17p PMAstim Th17;chr2:113914486:113939384:Colon Crypt 2;chr2:113914618:113956829:Thymus;chr2:113924170:113970102:CD14;chr2:113956031:113984103:VACO 9m;chr2:113956137:114015415:u87;chr2:113974554:114000569:Adrenal Gland

ASHG19SELNC2A100662953	2.0908	down	noncoding	ENST000005867 41	noncoding	ENSG00000267583	RP11-322E11.5	571	chr18:33026872-33047052:-	chr18:33009688:33061581:Osteoblasts;chr18:33035511:33072445:HUVEC;chr18:33035518:33107158:Stomach Smooth Muscle;chr18:33054479:33061134:HMEC;chr18:33055682:33060819:NHEK;chr18:33057530:33094604:Esophagus

ASHG19SELNC2A100006962	3.2291	up	noncoding	ENST0000060559 5	noncoding	ENSG00000271327	RP11-1109F11.3	Reliable	1495	chr12:89761584-89763078:+	chr12:89716946:89750065:Spleen;chr12:89721256:89749426:DND41;chr12:89727363:89749582:MM1S;chr12:89727454:89749549:Small Intestine;chr12:89733216:89785367:Left Ventricle;chr12:89733313:89749397:Fetal Muscle;chr12:89736686:89749897:Adipose Nuclei;chr12:89738766:89749678:Duodenum Smooth Muscle;chr12:89738992:89749161:HUVEC;chr12:89739228:89749898:HCT-116;chr12:89739271:89785574:Sigmoid Colon;chr12:89739274:89749889:Lung;chr12:89739317:89785560:Right Atrium;chr12:89739368:89796395:Fetal Intestine;chr12:89739391:89749746:Gastric;chr12:89739453:89796324:Fetal Intestine Large;chr12:89739456:89749194:VACO 400;chr12:89739987:89749673:VACO 503;chr12:89740269:89749345:NHLF;chr12:89740392:89779192:HMEC;chr12:89742737:89749385:Esophagus;chr12:89744290:89749109:Pancreas;chr12:89744358:89785524:Colon Crypt 3;chr12:89761862:89785888:HUVEC;chr12:89772428:89786699:HepG2

ASHG19SELNC2A100004684	2.5217	up	noncoding	ENST00000605051	noncoding	ENSG00000271259	RP11-1109F11.5	Reliable	540	chr12:89765597-89766136:+	chr12:89716946:89750065:Spleen;chr12:89721256:89749426:DND41;chr12:89727363:89749582:MM1S;chr12:89727454:89749549:Small Intestine;chr12:89733216:89785367:Left Ventricle;chr12:89733313:89749397:Fetal Muscle;chr12:89736686:89749897:Adipose Nuclei;chr12:89738766:89749678:Duodenum Smooth Muscle;chr12:89738992:89749161:HUVEC;chr12:89739228:89749898:HCT-116;chr12:89739271:89785574:Sigmoid Colon;chr12:89739274:89749889:Lung;chr12:89739317:89785560:Right Atrium;chr12:89739368:89796395:Fetal Intestine;chr12:89739391:89749746:Gastric;chr12:89739453:89796324:Fetal Intestine Large;chr12:89739456:89749194:VACO 400;chr12:89739987:89749673:VACO 503;chr12:89740269:89749345:NHLF;chr12:89740392:89779192:HMEC;chr12:89742737:89749385:Esophagus;chr12:89744290:89749109:Pancreas;chr12:89744358:89785524:Colon Crypt 3;chr12:89761862:89785888:HUVEC;chr12:89772428:89786699:HepG2
ASHG19SELNC2A100059370	5.4473	down	noncoding	ENST00000528316	noncoding	ENSG00000248844	RP11-626H12.3		548	chr11:69902336-69910030:-	chr11:69894404:69928230:HCC1954;chr11:69918445:69983019:Aorta

ASHG19SELNC2A100662346	2.7998	up	noncoding	ENST000005904 78	noncoding	ENSG00000266947	RP11-799D4.4	2658	chr17:33558469-33569982:-	chr17:33537241:33598139:CD4p CD25- Il17- PMAstim Th;chr17:33538318:33614200:CD4p CD25- CD45RAp Naive;chr17:33562741:33580128:CD4 Memory Primary 7pool;chr17:33563877:33573407:HMEC;chr17:33564068:33596942:CD8 primiary;chr17:33564704:33575384:CD4p CD25- Il17p PMAstim Th17;chr17:33565216:33584939:HSMMtube;chr17:33565217:33581725:NHEK;chr17:33565325:33572561:CD8 Naive 7pool;chr17:33566773:33572593:CD4 Naive Primary 7pool;chr17:33567989:33572775:CD56;chr17:33568279:33572821:CD4 Naive Primary 8pool;chr17:33568548:33585186:HeLa
ASHG19SELNC2A100001086	3.1813	up	noncoding	ENST000004167 74	noncoding	ENSG00000223823	RP11-465B22.5	1620	chr1:1072397-1079436:+	chr1:1058966:1101877:Small Intestine;chr1:1059412:1101686:Fetal Intestine Large;chr1:1059633:1101903:Fetal Intestine;chr1:1059682:1101913:VACO 400;chr1:1067578:1102178:LNCaP;chr1:1068186:1101896:Esophagus;chr1:1069414:1073756:Colon Crypt 3;chr1:1069443:1073717:Colon Crypt 2;chr1:1070790:1094387:CD19 Primary;chr1:1071352:1103985:VACO 9m;chr1:1077814:1082618:Colon Crypt 3;chr1:1077845:1081465:Colon Crypt 2;chr1:1079431:1101911:HCT-116;chr1:1090819:1107666:Gastric;chr1:1092804:1107737:Colon Crypt 3;chr1:1092807:1104591:Sigmoid Colon;chr1:1092822:1104598:Colon Crypt 1;chr1:1092838:1103486:Colon Crypt 2;chr1:1092849:1101754:Pancreas;chr1:1096780:1105079:Pancreatic islets;chr1:1097418:1104038:VACO 503

ASHG19SELNC2A100015711	2.1593	up	noncoding	ENST00000501178	noncoding	ENSG00000214772	RP11-174G6.1	657	chr12:14818589-14910897:+	chr12:14863465:14868433:H1

ASHG19SELNC2A100015711	2.1593	up	noncoding	ENST00000501178	noncoding	ENSG00000214772	RP11-174G6.1		657	chr12:14818589-14910897:+	chr12:14894156:14929903:Fetal Intestine;chr12:14904251:14929729:Fetal Intestine Large;chr12:14909266:14930627:Gastric;chr12:14920323:14925540:CD4p CD25- Il17p PMAstim Th17;chr12:14920944:14929391:Tonsil;chr12:14920983:14929308:CD4p CD25- Il17- PMAstim Th;chr12:14921125:14929327:CD4p CD25- CD45RAp Naive;chr12:14921171:14925487:CD4 Memory Primary 7pool;chr12:14921199:14929173:CD4p CD25- CD45ROp Memory;chr12:14921305:14925568:CD8 Naive 8pool;chr12:14921398:14925615:CD4 Naive Primary 8pool;chr12:14921454:14925458:CD4p CD225int CD127p Tmem;chr12:14921547:14925520:CD56;chr12:14921553:14929535:Pancreatic islets;chr12:14921581:14925611:CD8 Memory 7pool;chr12:14921659:14925421:CD3;chr12:14921685:14925507:CD8 Naive 7pool;chr12:14921718:14925476:CD4 Naive Primary 7pool;chr12:14921730:14925713:CD4 Memory Primary 8pool;chr12:14921804:14930583:Colon Crypt 1;chr12:14921817:14929490:Pancreas;chr12:14922017:14929433:HeLa
ASHG19SELNC2A100015415	2.3584	down	noncoding	ENST00000606057	noncoding	ENSG00000272354	RP11-307L14.2	Reliable	376	chr5:64316441-64316816:+	chr5:64308152:64339581:Osteoblasts;chr5:64317564:64375123:HBL1;chr5:64323861:64372928:u87

ASHG19SELNC2A100006906	2.0534	up	noncoding	ENST0000056579 7	noncoding	ENSG00000261526	CTB-31O20.2	Reliable	1288	chr19:1874881-1876168:-	chr19:1825637:1883458:HCT-116;chr19:1825803:1876878:LNCaP;chr19:1851043:1898475:CD8 primiary;chr19:1851043:1898511:CD4p CD25-CD45ROp Memory;chr19:1851179:1863643:CD4 Memory Primary 7pool;chr19:1851308:1898081:CD4p CD225int CD127p Tmem;chr19:1851482:1865735:Colon Crypt 3;chr19:1851542:1865663:Toledo;chr19:1851963:1876206:CD34 adult;chr19:1851994:1898577:CD8 Memory 7pool;chr19:1852750:1865843:Sigmoid Colon;chr19:1852897:1857967:CD34 fetal;chr19:1853107:1866012:Spleen;chr19:1853176:1865991:Esophagus;chr19:1853410:1876816:VACO 503;chr19:1854521:1862878:Small Intestine;chr19:1854545:1885395:Brain Cingulate Gyrus;chr19:1854775:1902160:CD56;chr19:1855456:1863700:Colon Crypt 2;chr19:1855501:1877025:NHLF;chr19:1855536:1863748:Colon Crypt 1;chr19:1873820:1898280:CD4 Naive Primary 8pool;chr19:1873834:1898194:CD8 Naive 8pool;chr19:1873851:1898228:CD8 Naive 7pool;chr19:1873897:1898070:CD4 Naive Primary 7pool

ASHG19SELNC2A100006634	2.3286	up	noncoding	ENST00000439325	noncoding	ENSG00000233912	AC026202.3	Reliable	672	chr3:5198590-5229014:-	chr3:5163110:5182088:CD4p CD25- Il17- PMAstim Th;chr3:5163492:5248060:CD14;chr3:5163601:5181308:NHEK;chr3:5198508:5247721:DND41;chr3:5201330:5241760:Ly1;chr3:5201533:5264860:CD20;chr3:5210188:5247435:Toledo;chr3:5218103:5241954:DHL6;chr3:5220834:5241792:Jurkat;chr3:5220882:5242806:Fetal Thymus;chr3:5221044:5257276:MM1S;chr3:5221589:5247834:CD19 Primary;chr3:5221687:5247709:Tonsil;chr3:5221949:5241436:Thymus;chr3:5226827:5248073:CD4 Memory Primary 7pool;chr3:5227381:5241438:CD8 Memory 7pool;chr3:5227416:5260138:CD4p CD25- Il17- PMAstim Th;chr3:5227479:5248256:Ly3;chr3:5228044:5247995:HBL1;chr3:5228309:5242717:CD4 Memory Primary 8pool;chr3:5228420:5242420:CD4p CD25- Il17p PMAstim Th17;chr3:5229272:5244870:NHEK

ASHG19SELNC2A100001569	2.8314	down	noncoding	ENST0000060505 6	noncoding	ENSG00000270210	RP11-373D23.3	Reliable	775	chr2:28648812-28649586:+	chr2:28538547:28583319:Esophagus;chr2:28539422:28647737:Gastric;chr2:28541595:28638254:Psoas Muscle;chr2:28541764:28638743:Skeletal Muscle;chr2:28549794:28661600:Sigmoid Colon;chr2:28550197:28654694:Spleen;chr2:28555017:28580455:Right Atrium;chr2:28555696:28621822:Fetal Muscle;chr2:28560768:28633683:HeLa;chr2:28561410:28660512:Small Intestine;chr2:28564705:28630926:Duodenum Smooth Muscle;chr2:28569985:28654363:CD56;chr2:28570162:28654496:CD8 primiary;chr2:28578132:28632603:Pancreatic islets;chr2:28578249:28632645:Bladder;chr2:28578290:28620321:Fetal Intestine;chr2:28578354:28661392:Colon Crypt 1;chr2:28578385:28638268:Colon Crypt 3;chr2:28578393:28661293:Colon Crypt 2;chr2:28578431:28620644:Fetal Intestine Large;chr2:28578930:28633785:VACO 503;chr2:28581266:28660518:Tonsil;chr2:28598695:28677657:Esophagus;chr2:28599607:28639515:H2171;chr2:28599847:28632500:HMEC;chr2:28600200:28621212:NHEK;chr2:28600287:28647472:Thymus;chr2:28600588:28649314:CD14;chr2:28601601:28648611:CD4p CD25- Il17- PMAstim Th;chr2:28609177:28647539:HUVEC;chr2:28609954:28647368:Osteoblasts;chr2:28611692:28630654:Adipose

ASHG19SELNC2A100002491	2.0331	up	noncoding	ENCT00000316219	noncoding	CATG00000067577	CATG00000067577.1		2375	chr4:3782491-3784865:+	chr4:3766999:3790224:VACO 400
ASHG19SELNC2A100018469	4.3469	down	noncoding	ENST00000595133	noncoding	ENSG00000268164	CTD-2337J16.1	Reliable	619	chr19:54753289-54753907:-	chr19:54750641:54793891:Spleen;chr19:54772520:54794390:CD14

ASHG19SELNC2A100010603	3.4947	down	noncoding	ENST000005102 74	noncoding	ENSG00000245864	CTC-467M3.1	Reliable	463	chr5:87972036-88018648:+	chr5:87942456:87992522:Brain Inferior Temporal Lobe;chr5:87946098:87991815:Brain Hippocampus Middle 150;chr5:87946127:87989956:Brain Cingulate Gyrus;chr5:87946372:87987414:Brain Anterior Caudate;chr5:87950865:87989017:NCI-H69;chr5:87951604:87989109:u87;chr5:87952149:87990686:Brain Angular Gyrus;chr5:87954387:87972999:MM1S;chr5:87954482:87973550:Toledo;chr5:87954635:87990140:Brain Hippocampus Middle;chr5:87954647:87973608:Ly4;chr5:87955130:87976618:Astrocytes;chr5:87955213:87989872:Brain Mid Frontal Lobe

ASHG19SELNC2A100010603	3.4947	down	noncoding	ENST000005102 74	noncoding	ENSG00000245864	CTC-467M3.1	Reliable	463	chr5:87972036-88018648:+	chr5:88018226:88187764:CD20;chr5:88025259:88059632:Ly3;chr5:88103163:88144005:CD34 Primary RO01536;chr5:88107335:88144439:Ly4;chr5:88109240:88133248:HUVEC;chr5:88110445:88130359:MM1S;chr5:88110469:88144375:Ly1;chr5:88110755:88144233:Tonsil;chr5:88113984:88129954:CD34 Primary RO01549;chr5:88114141:88144133:HBL1;chr5:88114708:88142027:CD19 Primary;chr5:88115423:88132852:CD34 Primary RO01480;chr5:88168302:88187406:Tonsil;chr5:88168664:88186011:Brain Angular Gyrus;chr5:88168729:88186655:Brain Hippocampus Middle 150;chr5:88168862:88185897:Brain Mid Frontal Lobe;chr5:88170622:88187193:Duodenum Smooth Muscle;chr5:88170634:88187174:CD34 Primary RO01536;chr5:88171045:88201024:Fetal Muscle;chr5:88171061:88187020:MM1S;chr5:88171083:88209663:Skeletal Muscle;chr5:88176544:88186609:CD34 Primary RO01480;chr5:88176603:88187231:CD34 Primary RO01549

ASHG19SELNC2A100009108	2.5432	up	noncoding	ENST00000532296	noncoding	ENSG00000255306	RP5-901A4.1	Reliable	739	chr11:67792277-67797928:-	chr11:67768822:67784928:Toledo;chr11:67774986:67788833:HeLa;chr11:67775074:67783359:Lung;chr11:67775466:67783160:VACO 503;chr11:67775911:67797063:HSMMtube;chr11:67777185:67797197:HSMM;chr11:67777199:67797183:Skeletal Muscle Myoblast;chr11:67779534:67820405:Pancreatic islets;chr11:67793884:67802150:Adrenal Gland;chr11:67800871:67818895:Pancreas;chr11:67802480:67832882:CD14;chr11:67802716:67809011:Spleen;chr11:67803279:67809641:Lung;chr11:67803491:67811886:Adrenal Gland
ASHG19SELNC2A100013128	3.2006	up	noncoding	ENST00000604818	noncoding	ENSG00000270659	RP11-105N14.1	Reliable	690	chr2:214017694-214018383:+	chr2:213953442:213995415:DND41;chr2:213963111:214031097:CD56;chr2:213965627:214031947:Tonsil;chr2:214001881:214067934:Toledo;chr2:214009200:214055227:Ly1
ASHG19SELNC2A100014285	2.4231	down	noncoding	ENST00000422542	noncoding	ENSG00000228649	AC005682.5		929	chr7:22896147-22901021:+	chr7:22893122:22907712:Osteoblasts;chr7:22893695:22903725:NHEK;chr7:22893734:22901595:HMEC;chr7:22893787:22907736:NHLF;chr7:22893790:22907802:IMR90
ASHG19SELNC2A100010699	2.2386	down	noncoding	ENST00000545706	noncoding	ENSG00000257105	RP11-259O18.4	Reliable	496	chr12:9139771-9149162:-	chr12:9137617:9202267:CD8 primiary;chr12:9139711:9193378:CD4p CD25- Il17-PMAstim Th

ASHG19SELNC2A100012114	2.3561	down	noncoding	ENST0000425916	noncoding	ENSG00000237149	ZNF503-AS2	2024	chr10:77162812-77168738:+	chr10:77153661:77191888:Esophagus;chr10:77154149:77169985:Pancreatic islets;chr10:77154286:77170840:Lung;chr10:77154416:77170741:Gastric;chr10:77154551:77169965:HCT-116;chr10:77154662:77170075:Spleen;chr10:77154714:77169953:Adrenal Gland;chr10:77154737:77169907:Pancreas;chr10:77154799:77169888:Small Intestine;chr10:77154862:77170741:Aorta;chr10:77155248:77170749:Right Atrium;chr10:77156974:77169864:Ovary;chr10:77159193:77192371:u87;chr10:77159256:77169784:VACO 400;chr10:77159866:77169894:VACO 9m

ASHG19SELNC2A101056345	2.3616	down	noncoding	ENST00000440181	noncoding	ENSG00000231160	RP11-617D20.1		2368	chr4:38614322-38666249:-	chr4:38650482:38694503:Spleen;chr4:38655310:38675608:CD8 Naive 8pool;chr4:38655358:38685822:CD8 primiary;chr4:38655367:38677981:CD4p CD25- CD45RAp Naive;chr4:38655398:38687011:CD56;chr4:38655565:38677219:CD8 Memory 7pool;chr4:38655573:38677376:CD4 Memory Primary 7pool;chr4:38655674:38670105:CD8 Naive 7pool;chr4:38655686:38672752:CD4 Naive Primary 7pool;chr4:38655700:38695917:CD4p CD25- Il17- PMAstim Th;chr4:38655730:38669900:CD4 Naive Primary 8pool;chr4:38655747:38677540:Tonsil;chr4:38655760:38677238:CD34 Primary RO01536;chr4:38655772:38675431:CD3;chr4:38655816:38677021:CD34 Primary RO01549;chr4:38655873:38676894:CD34 Primary RO01480;chr4:38662632:38676989:K562;chr4:38662858:38683634:Fetal Intestine;chr4:38662882:38683745:Fetal Intestine Large;chr4:38662929:38689928:Sigmoid Colon;chr4:38662944:38689939:Small Intestine;chr4:38663001:38694604:Stomach Smooth Muscle;chr4:38663011:38677824:Colon Crypt 3;chr4:38663261:38698619:Duodenum Smooth Muscle;chr4:38663312:38677216:VACO 503;chr4:38663363:38689960:Colon Crypt 1;chr4:38663392:38690772:Gastric;chr4:38663529:38685386:Fetal

ASHG19SELNC2A100389849	17.406	up	noncoding	MICT000002605 56	noncoding	CATG00000067637	CATG00000067637.1	2439	chr4:6765235-6776239:+	chr4:6747543:6755479:Sigmoid Colon;chr4:6747688:6775877:Colon Crypt 1;chr4:6747805:6754014:LNCaP;chr4:6747980:6755423:Colon Crypt 2;chr4:6748436:6775971:Esophagus;chr4:6769138:6790306:Spleen;chr4:6769697:6896339:CD14;chr4:6893090:6913819:HepG2;chr4:6893265:6972483:Sigmoid Colon;chr4:6893286:6950427:Small Intestine;chr4:6904027:6966281:Gastric;chr4:6909951:6995625:Fetal Intestine Large;chr4:6909995:6959132:Lung;chr4:6910012:6966350:Adrenal Gland;chr4:6910151:6920583:K562;chr4:6911789:6950465:Duodenum Smooth Muscle;chr4:6916638:6995431:Fetal Intestine;chr4:6916719:6972539:Colon Crypt 1;chr4:6916746:6972431:Colon Crypt 2;chr4:6917822:6945824:Spleen;chr4:6918165:6945833:Thymus;chr4:6985595:7004711:Esophagus;chr4:6992335:7008299:HMEC

ASHG19SELNC2A100009767	7.0038	up	noncoding	ENST00000413991	noncoding	ENSG00000237614	AC073257.2	Gold	528	chr2:121300485-121301902:-	chr2:121284833:121325534:HSMMtube
ASHG19SELNC2A100339723	2.0004	down	noncoding	ENST00000568827	noncoding	ENSG00000260790	RP11-21M24.2		1415	chr16:22386180-22387594:+	chr16:22363239:22393624:Tonsil;chr16:22379688:22387317:HepG2

ASHG19SELNC2A100011317	2.8304	up	noncoding	ENST000004302 44	noncoding	ENSG00000235475	RP11-166O4.5	489	chr7:66800963-66805011:+	chr7:66790827:66810091:Skeletal Muscle

ASHG19SELNC2A100520580	2.2248	down	noncoding	ENST000005326 97	noncodi ng	ENSG00000255399	TBX5-AS1	961	chr12:114846001-114847419:+	chr12:114798893:114890765:Right Atrium;chr12:114806250:114852976:Lung;chr12:114818897:114854353:Left Ventricle;chr12:114833059:114852784:NHLF;chr12:114833160:114869133:IMR90

ASHG19SELNC2A100009330	4.4023	down	noncoding	MICT000002611 93	noncoding	CATG00000067721	CATG00000067721.1		1019	chr4:10171656-10183330:+	chr4:10150980:10186965:RPMI-8402;chr4:10162614:10215242:CD4 Memory Primary 7pool;chr4:10166089:10191275:u87;chr4:10169349:10206849:K562;chr4:10172655:10189291:CD8 Memory 7pool;chr4:10172793:10188615:CD34 Primary RO01536;chr4:10175487:10184651:CD34 adult
ASHG19SELNC2A100007756	3.6861	down	noncoding	ENCT00000083992	noncoding	CATG00000007199	CATG00000007199.1		1074	chr11:120075373-120076446:-	chr11:120039045:120098496:Brain Hippocampus Middle;chr11:120039076:120098002:Brain Inferior Temporal Lobe;chr11:120039099:120097456:Brain Cingulate Gyrus;chr11:120039192:120097878:Brain Anterior Caudate;chr11:120048839:120096728:Fetal Muscle;chr11:120052177:120096634:Fetal Intestine Large;chr11:120052269:120096765:Fetal Intestine;chr11:120052336:120096938:Sigmoid Colon;chr11:120052757:120096965:Small Intestine;chr11:120053289:120089525:HepG2;chr11:120053442:120092967:Colon Crypt 1;chr11:120053527:120093038:Colon Crypt 2;chr11:120053731:120068943:VACO 400;chr11:120078202:120099019:Spleen;chr11:120078247:120097906:Adrenal Gland;chr11:120094297:120131709:Ly4
ASHG19SELNC2A100000758	5.053	up	noncoding	ENST00000596379	noncoding	ENSG00000268307	CTD-2619J13.13	Reliable	459	chr19:58911588-58912046:+	chr19:58896320:58918674:LNCaP;chr19:58896614:58913811:VACO 503

ASHG19SELNC2A100004107	2.0996	up	noncoding	ENCT0000430892	noncoding	CATG00000101226	CATG00000101226.1	1704	chr8:142106897-142108600:+	chr8:142082657:142171039:Colon Crypt 3;chr8:142083234:142142130:Tonsil;chr8:142093547:142137168:Ly4;chr8:142094319:142137181:Ly1;chr8:142095959:142155874:CD20;chr8:142096012:142175600:Spleen;chr8:142096174:142132036:CD19 Primary;chr8:142120798:142140753:Left Ventricle;chr8:142120902:142193195:Lung;chr8:142124717:142141984:HeLa;chr8:142125160:142175502:Sigmoid Colon;chr8:142125197:142171242:Colon Crypt 1;chr8:142125218:142168720:Colon Crypt 2;chr8:142125252:142175373:Gastric;chr8:142126081:142175538:Small Intestine;chr8:142126519:142143523:HUVEC;chr8:142128389:142176025:CD14;chr8:142136636:142142373:NHLF;chr8:142155859:142175599:NHLF;chr8:142157160:142175949:HUVEC;chr8:142189257:142329653:Pancreatic islets;chr8:142203996:142256112:Colon Crypt 3;chr8:142204096:142218264:Colon Crypt 2;chr8:142235785:142323125:Left Ventricle;chr8:142236313:142322683:Lung;chr8:142247913:142257048:CD3;chr8:142273782:142329307:Gastric;chr8:142274002:142298319:Colon Crypt 1;chr8:142274063:142298247:Colon Crypt 3;chr8:142274170:142298019:Colon Crypt 2;chr8:142275078:142322809:Right Atrium;chr8:142276139:142319373:Pancreas
